# Analysis of Dengue Virus Genetic Diversity during Human and Mosquito Infection Reveals Genetic Constraints

**DOI:** 10.1371/journal.pntd.0004044

**Published:** 2015-09-01

**Authors:** October M. Sessions, Andreas Wilm, Uma Sangumathi Kamaraj, Milly M. Choy, Angelia Chow, Yuwen Chong, Xin Mei Ong, Niranjan Nagarajan, Alex R. Cook, Eng Eong Ooi

**Affiliations:** 1 Program in Emerging Infectious Diseases, Duke-NUS Graduate Medical School, Singapore; 2 Computational and Systems Biology, Genome Institute of Singapore, Singapore; 3 Saw Swee Hock School of Public Health, National University of Singapore and National University Health System, Singapore; Yale-NUS College, National University of Singapore, Singapore; Program in Health Services and Systems Research, Duke-NUS Graduate Medical School, Singapore; The Connecticut Agricultural Experiment Station, UNITED STATES

## Abstract

Dengue viruses (DENV) cause debilitating and potentially life-threatening acute disease throughout the tropical world. While drug development efforts are underway, there are concerns that resistant strains will emerge rapidly. Indeed, antiviral drugs that target even conserved regions in other RNA viruses lose efficacy over time as the virus mutates. Here, we sought to determine if there are regions in the DENV genome that are not only evolutionarily conserved but genetically constrained in their ability to mutate and could hence serve as better antiviral targets. High-throughput sequencing of DENV-1 genome directly from twelve, paired dengue patients’ sera and then passaging these sera into the two primary mosquito vectors showed consistent and distinct sequence changes during infection. In particular, two residues in the NS5 protein coding sequence appear to be specifically acquired during infection in *Ae*. *aegypti* but not *Ae*. *albopictus*. Importantly, we identified a region within the NS3 protein coding sequence that is refractory to mutation during human and mosquito infection. Collectively, these findings provide fresh insights into antiviral targets and could serve as an approach to defining evolutionarily constrained regions for therapeutic targeting in other RNA viruses.

## Author Summary

Dengue viruses cause debilitating and potentially life-threatening acute disease throughout the tropical world. While drug development efforts are underway, there are concerns that drug-resistant strains will emerge rapidly. Indeed, many antiviral drugs for other RNA viruses lose efficacy over time as the virus mutates. Here, we sought to determine if there are regions in the dengue virus genome that are constrained in their ability to mutate and could therefore serve as better targets for antiviral drugs. Deep sequencing of the dengue virus 1 genome directly from the blood of twelve dengue patients and from mosquitoes given this blood showed consistent and distinct mutation patterns during infection. Importantly, we identified regions within the viral genome that are resistant to mutation during human and mosquito infection. Collectively, these findings provide fresh insights into potential antiviral targets and could serve as an approach to defining better regions for therapeutic targeting in other RNA viruses.

## Introduction

Dengue, caused by one of four dengue viruses (DENV), is the most important arboviral disease in the world. Recent estimates indicate DENV infects 400 million people annually and over half of the world’s population lives in regions endemic for this debilitating and potentially life-threatening disease [1]. DENVs are single-stranded, positive sense RNA viruses within the Flaviviridae family of viruses and are transmitted from human-to-human in most parts of the world by *Aedes aegypti* and *Aedes albopictus* [2]. The DENV genome is approximately 11kb long and encodes a single viral polyprotein that is then post-translationally cleaved into three structural proteins—the capsid (C), pre-membrane (prM) and envelope (E)—and seven non-structural (NS) proteins, NS1, NS2a, NS2b, NS3, NS4a, NS4b and NS5. The known and suspected functions of these proteins have been reviewed elsewhere [3,4]. The viral coding region is flanked by a short 5’ untranslated region (UTR) and a longer 3’ UTR, both of which have been shown to associate with host factors and form secondary and tertiary structures that are required for viability of the virus [3]. Dengue prevention relies solely on vector control, which in most places has not resulted in sustainable reduction in disease incidence. While vaccine development has made important strides recently, the efficacy against all four DENV serotypes is variable and protection against infection is incomplete [5,6]. An antiviral drug that specifically combats DENV remains a much-needed tool against this global scourge.

Antiviral drug development has mostly focused on compounds targeting conserved regions of the viral genome. Despite such an approach, drug resistance has developed rapidly, particularly for RNA viruses. RNA viruses are indeed notorious for their ability to adapt quickly to selective pressure from the host immune system and/or antivirals [7,8]. This adaptability can largely be attributed to their existence as a population and the error-prone characteristics of their RNA-dependent RNA polymerase (RdRp) [9–11]. These features combine to make RNA viruses able to quickly adapt to selective pressure from the host or antiviral treatment by exploring available sequence space [12–22]. Combination therapy is thus required to prevent rapid emergence of drug resistant strains and this strategy have been successful for human immunodeficiency virus (HIV) and hepatitis C virus (HCV) [23,24]. However, such a therapeutic approach may not be suitable for viruses such as dengue or chikungunya. The cost of treatment would increase with each additional drug and the tropical world, where these viruses are prevalent and cause significant economic burden, may not be able to afford the treatment needed. Identification of regions within the DENV genome that are not only evolutionarily conserved but also genetically constrained could thus pinpoint potent and resilient targets for monotherapy that minimizes risk of resistance emergence.

**Fig 1:**
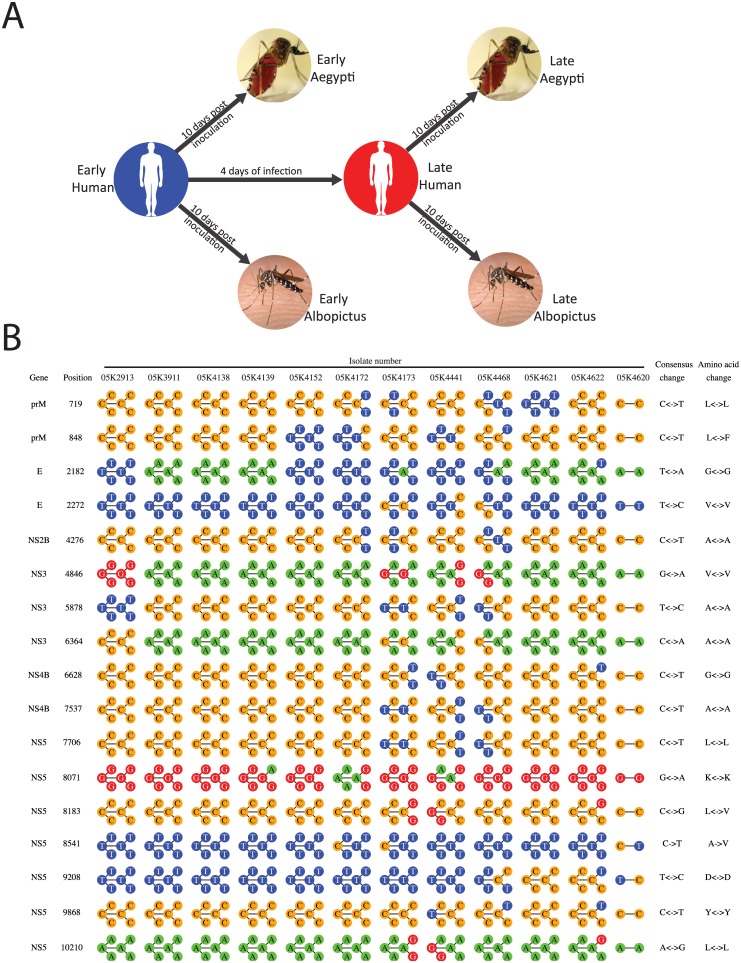
Consensus shifts over time by position. (**A**) Serum samples were taken from patients on fever day 1–3 (Early Human) and again four days later (Late Human). These sera were inoculated into *Ae*. *aegypti* and *Ae*. *albopictus* and sampled 10 days later. (**B**) Positions evidencing a change in consensus sequence in at least a quarter of our samples were recorded and interrogated for each DENV-1 strain. Nucleotide sequences are color-coded and consensus is defined as >50% of reads mapping to the indicated position. Each of the circles depicted represents an experimental condition and are in the order of the experimental design figure depicted above.

To this end, we analyzed intra-host genetic diversity of DENV1 at day 1–3 and again at 4–7 following onset of fever in 12 dengue patients. The sera from these patients were then intra-thoracically inoculated into both *Ae*. *aegypti* and *Ae*. *albopictus* and analyzed after 10 days of infection (Fig 1a). This method of viral delivery to the mosquito bypasses the bottlenecking event the virus encounters in the midgut barrier [25] and was necessary due to the limited amount of patient sera available. This method does, however, allow us to explore the full mutational space available to the virus when not confronted by this bottleneck thereby allowing a more complete picture of which areas in the genome tolerate a degree of variability without sampling hundreds of natural infections. Conversely, we were also able to identify those regions where variability was significantly reduced. These areas of reduced variation, hereby referred to as constrained, likely represent residues lethal to the virus if mutated. Using the resolution enabled by next generation sequencing (NGS) technologies [12], we show that there is an abundant accumulation of intra-host viral population diversity in both humans and mosquitoes. Unexpectedly, we observed specific variations in the DENV genome in *Ae*. *aegypti* not present in *Ae*. *albopictus*, suggesting that amid the stochastic variations, there are distinct changes critical for DENV to thrive in each mosquito host. Importantly, we also show that there are regions of constraint within the viral genome that are refractory to variation in both human and mosquito.

## Results

The intra-host genetic diversity of DENV1 was analyzed in 12 individuals that were enrolled in the early dengue infection and outcome (EDEN) study (S1 File) [26,27]. Consensus sequences of DENV1 isolated from these 12 individuals and grown in C6/36 cells have been reported previously [28]. DENV1 genomic material from paired serum samples was also taken at fever day 1–3 (early) and 4–7 (late) from each patient. The patients were a mixture of primary and secondary infection and the final diagnosis for each of them are shown in S1 File [28]. We tested for differences between primary and secondary infections using the non-parametric Wilcoxon-Mann-Whitney test but none were statistically significant. DENV1 genomic material from these samples was PCR amplified and deep sequenced. These same serum samples were also inoculated intrathoracically into 4-day old female *Ae aegypti* and *Ae albopictus*. After 10 days of incubation, the ten mosquitoes for each serum sample were pooled to minimize sampling bias from individual mosquitoes. DENV1 was then PCR amplified from the total RNA and deep sequenced on an Illumina or Solid sequencing platform (Fig 1a and S2 File). In order to check whether different sequencing technologies (Solid and Illumina) had an effect on the type of SNPs detected, we performed Fisher's exact test on the number of Transition and Transversion SNPs of serum samples sequenced by Solid and Illumina. The results of this analysis suggest that there are no significant differences in any gene between Solid and Illumina sequencing (S3 File).

Overall, our deep sequencing data shows positional variance throughout the DENV1 genome (S4, S5 and S6 Files). The 17 positions where consensus discordance was observed in at least three of the twelve viruses are shown in Fig 1b and the complete list of all consensus changes observed in our data set are described in S7 File. These consensus changes fall within the coding sequence and the 3’UTR. Two of these consensus changes, one in prM and the other in NS5 also resulted in changes to the protein coding sequence.

Besides the limited number of consensus changes, there are a large number of positions throughout the viral genome that display a degree of intra-host viral diversity. We refer to these types of positions as having ‘variance’. To distinguish variants from the average sequencing error rate, we used the program Lofreq, which identifies single nucleotide polymorphisms by incorporating base-call quality scores as error probabilities into its model and assigns a p-value to each variant [29]. These analyses identified seven positions within the DENV genome that possessed this level of reproducible plasticity: two in the E gene, one in the NS1 gene, one in the NS3 gene, one in the 2k peptide at the C terminus of the NS4a gene and two in the NS5 gene (S4 File).

**Fig 2:**
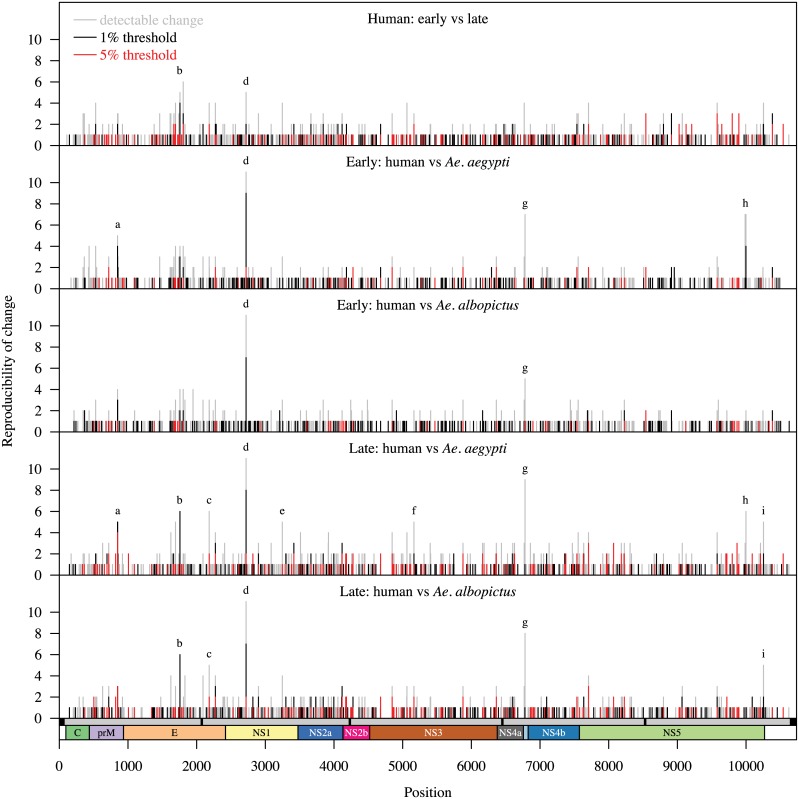
Examination of intra-host genetic diversity by position over time. The DENV genome of each strain was analyzed for positions having detectable, >1% and 5% non-consensus base calls. The number of strains with detectable (grey), >1% (black) and >5% (red) variance are plotted on the y-axis for each position in the DENV genome (x-axis) This analysis was completed for each of the five time periods tested: From Early Human to Late Human, From Early Human to *Ae*. *aegypti* and *Ae*. *albopictus*, and Late Human to *Ae*. *aegypti* and *Ae*. *albopictus*. The letters indicate loci with a high degree of reproducibility (greater than 1/3 of the samples, corresponding to a *p*-value <10^−7^). The residues at each locus are: 848 and 854 (a), 1691, 1757 and 1804 (b), 2182 (c), 2719 (d), 3247 (e), 5164 (f), 6782 (g), 9986 and 9998 (h) and 10253 (i).

Since our samples were extracted from the same patients at two time points during their infection and then directly inoculated into the two mosquito vector species, we were able to track these changes in the genetic diversity across the viral genome over time. More specifically, we compared the proportion of base calls at each position in the DENV genome in early and late serum samples as well as between human and *Ae*. *aegypti* or *Ae*. *albopictus* in both early and late stages of acute dengue. Our results indicate that during the course of the human infection, changes in the intra-host genetic diversity were more prevalent in the NS1, NS2A and E genes (NS2A vs NS2b Bonferroni corrected p-value [Bcp] = 0.008; NS2A vs NS3 Bcp<0.001; NS2A vs NS4B Bcp = 0.02; NS2A vs NS5 Bcp<0.001; E vs NS3 Bcp = 0.006; also NS1 vs NS3 Bcp = 0.002). The average number of changes occurring over the course of four days of human infection is 86 or ~0.0020 changes/position/day of human infection. In *Ae*. *albopictus*, changes were observed in E, NS1, NS4A (2k peptide) and NS5 genes. In *Ae*. *aegypti*, changes were observed in prM, E, NS1, NS3, NS4A (2k peptide) and NS5 genes (Fig 2 and S5 File). Two of the most commonly observed changes at 2719 (NS1) and 6782 (2k peptide) were observed in both species of mosquito and suggests that selection pressure on these residues is likely to be a common mechanism shared between the species. Interestingly, there were changes that were unique to *Ae*. *aegypti* infection, notably at 9986 and 9998 in the NS5 gene. These changes suggest that differential selection pressures may be applied on selected nucleotide residues in the DENV genome by *Ae*. *aegypti* but not by *Ae*. *albopictus*.

**Fig 3:**
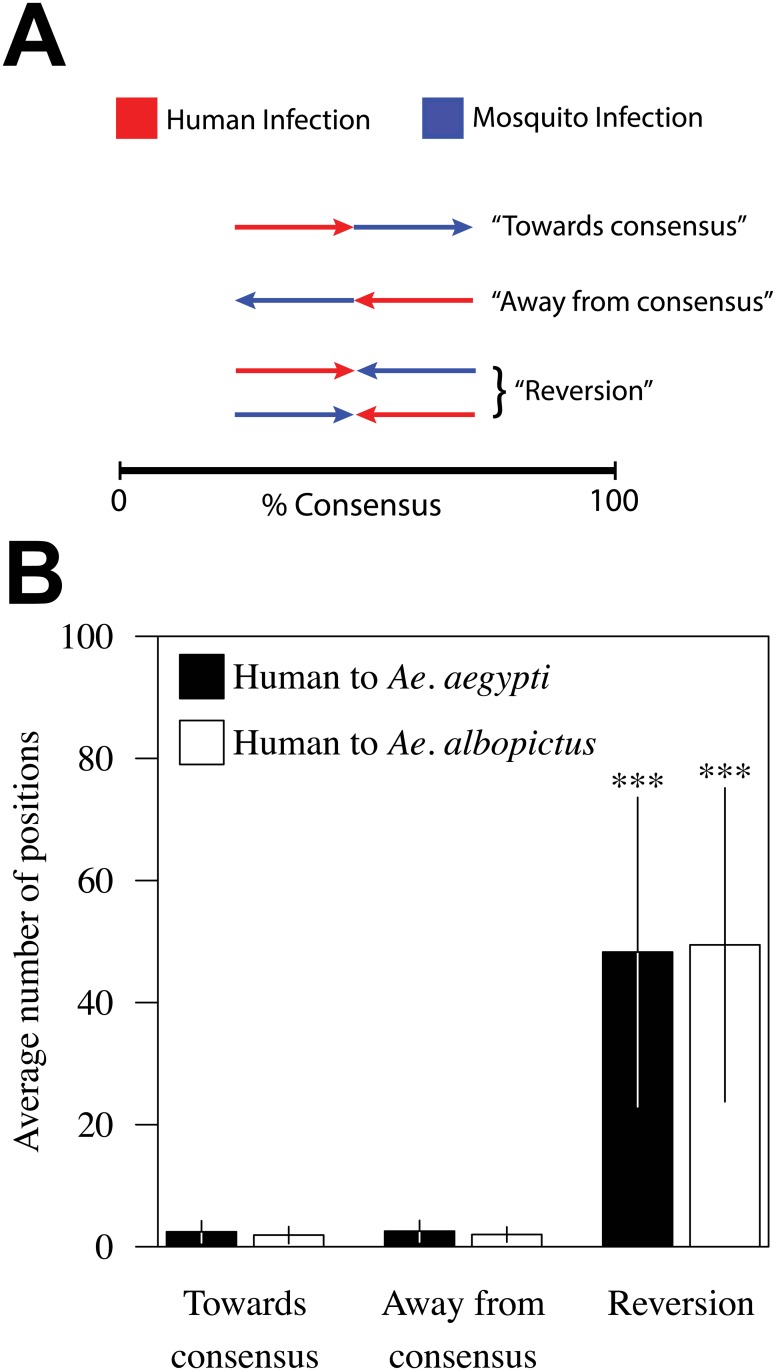
Direction of mutation during infection in human and mosquitoes. (**A**) The change in non-consensus base calls was calculated for each position in the DENV genome over the course of infection in human and in mosquito. For each position, the direction of the change in human and mosquito, either towards consensus or away from consensus, was recorded. If a position that was moving either towards or away from consensus during the human infection continued on this same trajectory during the course of the mosquito infection, than that position is categorized as “Towards consensus” or “Away from consensus”, respectively. If the directions of change differed during human and mosquito infection, than these positions are categorized as “Reversion”. (**B**) The average number of positions falling into each of the three categories listed above is indicated. *** indicates a p-value of <0.01.

For each detectable change in the DENV genome over the course of the human infection, we defined whether the proportion of base calls at each position moved towards or away from the consensus base after 10 days of incubation in the vector. Our results indicate that the variance acquired between early and late serum samples undergo a reversion back towards the sequence in the early serum sample after 10 days of incubation in either vector (Fig 3). We then asked whether this reversion was happening in specific regions or was a more general mechanism. Our data suggest that reversion is largely a general phenomenon that occurs across the majority of the viral genome (S8 File). The majority of the reversion events are small oscillations in the overall composition at each position; however, larger consensus-level changes were also observed (S9 File).

Selection pressure and genetic drift were measured by calculating the dN/dS ratio. Overall it can be seen from the mean dN/dS ratio for each group that there is likely a purifying selection pressure against non-synonymous mutations (S10 File). The Mann-Whitney test was used to compare single protein coding sequences against the rest of the polyprotein and results suggest that there are no significant differences within the human samples (Early and Late), whereas significant differences were identified within the *Ae*. *aegypti* and *Ae*. *albopictus* samples (S11 File). The transition/transversion analysis and the Shannon diversity index and Shannon equitability measurements suggest that there is a decrease in mutation frequency from early to late samples in *Ae*. *aegypti* and human whereas an increase in the mutation rate was observed in the *Ae*. *albopictus* samples (S12 and S13 Files respectively). We also questioned whether the two time points for each species were likely to come from the same population and results suggest that the dN/dS ratio is significantly different between the *Ae*. *aegypti* early and late samples (S14 File). From the Ts/Tv ratio comparison, the ratio is significantly different (<0.01) between the Early and Late samples in *Ae*. *aegypti*, *Ae*. *albopictus* and human. This trend is consistent with the results from the Shannon diversity index and Shannon equitability measurements (S13 File).

**Fig 4:**
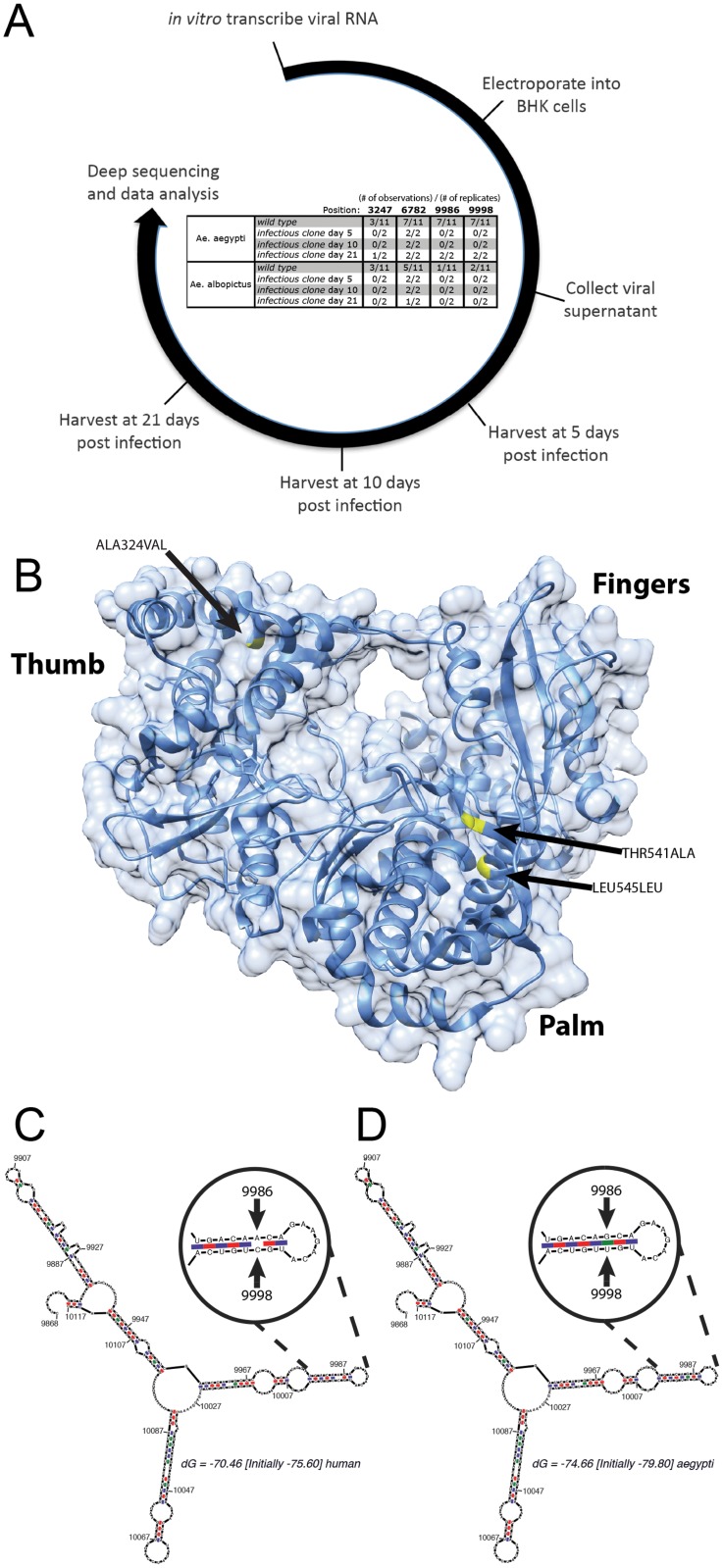
*Ae*. *aegypti* specific mutations in NS5. (**A**) DENV1 genomic RNA was *in vitro* transcribed from an infectious clone and inoculated into mosquitoes. Viral RNA was collected from duplicate pools of ten whole mosquitoes at 5, 10 and 21 days post inoculation and viral populations were analyzed. The time course of the experiment is demarcated on the circular timeline. The number of times we detected significant change at each position in the virus from human sera (wild type) or the virus derived from infectious clone (infectious clone) after incubation in mosquitoes is recorded in the table within the timeline. (**B**) The consensus change observed at position 8541 corresponding to amino acid 324 and the *Ae*. *aegypti* specific mutations in NS5 at position 9986 and 9998 which, correspond to amino acids 541 and 545 are highlighted on the crystal structure of the NS5 protein from Yap et al. 2007 [30]. (**C**) The secondary structure of region surrounding nucleotides 9986 and 9998 was modeled using mFold [31]. (**D**) The nucleotide changes observed in *Ae*. *aegypti* were added into the structure prediction.

The heterogeneity observed at positions 9986 and 9998 (NS5) in the DENV genome fall within the RdRp domain of NS5 and correspond to amino acids 541 (Thr → Ala) and 545 (Leu → Leu) at junction of the “palm” and the α14 alpha helix “finger” of the RdRp domain of the protein, respectively (Fig 4) [30]. That these observations were unique to *Ae*. *aegypti* suggests that these are not random events but are responses to species-specific selection pressure. To test this possibility experimentally, we constructed an infectious clone of DENV1 isolated from the same outbreak in Singapore in 2005 [26] but from a patient not among the 12 studied here. This infectious clone was constructed with the exact nucleotide sequence of the virus (GenBank: EU081230) that was isolated in the C6/36 *Ae*. *albopictus* derived cell line [26]. *In vitro* transcribed RNA was electroporated into BHK cells and harvested supernatant was inoculated intrathoracically into both *Ae*. *aegypti* and *Ae*. *albopictus*. The initial starting material and time points of 5, 10 and 21 days post intrathoracic inoculation were then sequenced and the data analyzed in the same manner as described above. Although the number of replicates in this experiment is limited, our results nevertheless indicate that the changes observed in NS1 and the 2k peptide (Fig 2) are recapitulated in both *Ae*. *aegypti* and *Ae*. *albopictus* (Fig 4). The two *Ae*. *aegypti* specific residues in the NS5 gene, 9986 and 9998 (amino acid positions 541 and 545), arose 21 days after the infectious clone-derived virus was incubated in *Ae*. *aegypti* but not in *Ae*. *albopictus* (Fig 4). Collectively, these findings demonstrate that species-specific selective pressures act to select for variance in specific positions on the DENV genome.

The “palm” domain of the DENV RdRp is the most structurally conserved domain among all known polymerases [30]. Although there is no specific catalytic activity associated with residue at position 541, the Thr→Ala substitution may alter the angle of the “finger” relative to the “palm” and by doing so; alter the enzymatic properties of the RdRp. The nucleotide change at position 9998 does not translate to an amino acid shift at position 545 and its functional significance in regards to RdRp activity is not clear. However, examination of the predicted RNA secondary structure in this region suggests that nucleotides 9986 and 9998 interact with each other in a previously uncharacterized stem-loop structure (Fig 4C and 4D) [31]. The observed A→G and/or C→U changes at bases 9986 and 9998 respectively are predicted to strengthen this interaction (see reduced dG in MFE structure). Other structures and sequences within the virus have been shown to be essential for the virus in a species-specific manner and this may be another mechanism the *Ae*. *aegypti* vector uses to control DENV replication [32].

**Fig 5:**
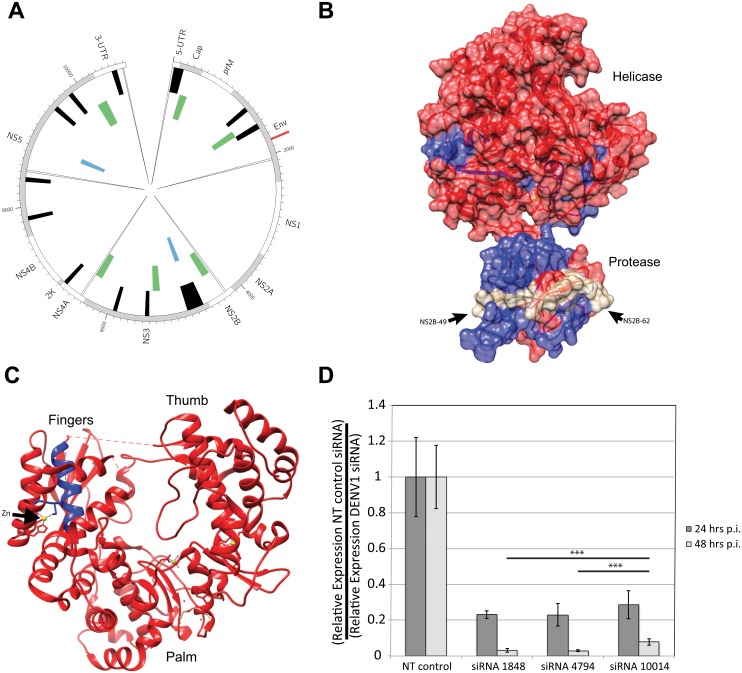
Analysis of mutational coldspots. (**A**). A mutation hotspot/coldspot analysis was performed on the results from the *in vivo* samples and on the infectious clone experimental results. The most outer ring shows gene names and borders. The next track (black) shows coldspots in the mosquito samples. In green are coldspots from the infectious clone samples. The most inner track (blue) shows coldspots in the human samples. There was one hotspot detected (red) in the infectious clone experiment in the E gene (1789–1814). The six large spikes correspond to primer positions. (**B**) The protein structure for NS3 from Luo et al. 2010 [34] is shown with the coldspots depicted in blue. Together these coldspots cluster around the ATP-binding domain where the ADP-Mn^2+^ is depicted in yellow. The NS2B residues (amino acids 49–62; depicted in green) are shown interacting with the protease domain of NS3. (**C**) The ribbon representation of the RdRp is shown with the coldspot depicted in blue with the zinc ion depicted in yellow. (**D**) siRNA’s were designed to target the hotspot (siRNA 1848), the coldspot in NS3 (siRNA 4794) and a region near the Ae. aegypti specific mutations in NS5 (siRNA 10014). A non-targeting (NT control) was used as a control. DENV1 genome was measured relative to GAPDH at 24 and 48 hours post infection and values plotted are fold change relative to the NT control. Error bars indicate the standard deviation of five biological replicates. All DENV1 siRNA’s were significantly different than the NT control (p<0.001) at both 24 and 48 hours post infection. *** indicates p<0.001.

To identify regions of constraint within the DENV1 genome, we aggregated all predicted single nucleotide variants (SNVs) to detect regions with (i) a local enrichment in intra-host SNV calls (mutational hotspots) and (ii) a significant depletion in variants (mutational cold-spots) (Fig 5). This type of analysis complements classical approaches of finding evolutionarily conserved regions through multiple sequence alignments and can reveal functionally important, though otherwise not easily detectable regions [29]. No hotspots predicted in more than one sample of either the mosquito or the human isolates could be detected. However, at least four samples of the infectious clone experiment were observed to have a hotspot in the envelope protein (bases 1789–1814). As the virus used in the infectious clone experiment was *in vitro* transcribed and electroporated into BHK cells for packaging we suggest that this particular hotspot can be attributed to the markedly different selection pressures in cell culture conditions than encountered by the virus *in vivo* [33]. Overall the mosquito samples had 12 coldspots covering 1064 total positions whereas the human samples show only 2 coldspots covering 220 total positions, which is consistent with previous reports [29]. The absence of coldspots in NS1 and NS2A has been observed before [29], but its significance is unclear. Mosquito, human and infectious clone samples largely display an absence of shared mutational coldspots (i.e. regions that show intra-host constraint) with the notable exception of coldspots within the multifunctional NS3 gene (Fig 5). NS3 is comprised of a protease domain and an ATP-driven helicase with two subdomains. The large coldspot discovered in the mosquito samples covers all three of these domains. Structurally, this region clusters around the ATP-binding domain and around the interaction site with NS2B (Fig 5b) [34]. The coldspot in NS5 is in the fingers domain of the RdRp and forms part of the zinc-binding pocket (Fig 5a and 5c) [30]. Although the function of this zinc-binding pocket is unknown, it is a feature shared with the West Nile virus RdRp and is likely to be functionally important [34].

In order to test the hypothesis that coldspot regions would make good antiviral targets, siRNA’s were designed to target the hotspot in the E gene (starting at position 1848), the coldspot in NS3 (starting at position 4794) and a region near the *Ae*. *aegypti* specific mutations in NS5 (starting at position 10014). A non-targeting (NT control) was used as a control for the experiment and DENV1 genome copies relative to GAPDH were measured by RTPCR at 24 and 48 hours post infection. All DENV1 siRNA’s were significantly different than the NT control (p<0.001) at both 24 and 48 hours post infection (Fig 5d). The siRNA’s targeting the E gene and NS3 were indistinguishable from each other in their effect at both 24 and 48 hours post infection. Interestingly, although the siRNA targeting NS5 that contains the *Ae*. *aegypti* specific mutations was not statistically significant from the E gene and NS3 siRNA’s at 24 hours post infection, it was statistically less effective by the 48 hour timepoint (p = 0.0008 and p = 0.0003 respectively) (Fig 5d).

## Discussion

In this study, we have used high-throughput parallel sequencing to analyze intra-host genetic diversity in the DENV genome directly from serum samples obtained from dengue patients and intra-thoracically infected mosquitoes. Our data show that numerous positions within the DENV genome exhibit a high degree of plasticity over the course of infection within the human and mosquito hosts and that several of these changes are in functionally significant domains of the viral coding sequence.

Maintenance of genome plasticity within viral populations is a poorly understood process however; it is possible that it may be critical for the overall fecundity of the virus [9]. Interestingly, members of the mosquito-borne clade of flavivirus have been observed to be more genetically stable over time than other RNA viruses [10,33,35–39]. Previous studies have found that repeated passage in a single host is likely to result in a consensus genome that is highly divergent from its original source [40,41]. In studies measuring viral variance by clonal analysis of viral amplicons, significant intra-host variation of the virus has been observed in laboratory passaged DENV and other flaviviruses such as West Nile virus [33,36,37,42,43]. Serial *in vitro* or *in vivo* passages in mosquitoes show significant amounts of consensus changes throughout the genome [15,33,37,44]. The long-term stability of these viruses may therefore be due to differential selection pressures exerted by the human and mosquito hosts that result in a net conservation of the viral genome [33,37]. Our results suggest that there is an abundant accumulation of intra-host viral population diversity in humans and mosquitoes. Consistent with the prevailing theory however, our study indicates that the changes that accrued during infection in the human host predominantly revert back to the ‘original state’ as the virus transits through the mosquito. Intriguingly, this reversion is occurring despite bypassing the bottlenecking event of midgut barrier escape. This suggests that even when these humanized variants are given the opportunity to replicate in the mosquito body, they are outcompeted by the original population observed early in the human infection. Given the broad distribution across the viral genome and their stochastic appearance in our data set, these larger changes likely represent changes to locations tolerated in the human host but not in the mosquito vectors. The proportion of these variants that are then able to disseminate through the salivary gland infection/escape barrier and thus infect the next human host is also of interest and will be the subject of future study [45]. Taken together, these data provide evidence that cycling between the two hosts restricts the overall diversity in DENV genome, making it phylogenetically more stable than other RNA viruses that propagate within a single species.

Our study also cautions on performing phylogenetic analyses on consensus sequences alone; especially those derived from serially passaged virus. We have also identified species-specific variations that have not been previously reported. Some of these variations are recurrent among the samples we have tested suggesting that there are positions with a high degree of plasticity in the DENV genome. The locations of these positions appear to depend on the host, which provides new insights into how the different vectors may influence DENV evolution. These differences also suggest that viruses transmitted predominantly in an *Ae*. *aegypti*-human cycle may produce viruses genetically distinct from those transmitted predominantly in an *Ae*. *albopictus-*human cycle. Indeed, it may be a molecular basis for which epidemic emergence is more often associated with *Ae*. *aegypti* than *Ae*. *albopictus* [46]. Further studies are necessary to determine whether these same residues arise after passing through the midgut barrier and are ultimately present in the saliva of the infected mosquitoes [47,48].

Interestingly, our samples did not display the extensive mutations in the 5’ or 3’UTR that have been identified in recent studies [49–51]. We observed only a single position in the 3’UTR to change consensus and only for two of the isolates (S7 File). This consensus change falls within the unstructured region between DB1 and DB2 and is not predicted to have a substantial impact on the overall 3’UTR structures (S15 File). The reasons for the observed stability in this region are unclear. The aforementioned studies were primarily conducted in cell lines with DENV2 and, to a lesser extent, DENV3 [49–51]. Whether the DENV1 serotype is fundamentally different in this regard or whether this can simply be attributed to our limited sample size is an interesting question and deserving of additional studies.

The finding of areas within the DENV genome that are constrained in nucleotide variation in the human and mosquito hosts are interesting. These cold-spots are consistent even in two disparate host species and thus suggest that these positions may encode protein-protein interactions that are functionally vital to the DENV lifecycle. The cold-spot at the interface between NS2b and NS3 is particularly interesting. NS3 requires a direct interaction with NS2b as a cofactor for its proteolytic activity. Our findings suggest that if not outright lethal, mutations within this interaction site are likely to cripple the virus. Given that sequence in this region of the genome is highly constrained, a potentially attractive antiviral strategy may be RNA interference (RNAi) due to its potential for high specificity to the viral genome [52,53]. In this study, we tested three different siRNA’s targeting the E gene, NS3 and NS5. Although all were able to significantly reduce viral copy number, the siRNA targeting NS5 was not as effective as the other two after the first 24 hours of infection. Given the plasticity observed in this region when these isolates were passaged into *Ae*. *aegypti*, this may represent a ‘flexible’ part of the genome and a less than ideal target for this type of antiviral strategy. This may indeed also explain the lack of difference between siRNA that targeted the E gene compared to NS3 as, while there is a degree of variance in the E gene, it is not a ‘flexible’ part of the genome that alters depending on the host species. Indeed, it is possible that sub-therapeutic doses of siRNA against the E gene would be more likely to generate resistant mutants over repeated passages compared to that against the cold spot in NS3. This could be a useful focus in future investigations.

While we have used siRNA in this study, small molecule therapeutics against the cold spot on NS3 would be another option. Binding to either interaction surface should interfere with the catalytic function of the viral protease although our data suggests that the NS3 is a more constant target. A small molecule inhibitor would also have the potential advantage of inexpensive mass production; a distinct advantage for the treatment of affected populations unable to afford more expensive therapies.

The changes observed in the PrM and NS5 genes are remarkable as they are highly conserved across most flaviviruses [30,54–56]. The Leu → Phe mutation in the prM gene at position 138 occurs in the C-terminal transmembrane domain of the “M” residue that is embedded in the lipid bilayer of the mature virion [54]. The functional significance of this particular amino acid change is not immediately clear. The membrane composition of mosquito cells are substantially different from mammalian membranes, particularly in their cholesterol content [57]. It is conceivable that an alteration at this position may be in response to these differences and plays a role in the infectivity of the virus. The Val → Ala mutation at position 324 in the NS5 gene occurs at the N-terminus of the RdRp domain in a region involved in the binding of β-importin and NS3 [30]. Alteration in the ability of NS5 to interact with these proteins could directly impact the ability of NS5 to shuttle into the nucleus and the ability of the virus to replicate its genome respectively [30,58]. The lack of extensive cold-spots in the NS5 gene was surprising to us although this might be due to the fact that we pooled ten mosquitoes inoculated with the same serum together. While this methodology has the advantage of being more rigorous when trying to identify common variants, it may obscure authentic coldspots due the averaging effect of combining individuals with stochastic mutations in these regions. One cold-spot was identified in the fingers subdomain of NS5 though not in the thumb or palm domain, which contains the catalytic active site. The latter has been the focus of attention in anti-dengue drug development [59,60]. Furthermore, that we also found variance in two nucleotides in the RdRP domain, one of which changes the protein coding sequence, in DENV that replicated in *Ae*. *aegypti* but not *Ae*. *albopictus* also raises additional concerns on antiviral drug development efforts that target the RdRP. Most laboratories culture DENV in C6/36 cell line, which is derived from *Ae*. *albopictus*. Compounds that show attractive efficacy to DENV cultured in such cells may thus not achieve anticipated efficacy in humans who acquire infection from *Ae*. *aegypti*, which is the epidemiologically more important vector.

Finally, the analysis we have employed in this study can readily be adapted for other pathogen-host studies affecting the developing world such as influenza, chikungunya and ebola viruses. We suggest that our approach could serve not only to identify areas of constraint in viral genomes but also to monitor the emergence of escape mutants following vaccination or initiation of antiviral therapies [61].

## Materials and Methods

### Ethics statement

The samples used in this study were collected under the Early Dengue infection and outcome study (EDEN). This prospective study was approved by the National Healthcare Group Domain Specific Review Board (DSRB B/05/013) and the Institutional Review Boards of the National University of Singapore and DSO National Laboratories. Enrollment of participants into the study was conditional upon written informed consent administered by a designated research nurse. All biological specimens collected for this study were de-identified following collection of demographic and clinical data.

### DENV-1 infection in *Ae*. mosquitoes

Both *Ae*. *aegypti* and *Ae*. *albopictus* mosquitoes were obtained from a colony at the Duke-NUS Graduate Medical School. The colony was established in 2010 with specimens collected in Ang Mo Kio, Singapore, and infused monthly with field-collected mosquitoes to maintain genetic diversity. Female mosquitoes, three to five days old, were intrathoracically inoculated with 0.017 μl of serum from the Early Human sample (fever day 1–3) and the Late Human sample (taken four days after the initial sample) for 11 out of 12 patients as previously described [62]. Insufficient sera remained from one patient for the mosquito inoculations. Mosquitoes inoculated with DENV-1 clinical serum were incubated for 10 days, while mosquitoes inoculated with DENV-1 derived from pOEEic infectious clone were incubated for 5, 10 and 21 days respectively at 28°C and 80% humidity, with access to 10% sucrose and water. Surviving mosquitoes were killed by freezing and examined for the presence of viral antigens in head tissue by direct immunofluorescence assay (IFA). Infected mosquitoes were stored at -80°C until assayed. For each viral sample, including each time point of the infectious clone experiment, 10 infected mosquitoes were pooled and triturated with a pellet pestle (Sigma Aldrich, St. Louis, MO, USA) in 250 μl of 1x PBS. DENV RNA was extracted from the sample using TRIzol RNA isolation reagent (Invitrogen, Carlsbad, CA, USA) according to the manufacturer's protocol and stored at −80°C until use.

### EDEN DENV1 sequencing

A pool of 10 inoculated mosquitoes was triturated in 250ul L-15 (Gibco, Life Technologies, Carlsbad, CA, USA) maintenance medium. Total RNA was extracted using TRIzol® (Life Technologies) according to the manufacturer’s protocol and stored at -80°C until use. Two separate reverse transcription reactions were carried out using the SuperScrip III First-Strand Synthesis System (Life Technologies) according to the manufacturer’s protocol (1) cDNA was synthesized with random hexamers for downstream amplification of fragments 1, 2, 3 and 4. (2) cDNA was synthesized with 10μM of reverse primer 10693R for downstream amplification of fragment 5. cDNA was amplified in 5 fragments by PCR (S16 File). Using the respective primers for each fragment, PCR was carried out using Phusion High-Fidelity PCR Master Mix with HF Buffer (Thermo Fisher Scientific, Waltham, MA, USA). 2μL of cDNA was mixed with 1μL of each primer (10μM), 25μL of 2X master mix and 21μL of water. The PCR conditions were: 30sec at 98C followed by 40 cycles of PCR at 98C for 10sec, 55C for fragment 1 or 57C for fragments 2,3,4 and 5 for 20sec, 72C for 2min and final extension at 72C for 10min. The PCR products were run on a 1.5% agarose gel. Bands of the correct size were excised and gel purified using Qiagen QiaQuick gel extraction kit (Qiagen, Valencia, CA, USA) according to the manufacturer’s protocol. NGS libraries were constructed according to the methods described in Aw et al. [63].

### Production of DENV-1 infectious clone

pOEEic was digested with SacII at 37°C for 2 h. Linearized DNA was purified using ultrapure Phenol: Chloroform: Isoamyl Alcohol (Invitrogen) according to the manufacturer’s protocol. *In vitro* transcription of the purified DNA was performed to generate full-length genomic DENV RNA using MEGAscript T7 kit (Ambion, Life Technologies, Carlsbad, CA, USA) according to the manufacturer’s protocol. The reaction was spiked with additional rATP after incubation at 37°C for 30 minutes, and further incubated at 37°C for 2 h. 5 μg of RNA was electroporated into approximately 5.0×106 BHK cells in a 4 mm cuvette using the Bio-Rad Gene Pulser II with the settings adjusted to 850 V, 25 μF. Each cuvette was subjected to 2 pulses at an interval of 3s. The cells were allowed to recover for 10 mins at 37°C and transferred into 15 ml of pre-warmed culture medium in a T75 flask. Cell culture supernatant was collected 5 days after infection and tested for the presence of infectious DENV-1 using plaque assay. The BHK cells were also scraped off and analyzed for the presence of DENV-1 envelope antigen by indirect fluorescent assay (IFA) using the anti-DENV-1 envelope primary mAb HB-47.

### Analysis of sequencing data for DENV-1 isolates

The data analysis pipeline used in this study is built upon open-source tools, which are freely available. The bulk of the sequencing analysis was done using the viral analysis pipeline ViPR (https://github.com/CSB5/vipr), which mainly handles mapping of reads and calling of SNVs with LoFreq [29]. For mapping of Illumina paired-end reads to the Sanger sequenced reference genomes we used BWA version 0.6.2-r126 for all Illumina and version 0.5.9 for all SOLiD sequencing datasets [64]. LoFreq (version 0.6.1) was used for SNV calling using default options and regions overlapping primer positions were ignored. Cold-spot analysis was performed as described in [29]. In brief, SNVs predictions from groups of samples are pooled and then scanned for SNV free regions that are larger than expected (binomial test; Bonferroni corrected p-value < 0.05). Reference genomes used for mapping and annotation were specific for each sample and come from the Sanger consensus sequences reported for viruses in Schreiber et al. 2009 [28].

An in-house R script was developed for calculations and visualization [65]. First, the numbers of reads across the entire sequence were extracted from pileup data files and used as default data values. Additional reads for were extracted from.snp format files for all positions where at least one non-consensus nucleotide was present, over-writing the pileup data for that position. Regions where primers bound to amplify the DENV1 genome, corresponding to positions 1–70, 2065–2084, 4221–4241, 6442–6461, 8519–8540, 10645–10735, were excluded from all analysis. As the typical number of reads is ~100,000, any difference between the proportions of non-consensus bases at different time points or in different hosts that is biologically significant is also statistically significant.

### Statistical analyses

Selection pressure and genetic drift were measured by calculating the dN/dS ratio, which is the ratio of non-synonymous mutation changes per non-synonymous sites (dN) to the synonymous mutation changes per synonymous sites (dS) (S10 File). The Mann-Whitney test was then performed on the dN/dS of the samples between a particular protein coding sequence and compared to the rest of the protein coding sequences within an experimental condition (e.g. the C gene is compared to PrM, E, NS1, NS2a, NS2b, NS3, NS4a, NS4b, 2K and NS5 from the early human samples) (S11 File). The frequency of mutation type was measured by calculating the ratio of transitions (A ↔ G, C ↔ T) to transversions (A↔ C, G ↔ T, G ↔ C, A ↔ T) for each protein coding sequence (S12 File). Diversity and evenness across the viral polyprotein was calculated using the Shannon diversity index and Shannon equitability measurements respectively and the overall number of mutations across the polyprotein (either transitions or transversions) was calculated using a 100bp window (S13 File). We used the Mann-Whitney-Wilcoxon test to assess whether the two samples come from the same population (S14 File).

### Reproducibility

Plasticity at each position-individual-time point was determined by the proportion of the reads that did not agree with the consensus. Three thresholds (0%, 1% and 5%) of plasticity were recorded. For each time point, the number of individuals (out of 12 in human, 11 in mosquitoes) with plasticity above that threshold were counted to assess localized reproducibility across samples. If more than a third of the samples for that position-individual-time point exhibited plasticity (of >0%), this was declared significant: with 99.6% of positions agreeing with the consensus, this threshold leads to a p-value of < 10^−7^. Although this proportion of samples was arbitrarily defined, it nonetheless provides a conservative approach to differentiate stochastic from biologically important variances.

Differences between (logically comparable) pairs of time points were assessed, for each position-individual-pair, by counting differences, differences of at least 0%, 1%, or 5%, between the proportion of reads of the consensus (vs. all others) nucleotide. If more than a third of the samples for that position-individual exhibited a difference between the two time points (of >0%), this was declared significant: with 99.2% of positions not exhibiting differences within the two serum samples, this threshold leads to a p-value of < 10^−5^. Prevalence of differences in different genes were tested via *χ* ^2^ tests between all gene pairs, with Bonferoni’s correction for multiple testing.

### Reversion

For triples of time points (e.g. human early, human late, mosquito late), we quantified pairs of differences for each position-individual combination. In this analysis, we looked for differences of at least 0.1% between the proportions of reads (of the consensus nucleotide at the first time point) for each pair of time points, and coded the sign of the differences. If both differences were positive, this position-individual was coded as changing towards consensus; if negative, as changing away from consensus; and if alternating (positive then negative or vice versa), as a reversion (if either difference were less than 0.1%, the position for that individual was ignored). The number of individuals with each change was recorded as a measure of reproducibility. Reversions with differences of >5% were stored and plotted individually. Consistent reversions across individuals (in more than 25% of samples) were declared significant: with 99.6% of positions in *Ae*. *aegypti*, and 99.5% in *Ae*. *albopictus*, not undergoing reversions, this leads to p<10^−4^. At this evidence threshold, if throughout the entire sequence reversions occurred by chance alone, we would expect to see 0.1 to 0.2 locations declared significant, and there is ~15% chance of seeing 1 false positive, and ~1% chance of seeing 2 or more.

In addition, we identified positions in which the consensus nucleotide itself changed between time points. To do this, we scanned over positions and noted any in which the dominant nucleotide for any two time points, for any individual, differed. Positions in the list for with several such switches were plotted.

## Supporting Information

S1 File Clinical information of the samples used in this study. The strain ID, whether the infection was primary or secondary dengue and the associated clinical manifestations are recorded here.
(EPS)

S2 File Sequencing summary. The host, total reads, reads mapped, percent of reads mapped to DENV1 genome, the sequencing machine used and the average genome coverage are described in this table.
(PDF)

S3 File Comparison of Illumina and Solid Platforms. Ts/Tv ratio was calculated for the polyprotein and for each protein coding sequence. Ts/Tv ratios for Solid and Illumina platforms were compared using the Fisher's exact test.
(PDF)

S4 File Examination of intra-host genetic diversity by position. The DENV genome of each strain was analyzed for positions having detectable, >1% and 5% non-consensus base calls. The number of strains with detectable (grey), >1% (black) and >5% (red) variance are plotted on the y-axis for each position in the DENV genome (x-axis). This analysis was completed for each of the six sample conditions tested: Early Human Serum, Late Human Serum, *Ae*. *aegypti* and *Ae*. *albopictus* inoculated with Early Human Serum, and *Ae*. *aegypti* and *Ae*. *albopictus* inoculated with Late Human Serum. The letters indicate loci with a high degree of reproducibility (more than 1/3 of the samples, corresponding to a *p*-value <10^−5^). The residues at each locus are: 1691 and 1757 (a), 2719 (b), 5164 (c), 6782 (d) and 9986 and 9998 (e).
(EPS)

S5 File Positions of interest. The DENV1 genome positions with high reproducibility across DENV1 strains from the positional examination of variance are notated here. The viral protein and its presumed function are indicated. The amino acid positions are indicated as well as are the codon position of the notable nucleotide and whether the observed variance is expected to change the amino acid encoded.
(EPS)

S6 File LoFreq output. The LoFreq output.snp files for each sample in our data set are in the folder “SNP Files”. The read coverage graphs for each sample are in the folder “Coverage Plots”.
(ZIP)

S7 File Consensus shifts over time by position. Positions evidencing a change in consensus sequence in any of our samples were recorded and interrogated for each DENV-1 strain. Nucleotide sequences are color-coded and consensus is defined as >50% of reads mapping to the indicated position. Each of the circles depicted represents an experimental condition and are in the order of the experimental design figure depicted in Fig 1a.
(PDF)

S8 File Reversion analysis by position. The change in non-consensus base calls was calculated for each position in the DENV genome over the course of infection in Human and then mosquito. The DENV genome of each strain was analyzed and compared for positions undergoing reversion (top panel), moving towards consensus (middle panel) and moving away from consensus (bottom panel) in both *Ae*. *aegypti* (**A**) and *Ae*. *albopictus* (**B**). The number of strains undergoing these events is plotted on the y-axis for each position in the DENV genome (x-axis). The letters indicate loci with a high degree of reproducibility. The residues at each locus in panel (**A**) are: 1757 (a), 5062 (b) and 10253 (c). The residues at each locus in panel (**B**) are: 1757 (a), 2182 (b), 2719 (c), 3247 (d), 5062 (e), 9071 (g) and 10253 (h).
(EPS)

S9 File Positions undergoing large reversions. Positions within the DENV genome that undergo a change greater than five percent during the transition through human into *Ae*. *aegypti* mosquito are recorded here. Position number and the base changes are indicated. Percentage change over the course of the human infection is indicated by the left arrow in each box and the percentage change during the *Ae*. *aegypti* infection is indicated by the arrow on the right.
(EPS)

S10 File dN/dS analysis. The ratio of the mean nonsynonymous and synonymous mutations were calculated for the polyprotein as well as for each protein coding sequence.
(PDF)

S11 File Mann-Whitney test for significant dN/dS differences between protein coding sequences. The Mann-Whitney test was used to compare individual dN/dS values for each protein coding sequence to the other protein coding sequences within each experimental condition.
(PDF)

S12 File Transition/Transversion ratio. Transition (Ts) is the mutation between the same nucleotide type (A ↔ G, C ↔ T) and transversion (Tv) is mutation that results in change in nucleotide type (A↔ C, G ↔ T, G ↔ C, A ↔ T). The ratio of these mutation types is calculated here.
(PDF)

S13 File Mutation rate, diversity and evenness across the polyprotein. The number of mutations in a 100bp window were calculated across the genome. Diversity and evenness of the nucleotide type change (A ↔ G, C ↔ T, A↔ C, G ↔ T, G ↔ C, A ↔ T) were calculated using the Shannon diversity index and Shannon equitability measurement respectively.
(PDF)

S14 File Mann-Whitney-Wilcoxon test. The dN/dS and Ts/Tv ratios for the polyprotein across experimental conditions were compared using the non-parametric Mann-Whitney-Wilcoxon test. Significant results are depicted in bold.
(PDF)

S15 File Structure prediction of the 3’UTR. The folding structures for the single consensus change observed in the 3’UTR (position 10541) were predicted with mFold. The predominant base at this position (U) is depicted in panel A and the variant (C) is depicted in panel B. Arrows indicate this position in each panel.
(EPS)

S16 File Primers used in this study. The primers used to amplify the DENV1 genome are recorded here.
(EPS)
