# Supplementary material for: Analysis of Dengue Virus Genetic Diversity during Human and Mosquito Infection Reveals Genetic Constraints
Source: PLoS Negl Trop Dis. 2015 Sep 1;9(9):e0004044. doi: 10.1371/journal.pntd.0004044 (PMC4556638; doi:10.1371/journal.pntd.0004044)
Supplement: S2 File — The host, total reads, reads mapped, percent of reads mapped to DENV1 genome, the sequencing machine used and the average genome coverage are described in this table. (PDF) [file pntd.0004044.s002.pdf]

S2 File

Sequencing statistics

No	Sample	Host	Total reads	Reads Mapped	% mapped	Sequencer	Average genome coverage
1	05K2913DK1	Early Aegypti	30430386	30226229	99.3	Illumina	210748.0
2	05K2913DK1	Early Albopictus	26696038	26557629	99.5	Illumina	187660.0
3	05K2913DK1	Early Human	14786942	11385330	77.0	Illumina	79730.1
4	05K2913DK2	Late Aegypti	14628880	14500160	99.1	Illumina	102453.0
5	05K2913DK2	Late Albopictus	14295914	14214056	99.4	Illumina	100432.0
6	05K2913DK2	Late Human	14239082	14023029	98.5	Illumina	99035.2
7	05K3911DK1	Early Aegypti	14279724	14077044	98.6	Illumina	99271.3
8	05K3911DK1	Early Albopictus	11420558	11349381	99.4	Illumina	80160.4
9	05K3911DK1	Early Human	12441192	12272658	98.6	Illumina	86661.3
10	05K3911DK2	Late Aegypti	14604892	14512627	99.4	Illumina	102491.0
11	05K3911DK2	Late Albopictus	21394862	21286143	99.5	Illumina	150327.0
12	05K3911DK2	Late Human	14671546	14522898	99.0	Illumina	102580.0
13	05K4138DK1	Early Aegypti	31171530	30724064	98.6	Illumina	208174.0
14	05K4138DK1	Early Albopictus	16758412	16576321	98.9	Illumina	117100.0
15	05K4138DK1	Early Human	16096964	15955929	99.1	Illumina	112737.0
16	05K4138DK2	Late Aegypti	15372060	13759857	89.5	Illumina	96874.5
17	05K4138DK2	Late Albopictus	15218598	14511515	95.4	Illumina	102505.0
18	05K4138DK2	Late Human	15257344	15075385	98.8	Illumina	106489.0
19	05K4139DK1	Early Aegypti	17038996	16851832	98.9	Illumina	119037.0
20	05K4139DK1	Early Albopictus	14990656	14713173	98.1	Illumina	103109.0
21	05K4139DK1	Early Human	15150116	14605107	96.4	Illumina	103077.0
22	05K4139DK2	Late Aegypti	15870944	15600100	98.3	Illumina	110152.0
23	05K4139DK2	Late Albopictus	30656810	30308223	98.9	Illumina	213647.0
24	05K4139DK2	Late Human	16691472	16463659	98.6	Illumina	116267.0
25	05K4152DK1	Early Aegypti	27357370	26602307	97.2	Illumina	187024.0
26	05K4152DK1	Early Albopictus	14672628	14484696	98.7	Illumina	102305.0
27	05K4152DK1	Early Human	15009638	11895575	79.3	Illumina	83933.4
28	05K4152DK2	Late Aegypti	15667204	15216413	97.1	Illumina	107495.0
29	05K4152DK2	Late Albopictus	14662688	14217207	97.0	Illumina	99326.4
30	05K4152DK2	Late Human	7979528	7746036	97.1	Illumina	54655.2
31	05K4172DK1	Early Aegypti	15833606	15767464	99.6	Illumina	111384.0
32	05K4172DK1	Early Albopictus	12641182	12574841	99.5	Illumina	88761.5
33	05K4172DK1	Early Human	2800370	1150758	41.1	Solid	5145.3
34	05K4172DK2	Late Aegypti	14042070	13960771	99.4	Illumina	98620.7
35	05K4172DK2	Late Albopictus	15910590	15759133	99.0	Illumina	111104.0
36	05K4172DK2	Late Human	2692795	1286527	47.8	Solid	5752.4
37	05K4173DK1	Early Aegypti	13025478	12925073	99.2	Illumina	91275.7
38	05K4173DK1	Early Albopictus	19547934	19412971	99.3	Illumina	137043.0
39	05K4173DK1	Early Human	6648857	2478059	37.3	Solid	11080.1
40	05K4173DK2	Late Aegypti	18874170	18731741	99.2	Illumina	132170.0
41	05K4173DK2	Late Albopictus	31742446	31467250	99.1	Illumina	219136.0
42	05K4173DK2	Late Human	3292025	1337971	40.6	Solid	5982.4
43	05K4441DK1	Early Aegypti	37700276	37334253	99.0	Illumina	257408.0
44	05K4441DK1	Early Albopictus	21606624	21113333	97.7	Illumina	149007.0
45	05K4441DK1	Early Human	32364600	32068535	99.1	Illumina	222252.0
46	05K4441DK2	Late Aegypti	22866908	22655764	99.1	Illumina	159861.0
47	05K4441DK2	Late Albopictus	22890952	22665845	99.0	Illumina	159962.0
48	05K4441DK2	Late Human	33950636	33479193	98.6	Illumina	229875.0
49	05K4468DK1	Early Aegypti	29580792	29195211	98.7	Illumina	205122.0

50	05K4468DK1	Early Albopictus	19968700	19693786	98.6	Illumina	136187.0
51	05K4468DK1	Early Human	18979296	18759330	98.8	Illumina	132304.0
52	05K4468DK2	Late Aegypti	17992344	17846436	99.2	Illumina	125659.0
53	05K4468DK2	Late Albopictus	15450152	15288630	99.0	Illumina	107778.0
54	05K4468DK2	Late Human	16986098	16771338	98.7	Illumina	118460.0
55	05K4620DK1	Early Human	3650110	1242491	34.0	Solid	5555.5
56	05K4620DK2	Late Human	7035992	1681789	23.9	Solid	7519.7
57	05K4621DK1	Early Aegypti	17925116	17851111	99.6	Illumina	126017.0
58	05K4621DK1	Early Albopictus	27668176	27435855	99.2	Illumina	192939.0
59	05K4621DK1	Early Human	4571323	1683306	36.8	Solid	7526.5
60	05K4621DK2	Late Aegypti	17964348	17856193	99.4	Illumina	126170.0
61	05K4621DK2	Late Albopictus	15944506	15838849	99.3	Illumina	111875.0
62	05K4621DK2	Late Human	3788053	1509036	39.8	Solid	6747.3
63	05K4622DK1	Early Aegypti	19699700	19616813	99.6	Illumina	138559.0
64	05K4622DK1	Early Albopictus	14575292	14417610	98.9	Illumina	101716.0
65	05K4622DK1	Early Human	18418402	18125182	98.4	Illumina	127863.0
66	05K4622DK2	Late Aegypti	17546384	17415970	99.3	Illumina	123026.0
67	05K4622DK2	Late Albopictus	20905624	20821735	99.6	Illumina	147133.0
68	05K4622DK2	Late Human	18765090	16882977	90.0	Illumina	119126.0

S2 File. Sequencing summary. The host, total reads, reads mapped, percent of reads mapped to DENV1 genome, the sequencing machine used and the average genome coverage are described in this table
