# Supplementary material for: Analysis of Dengue Virus Genetic Diversity during Human and Mosquito Infection Reveals Genetic Constraints
Source: PLoS Negl Trop Dis. 2015 Sep 1;9(9):e0004044. doi: 10.1371/journal.pntd.0004044 (PMC4556638; doi:10.1371/journal.pntd.0004044)
Supplement: S3 File — Ts/Tv ratio was calculated for the polyprotein and for each protein coding sequence. Ts/Tv ratios for Solid and Illumina platforms were compared using the Fisher's exact test. (PDF) [file pntd.0004044.s003.pdf]

S3 File

Solid vs Illumina Comparison

	Polyprotein	C	prM	E	NS1	NS2A	NS2B	NS3	NS4A	2K protein	NS4B	NS5
**Ts/Tv ratio – Solid**	4.429	No Tv	4	4.5	7	1.125	2.5	No Tv	3	No Tv	12	7.5
**Ts/Tv ratio – Illumina**	4.013	4.2	8.6	3.467	4.579	3.35	3.75	3.476	8.333	2.25	3.273	4.438
**Fisher's Test**	0.801	1	0.465	0.786	0.736	0.069	1	0.198	0.431	1	0.433	0.742

S3 File. Comparison of Illumina and Solid Platforms. Ts/Tv ratio was calculated for the polyprotein and for each protein coding sequence. Ts/Tv ratios for Solid and Illumina platforms were compared using the Fisher's exact test.
