# Supplementary figures and images for: Analysis of Dengue Virus Genetic Diversity during Human and Mosquito Infection Reveals Genetic Constraints
Source: PLoS Negl Trop Dis. 2015 Sep 1;9(9):e0004044. doi: 10.1371/journal.pntd.0004044 (PMC4556638; doi:10.1371/journal.pntd.0004044)

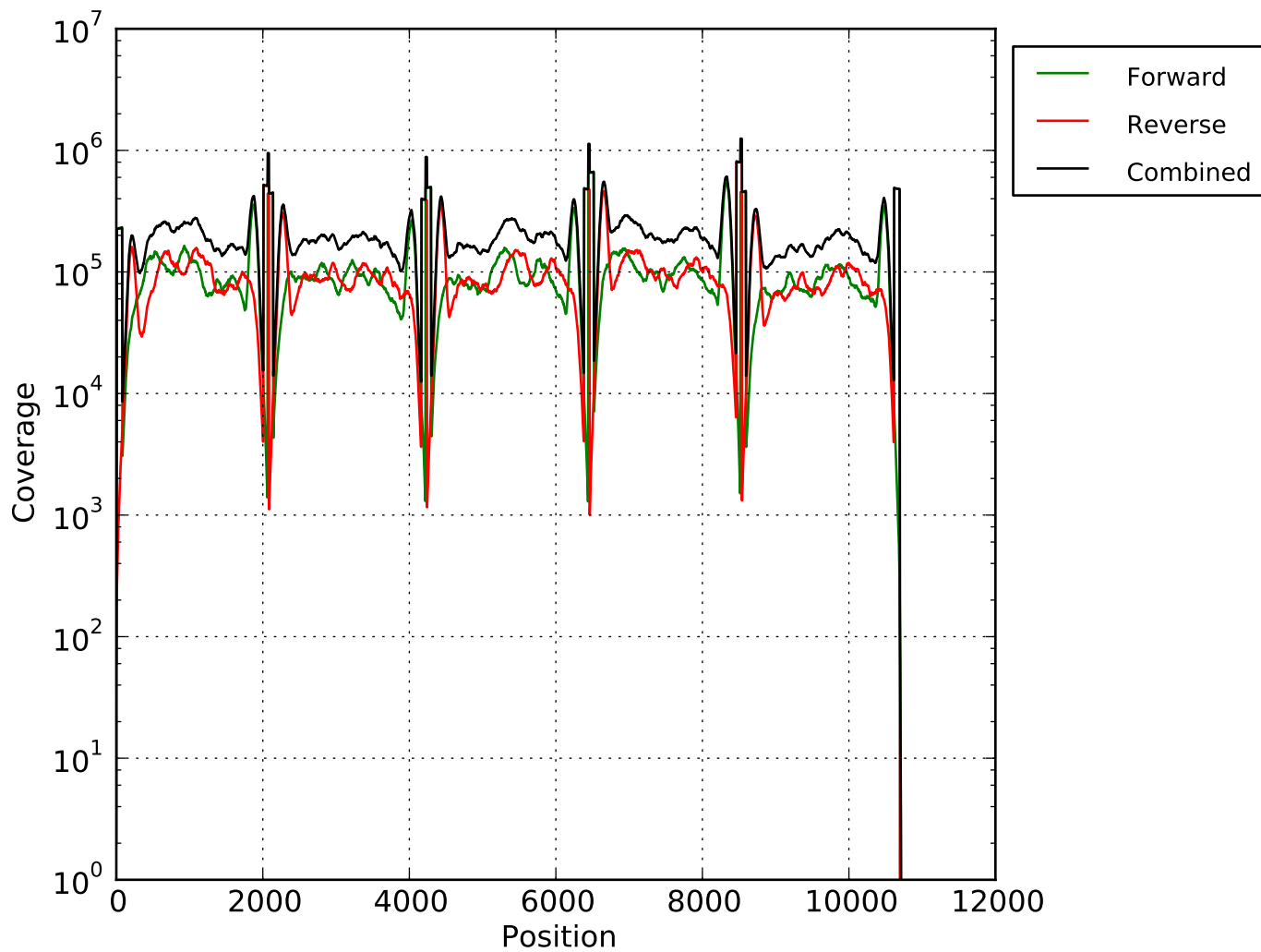

Supplement: S6 File — The LoFreq output.snp files for each sample in our data set are in the folder “SNP Files”. The read coverage graphs for each sample are in the folder “Coverage Plots”. (ZIP) [file pntd.0004044.s006.zip › S6_File/Coverage Plots/05K2913DK1-Aeg-Coverage.pdf]

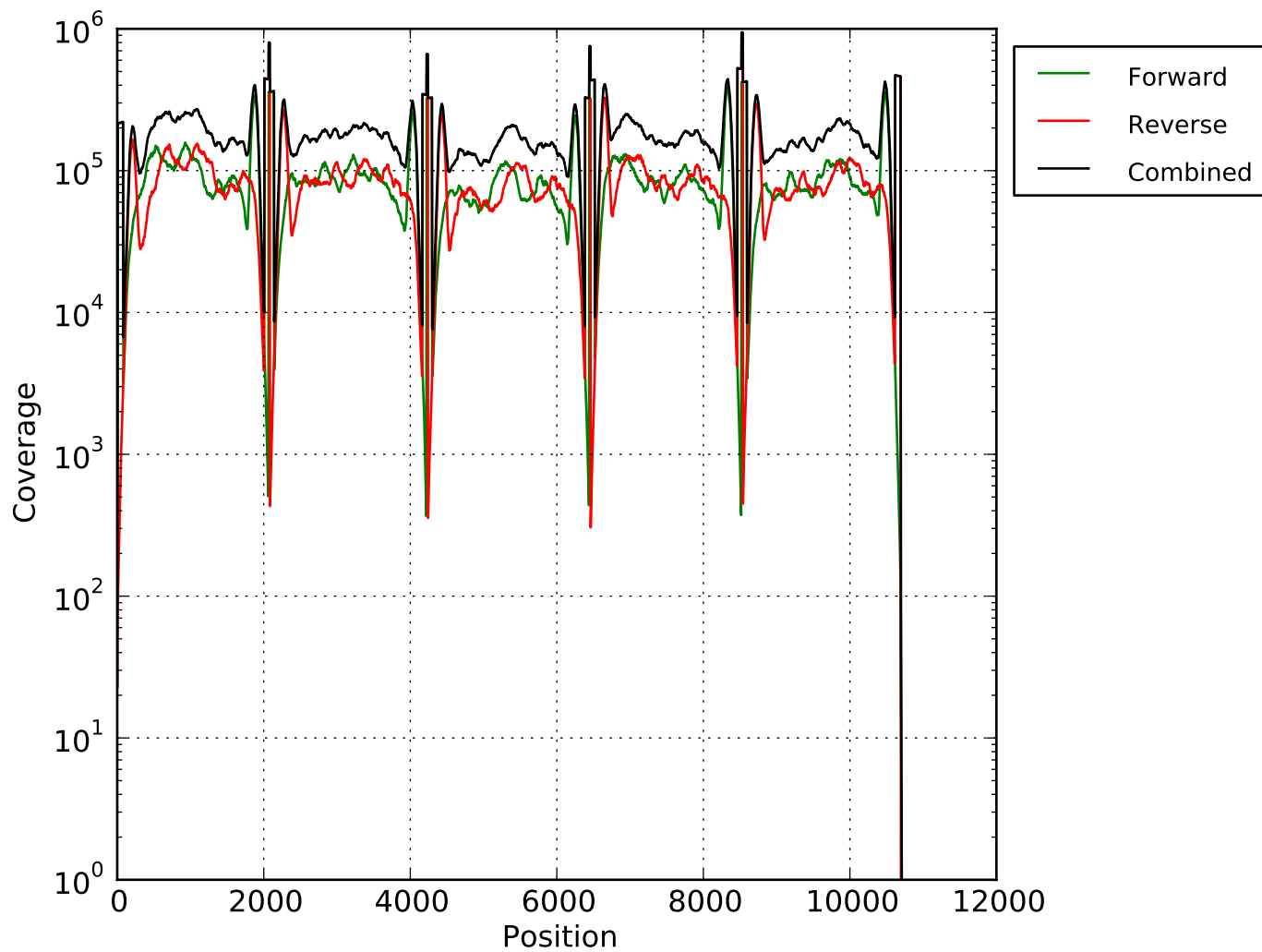

Supplement: S6 File — The LoFreq output.snp files for each sample in our data set are in the folder “SNP Files”. The read coverage graphs for each sample are in the folder “Coverage Plots”. (ZIP) [file pntd.0004044.s006.zip › S6_File/Coverage Plots/05K2913DK1-Albo-Coverage.pdf]

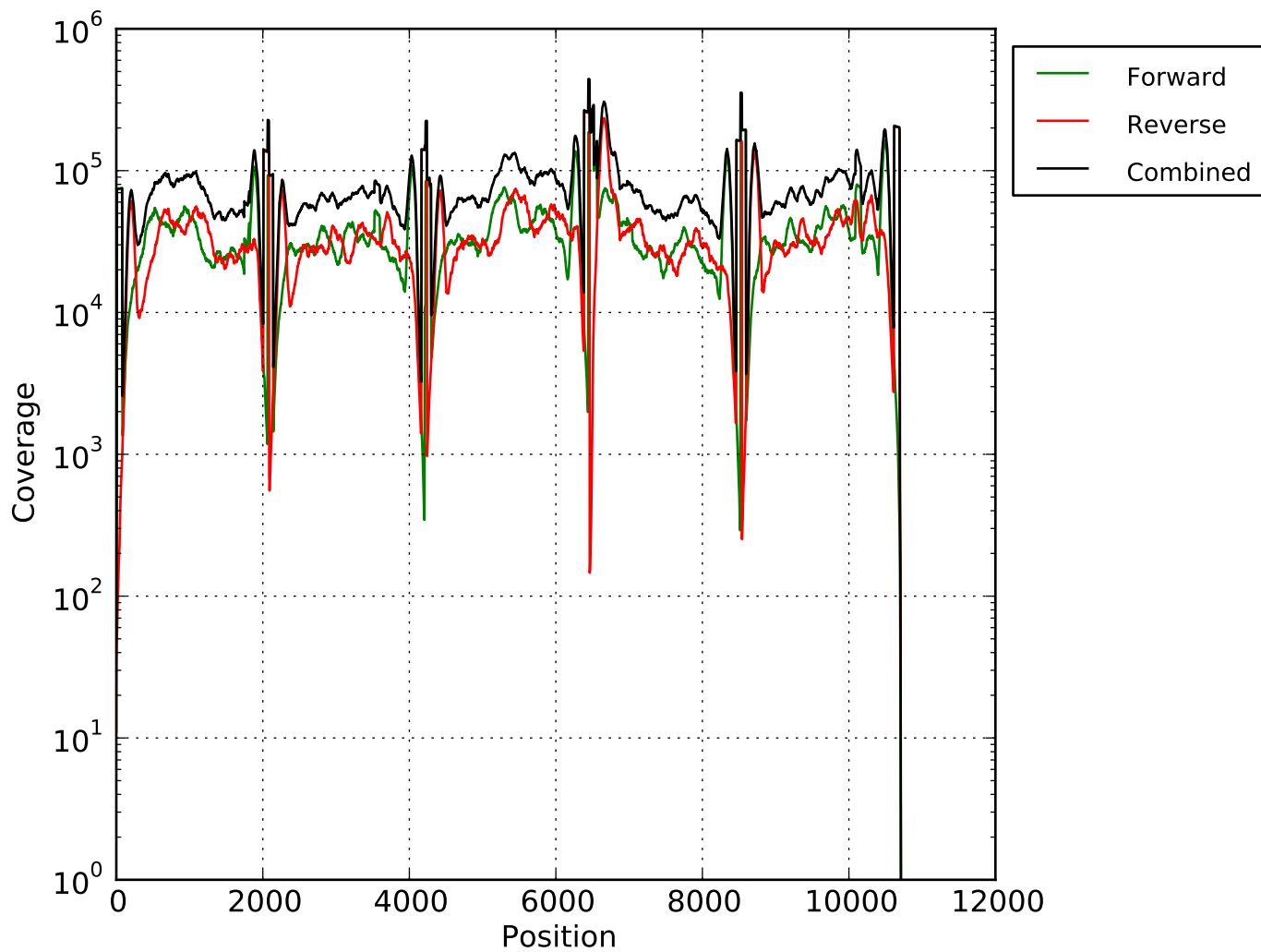

Supplement: S6 File — The LoFreq output.snp files for each sample in our data set are in the folder “SNP Files”. The read coverage graphs for each sample are in the folder “Coverage Plots”. (ZIP) [file pntd.0004044.s006.zip › S6_File/Coverage Plots/05K2913DK1-Serum-Coverage.pdf]

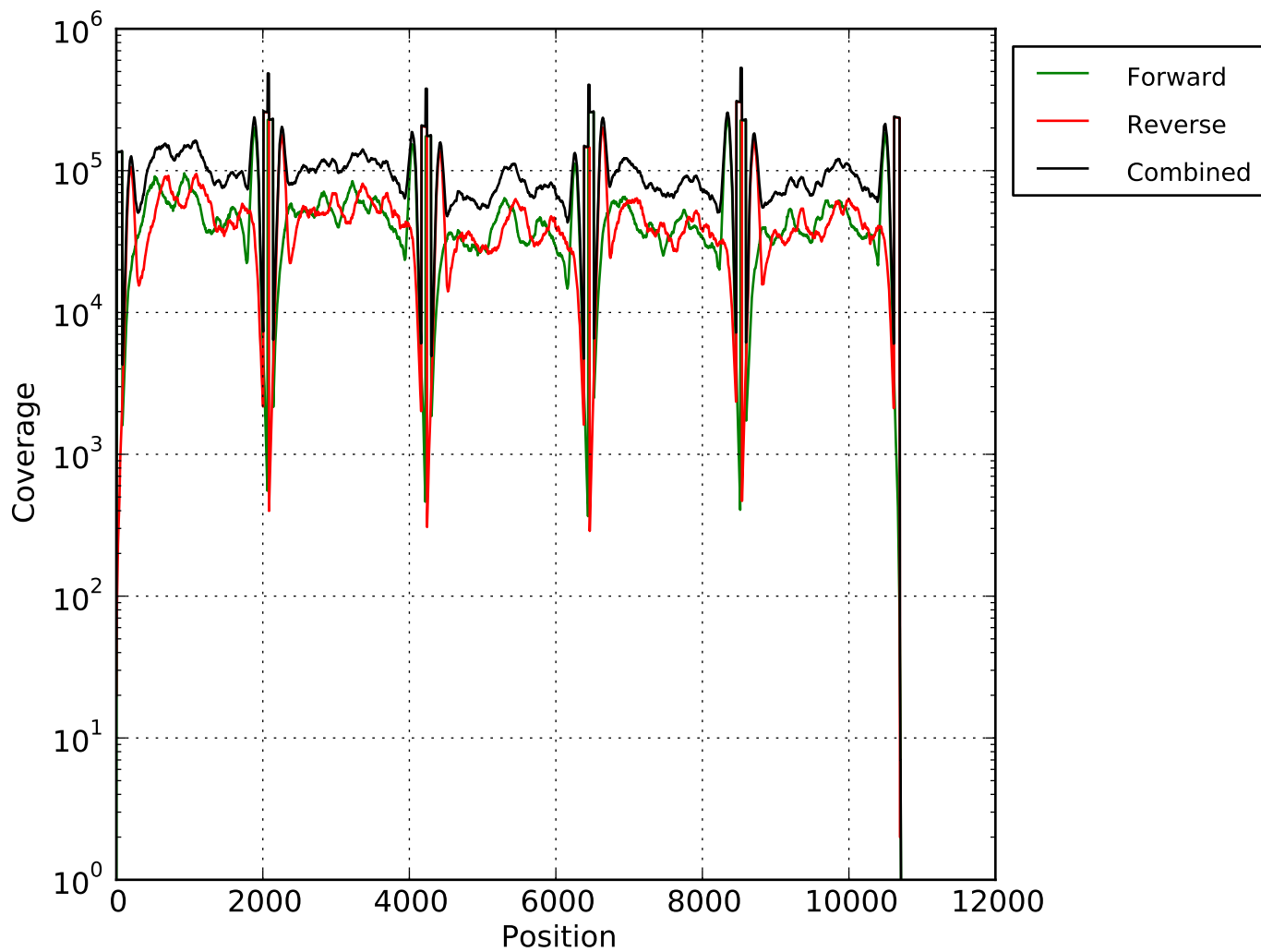

Supplement: S6 File — The LoFreq output.snp files for each sample in our data set are in the folder “SNP Files”. The read coverage graphs for each sample are in the folder “Coverage Plots”. (ZIP) [file pntd.0004044.s006.zip › S6_File/Coverage Plots/05K2913DK2-Aeg-Coverage.pdf]

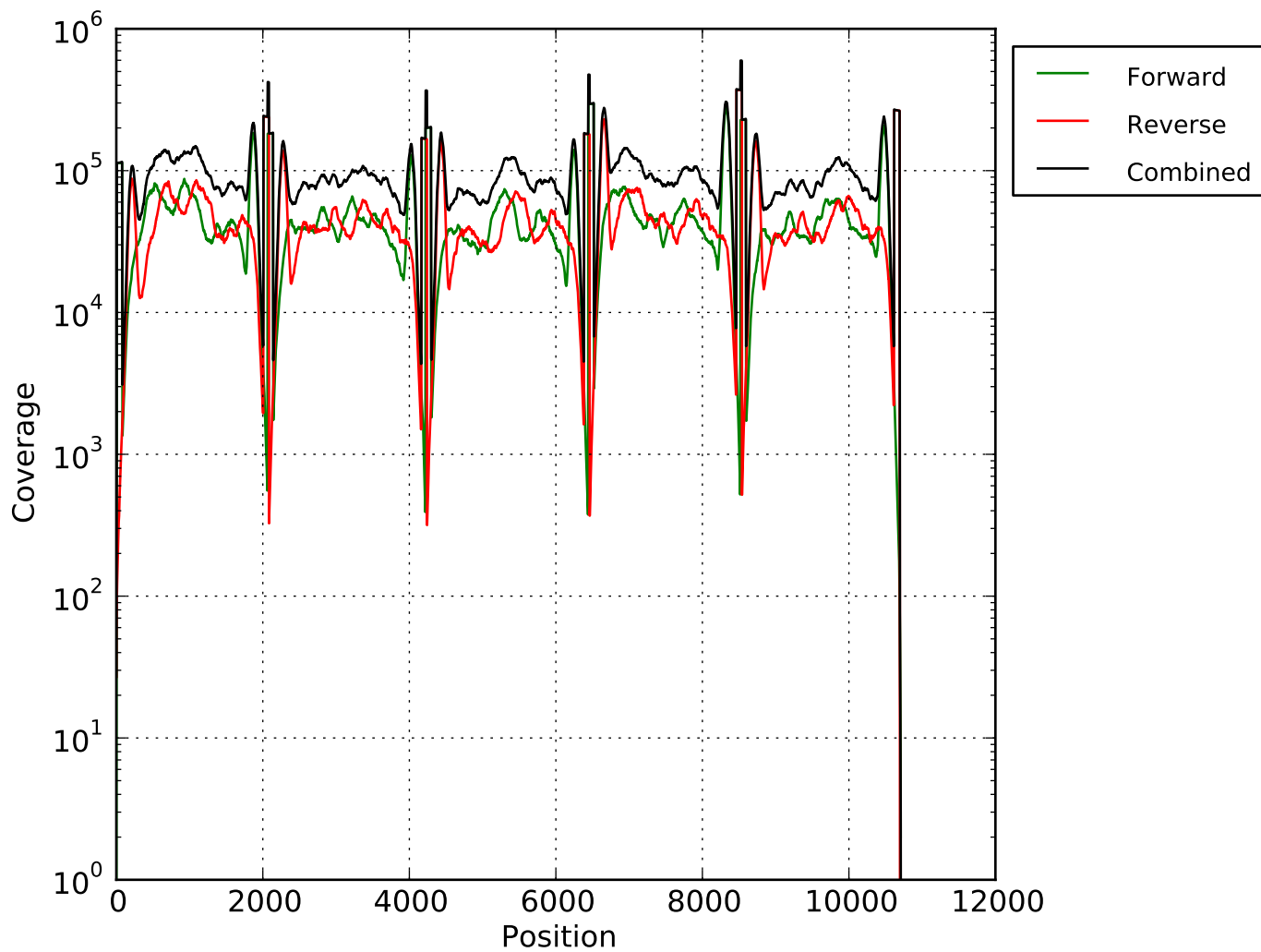

Supplement: S6 File — The LoFreq output.snp files for each sample in our data set are in the folder “SNP Files”. The read coverage graphs for each sample are in the folder “Coverage Plots”. (ZIP) [file pntd.0004044.s006.zip › S6_File/Coverage Plots/05K2913DK2-Albo-Coverage.pdf]

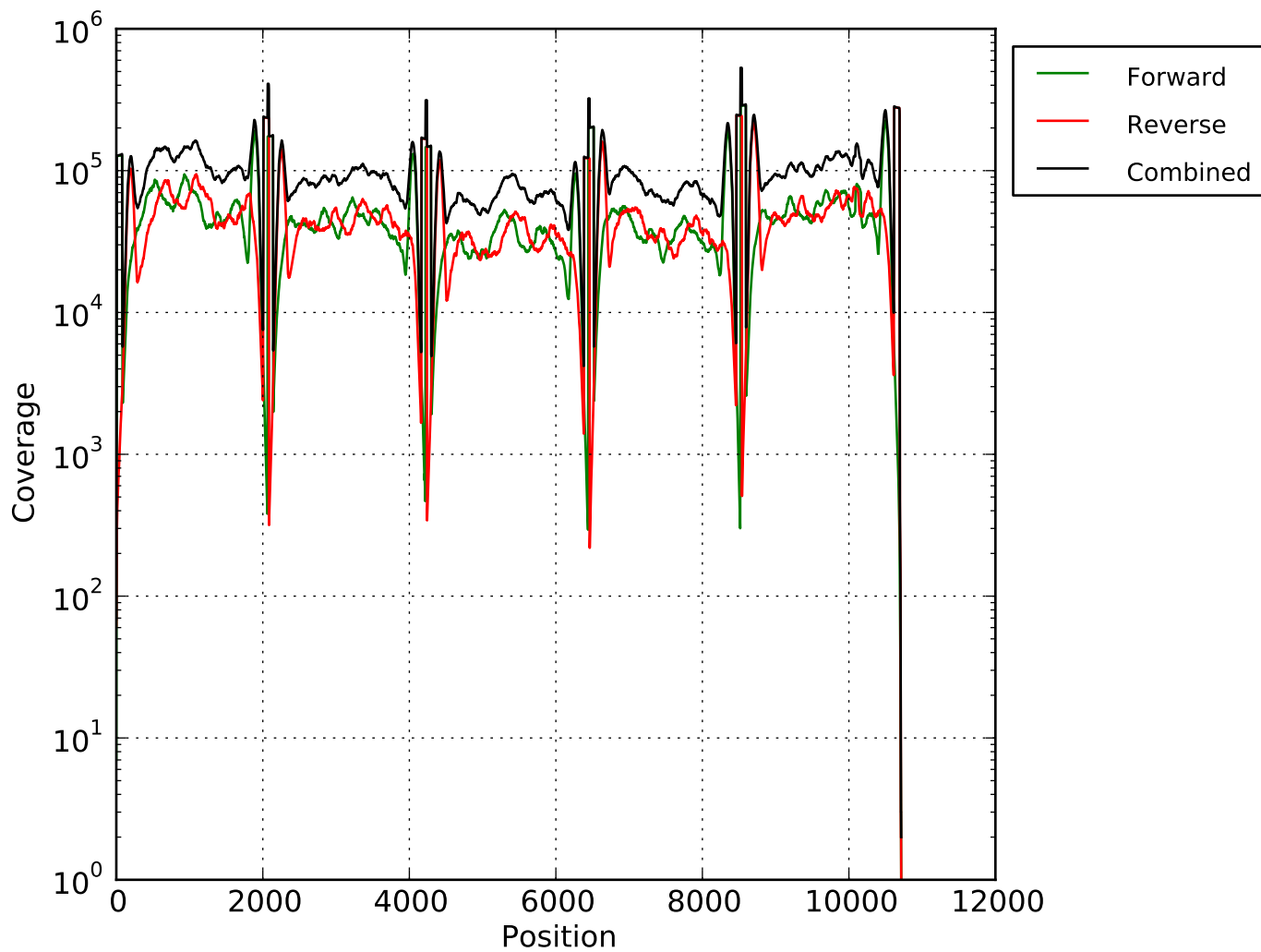

Supplement: S6 File — The LoFreq output.snp files for each sample in our data set are in the folder “SNP Files”. The read coverage graphs for each sample are in the folder “Coverage Plots”. (ZIP) [file pntd.0004044.s006.zip › S6_File/Coverage Plots/05K2913DK2-Serum-Coverage.pdf]

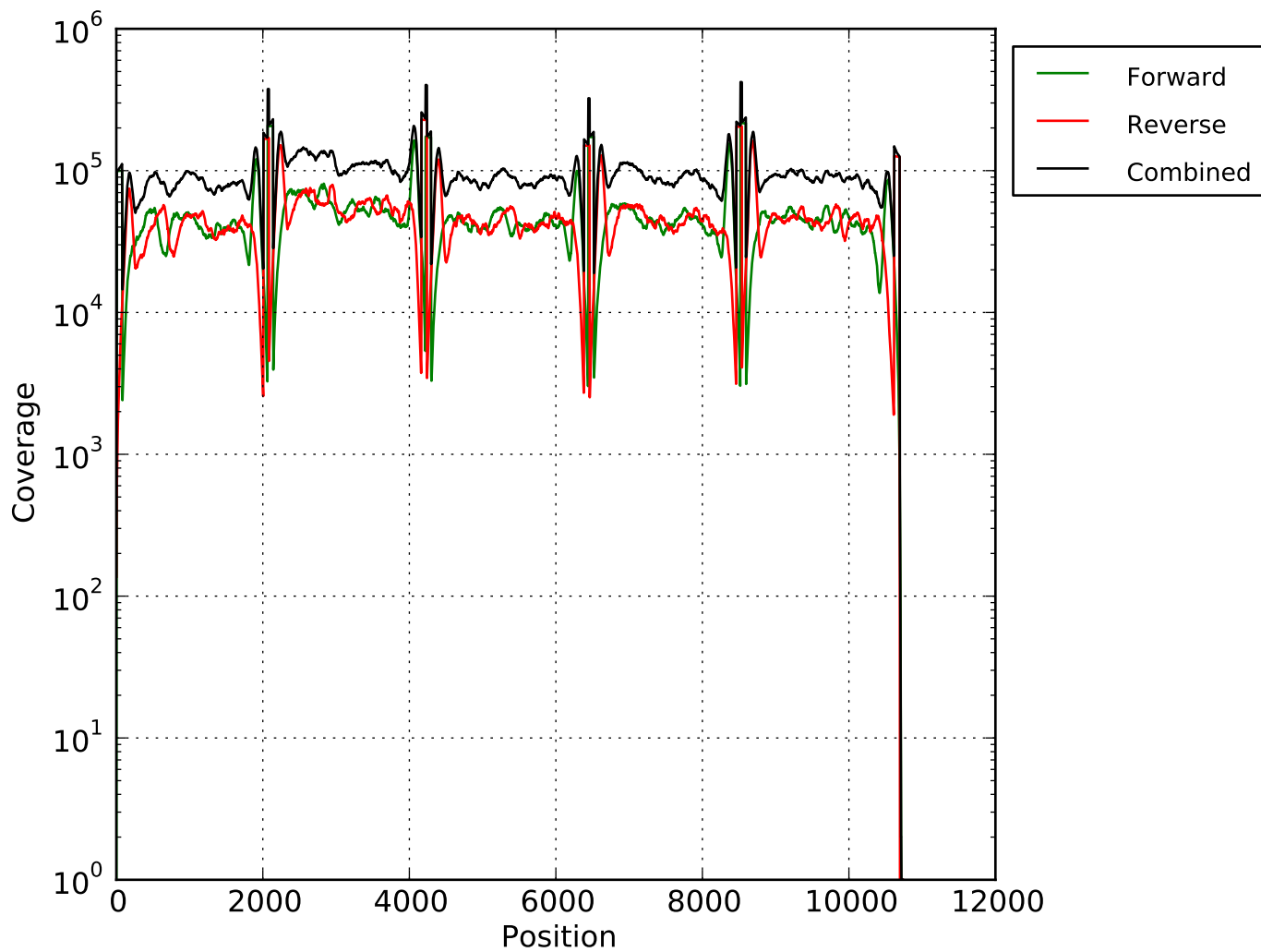

Supplement: S6 File — The LoFreq output.snp files for each sample in our data set are in the folder “SNP Files”. The read coverage graphs for each sample are in the folder “Coverage Plots”. (ZIP) [file pntd.0004044.s006.zip › S6_File/Coverage Plots/05K3911DK1-Aeg-Coverage.pdf]

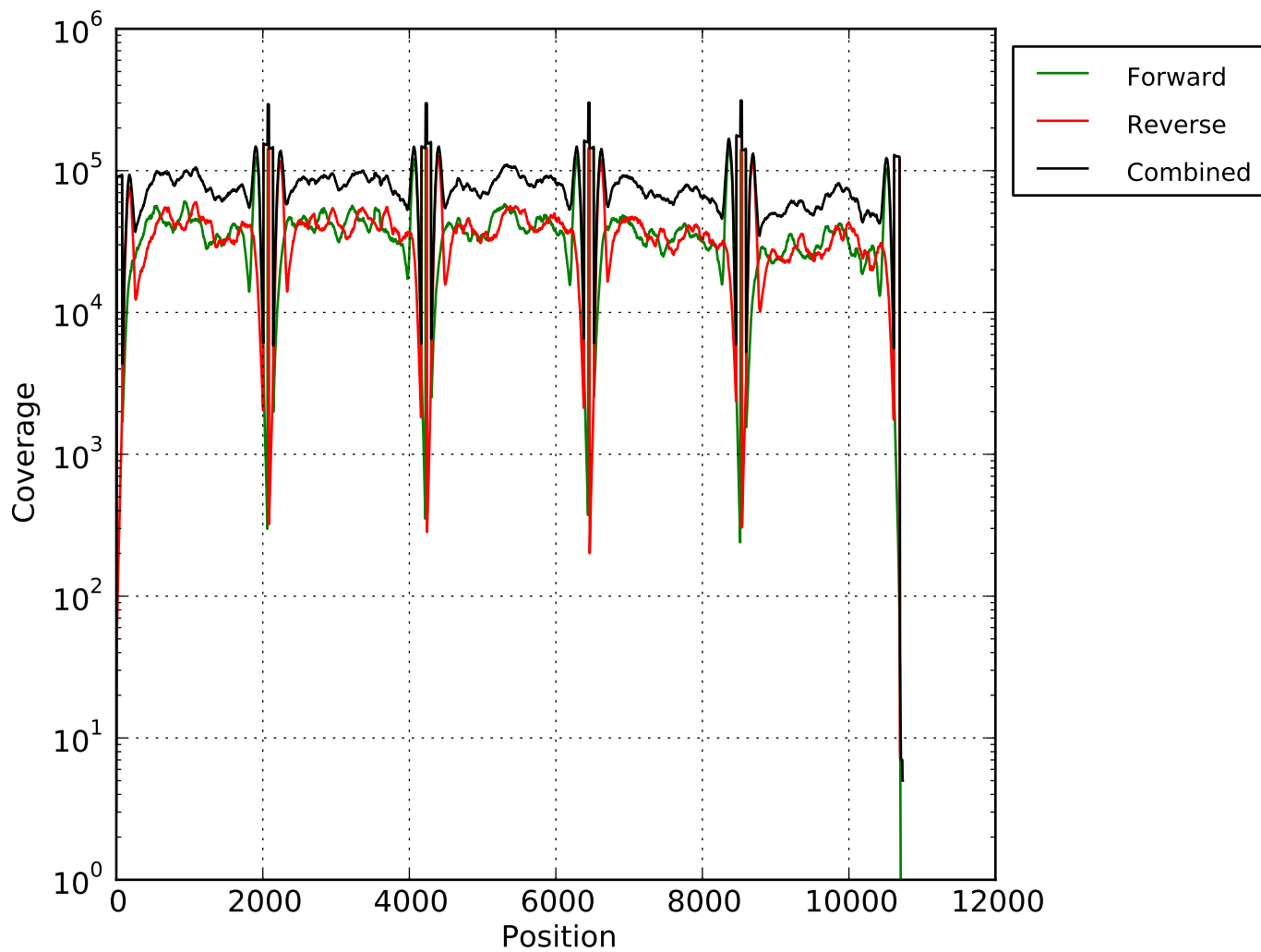

Supplement: S6 File — The LoFreq output.snp files for each sample in our data set are in the folder “SNP Files”. The read coverage graphs for each sample are in the folder “Coverage Plots”. (ZIP) [file pntd.0004044.s006.zip › S6_File/Coverage Plots/05K3911DK1-Albo-Coverage.pdf]

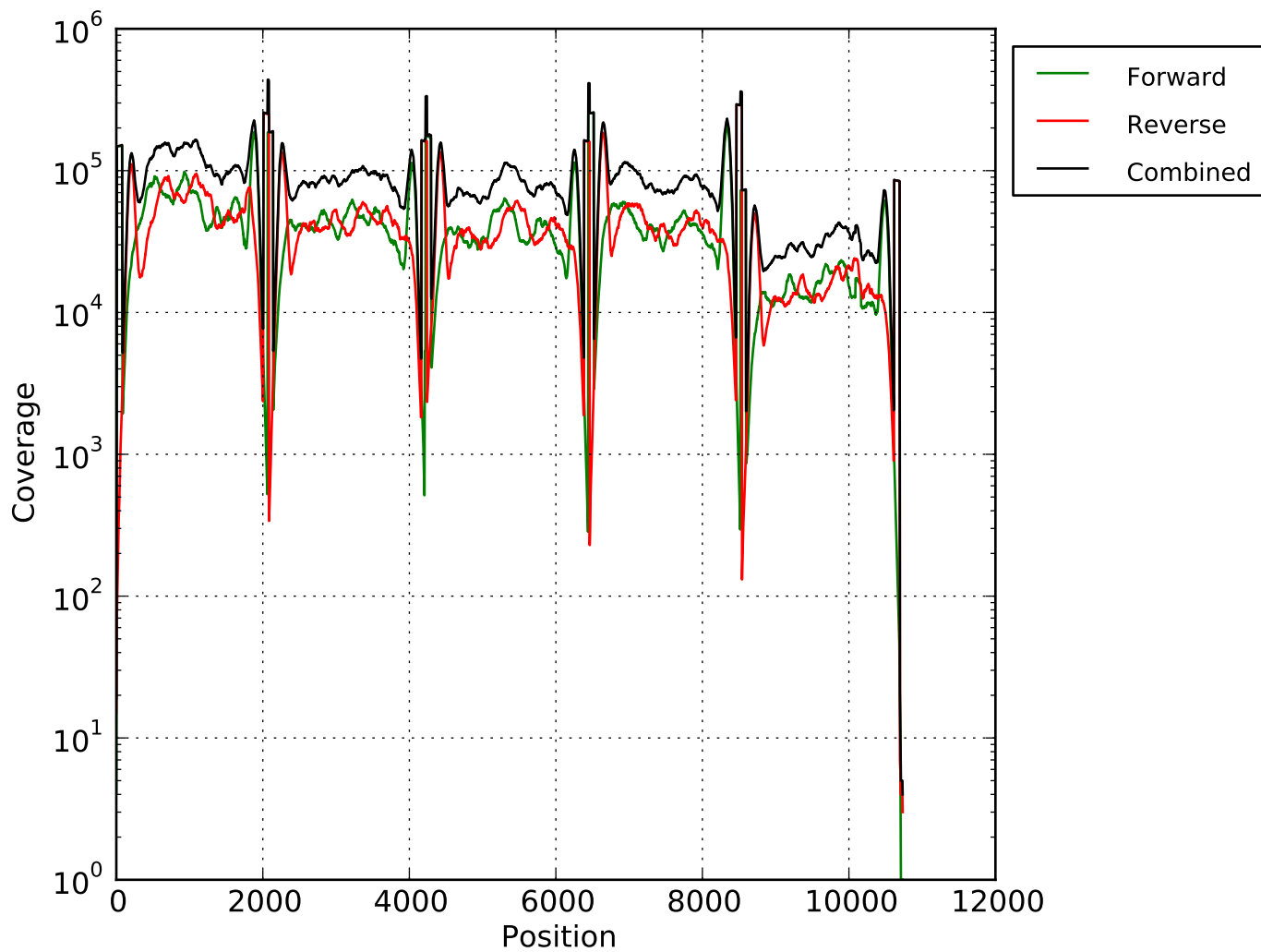

Supplement: S6 File — The LoFreq output.snp files for each sample in our data set are in the folder “SNP Files”. The read coverage graphs for each sample are in the folder “Coverage Plots”. (ZIP) [file pntd.0004044.s006.zip › S6_File/Coverage Plots/05K3911DK1-Serum-Coverage.pdf]

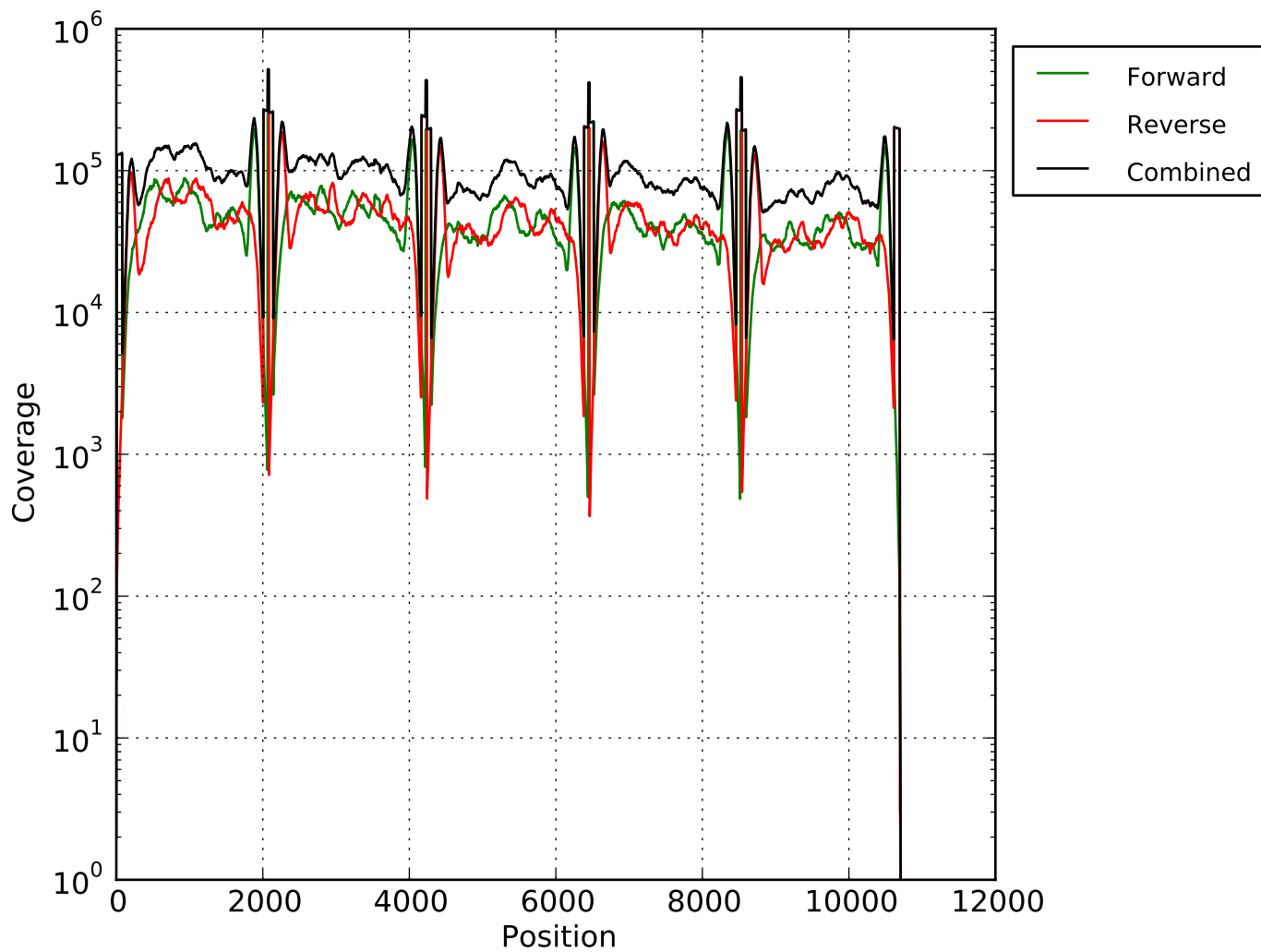

Supplement: S6 File — The LoFreq output.snp files for each sample in our data set are in the folder “SNP Files”. The read coverage graphs for each sample are in the folder “Coverage Plots”. (ZIP) [file pntd.0004044.s006.zip › S6_File/Coverage Plots/05K3911DK2-Aeg-Coverage.pdf]

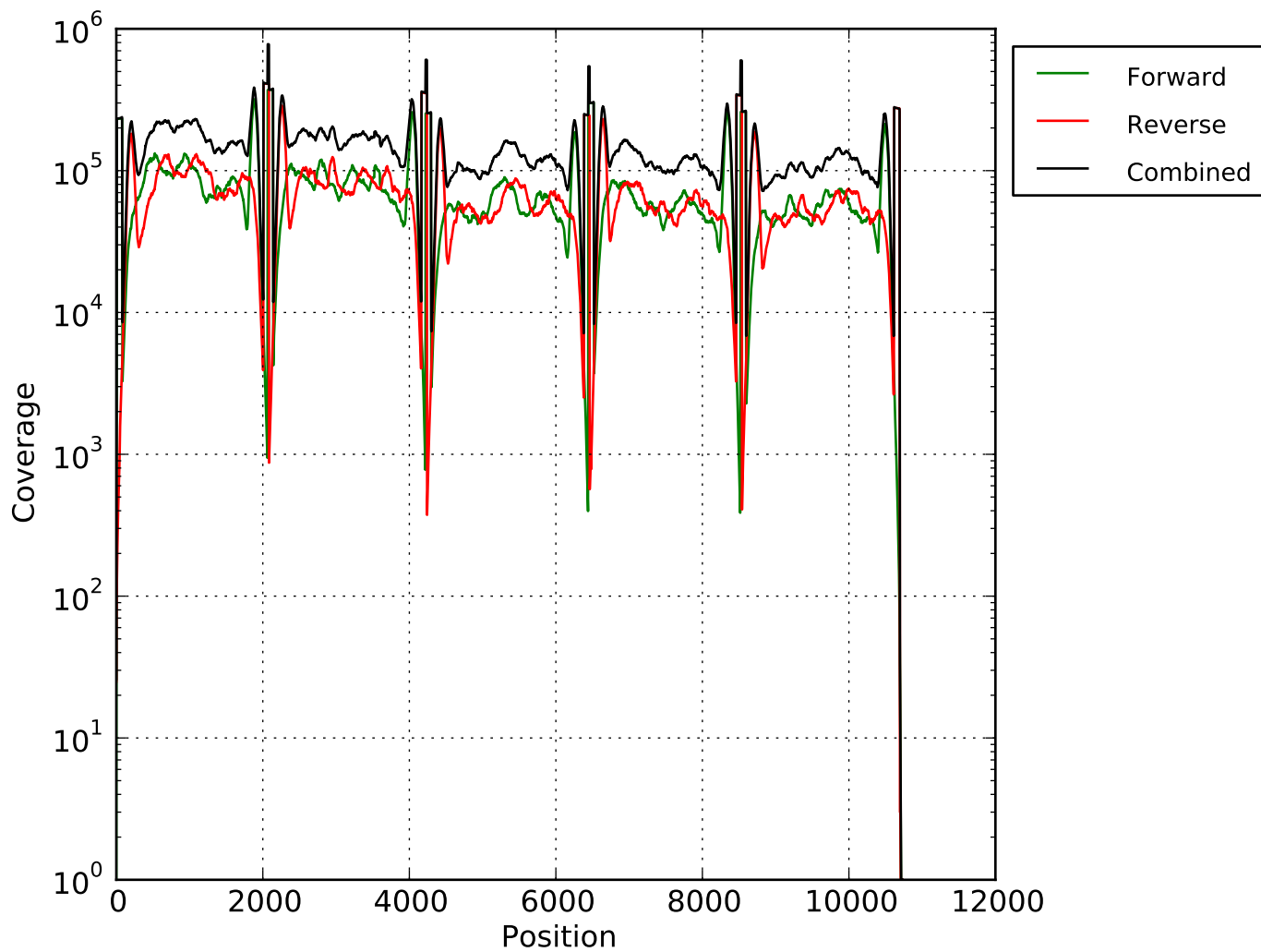

Supplement: S6 File — The LoFreq output.snp files for each sample in our data set are in the folder “SNP Files”. The read coverage graphs for each sample are in the folder “Coverage Plots”. (ZIP) [file pntd.0004044.s006.zip › S6_File/Coverage Plots/05K3911DK2-Albo-Coverage.pdf]

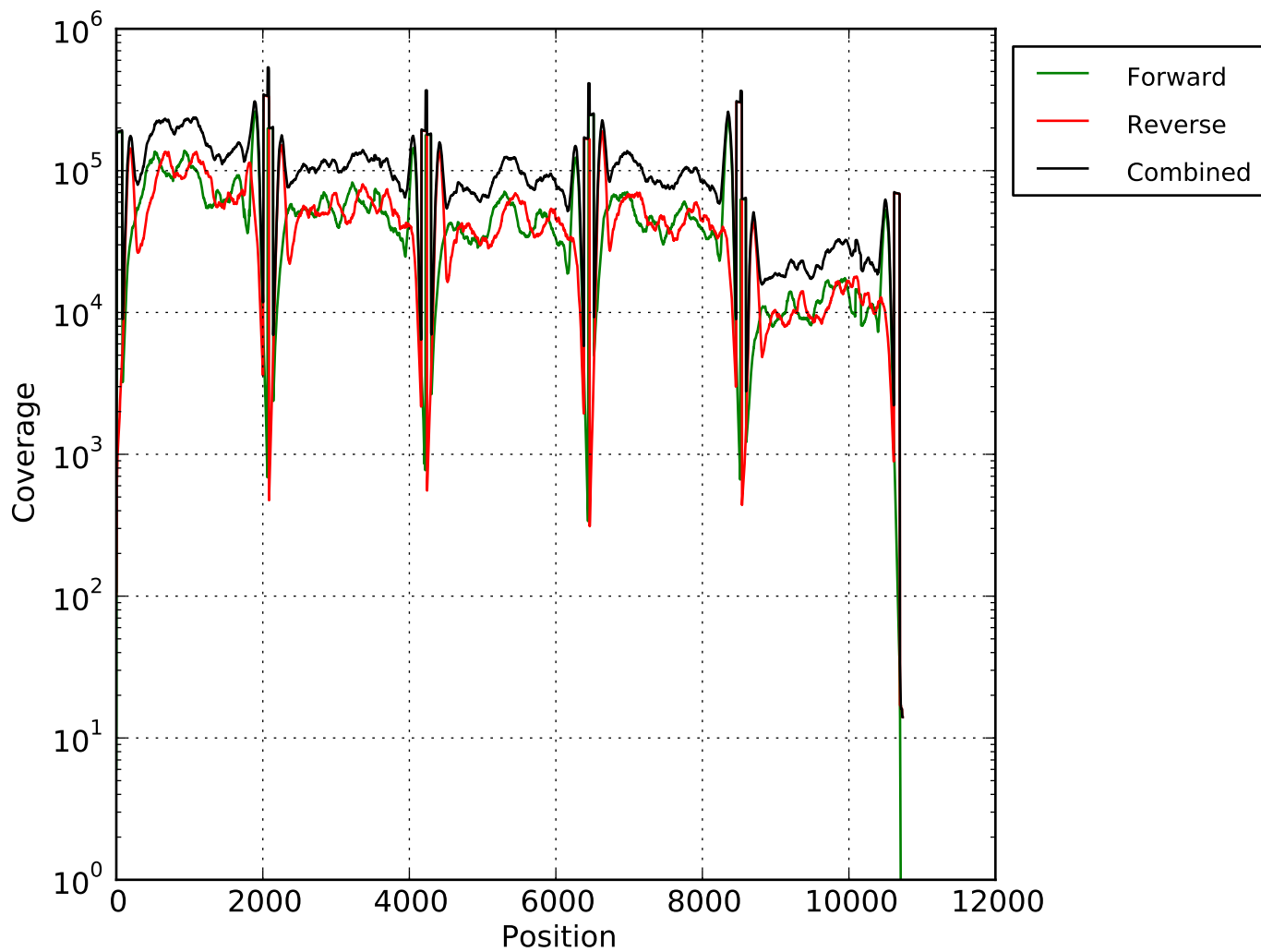

Supplement: S6 File — The LoFreq output.snp files for each sample in our data set are in the folder “SNP Files”. The read coverage graphs for each sample are in the folder “Coverage Plots”. (ZIP) [file pntd.0004044.s006.zip › S6_File/Coverage Plots/05K3911DK2-Serum-Coverage.pdf]

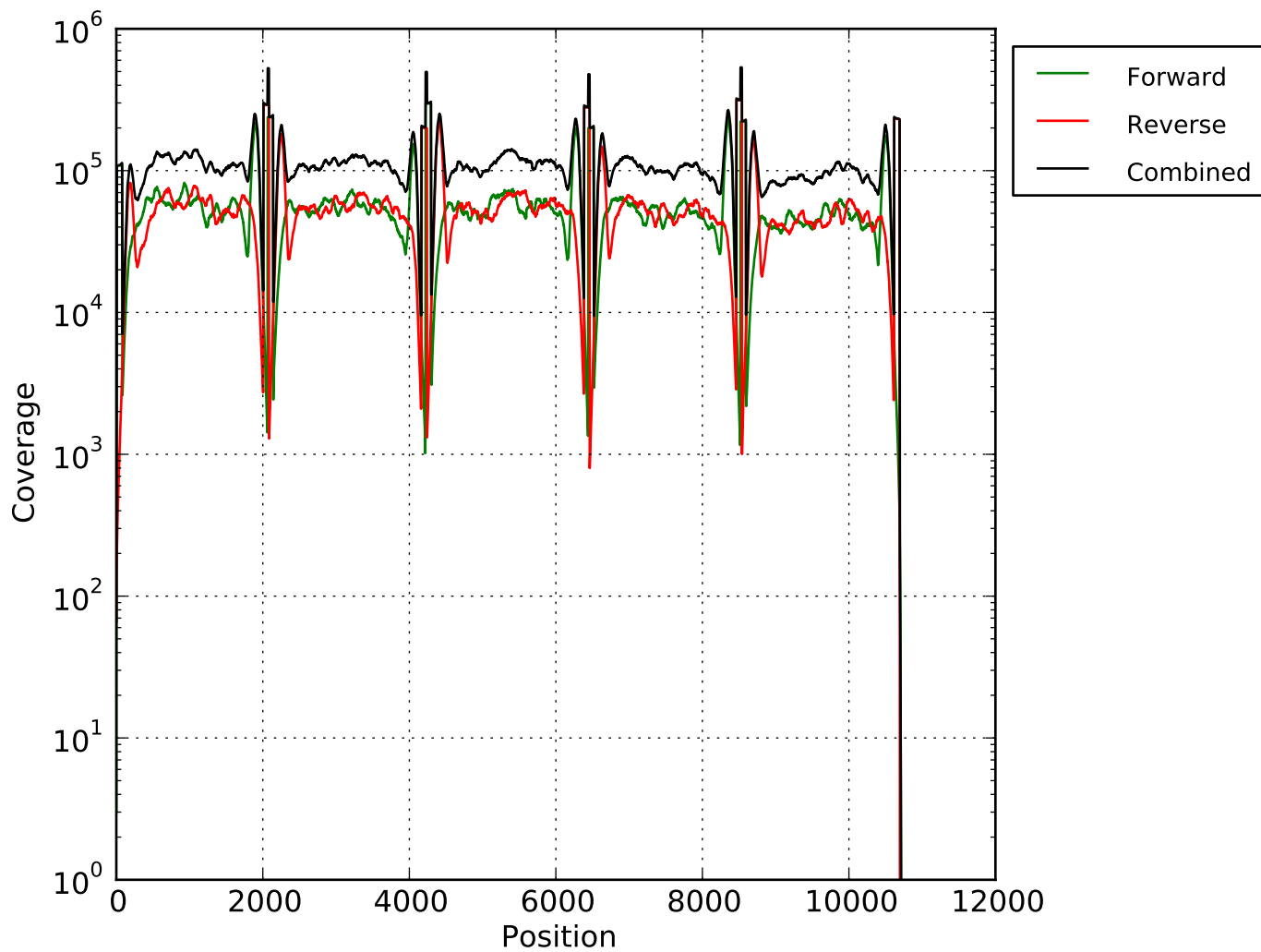

Supplement: S6 File — The LoFreq output.snp files for each sample in our data set are in the folder “SNP Files”. The read coverage graphs for each sample are in the folder “Coverage Plots”. (ZIP) [file pntd.0004044.s006.zip › S6_File/Coverage Plots/05K4138DK1-Albo-Coverage.pdf]

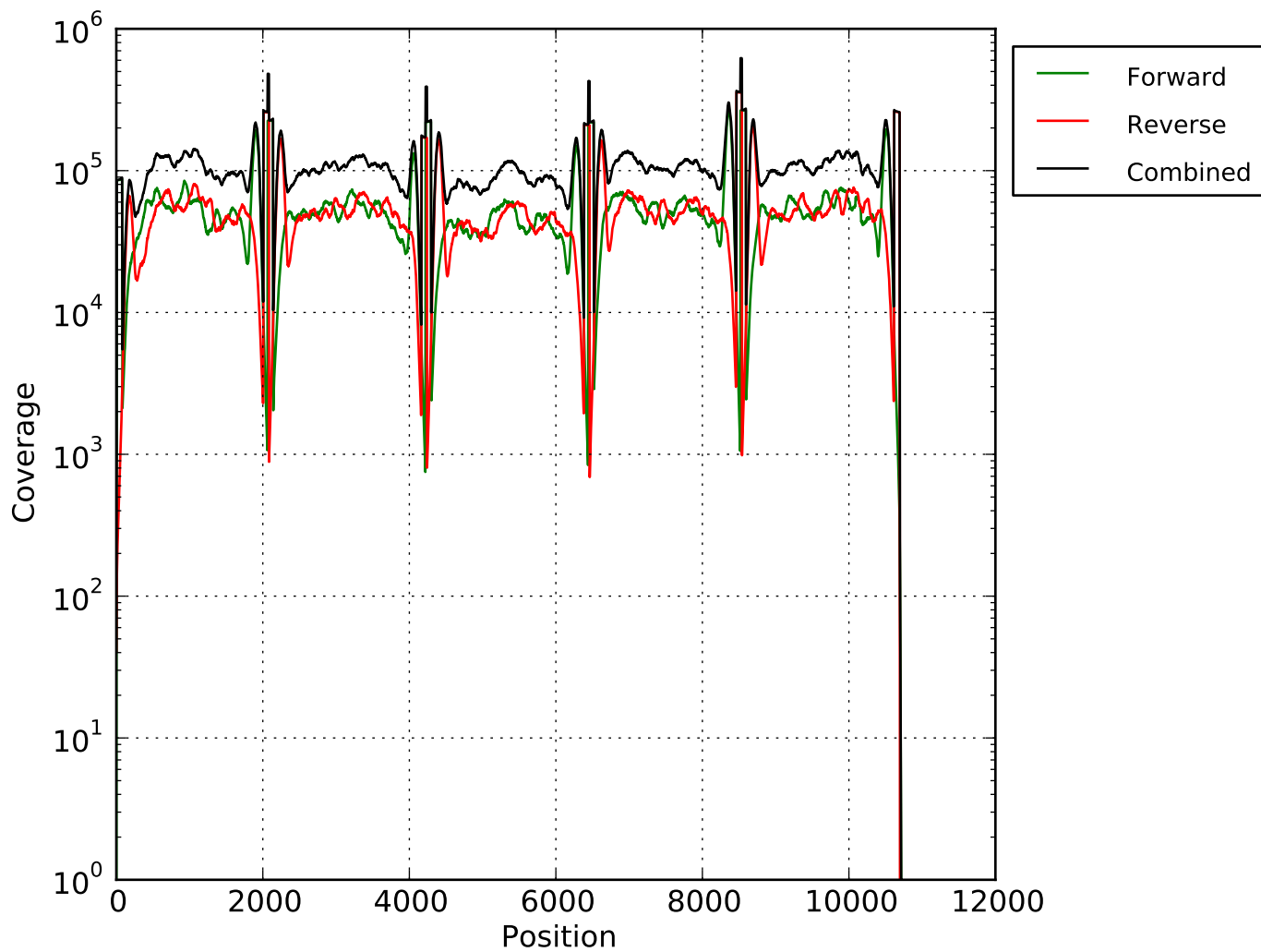

Supplement: S6 File — The LoFreq output.snp files for each sample in our data set are in the folder “SNP Files”. The read coverage graphs for each sample are in the folder “Coverage Plots”. (ZIP) [file pntd.0004044.s006.zip › S6_File/Coverage Plots/05K4138DK1-Serum-Coverage.pdf]

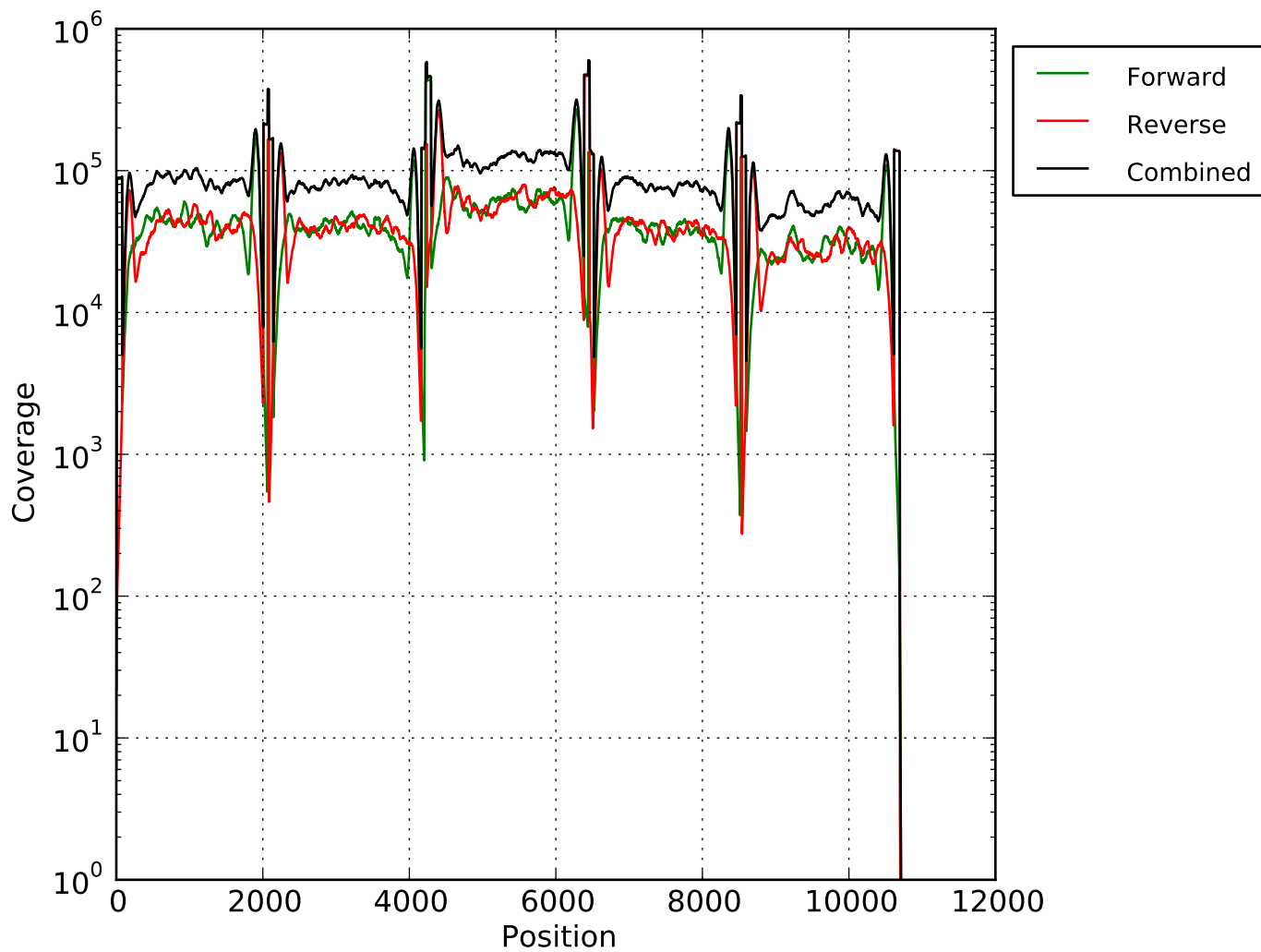

Supplement: S6 File — The LoFreq output.snp files for each sample in our data set are in the folder “SNP Files”. The read coverage graphs for each sample are in the folder “Coverage Plots”. (ZIP) [file pntd.0004044.s006.zip › S6_File/Coverage Plots/05K4138DK2-Aeg-Coverage.pdf]

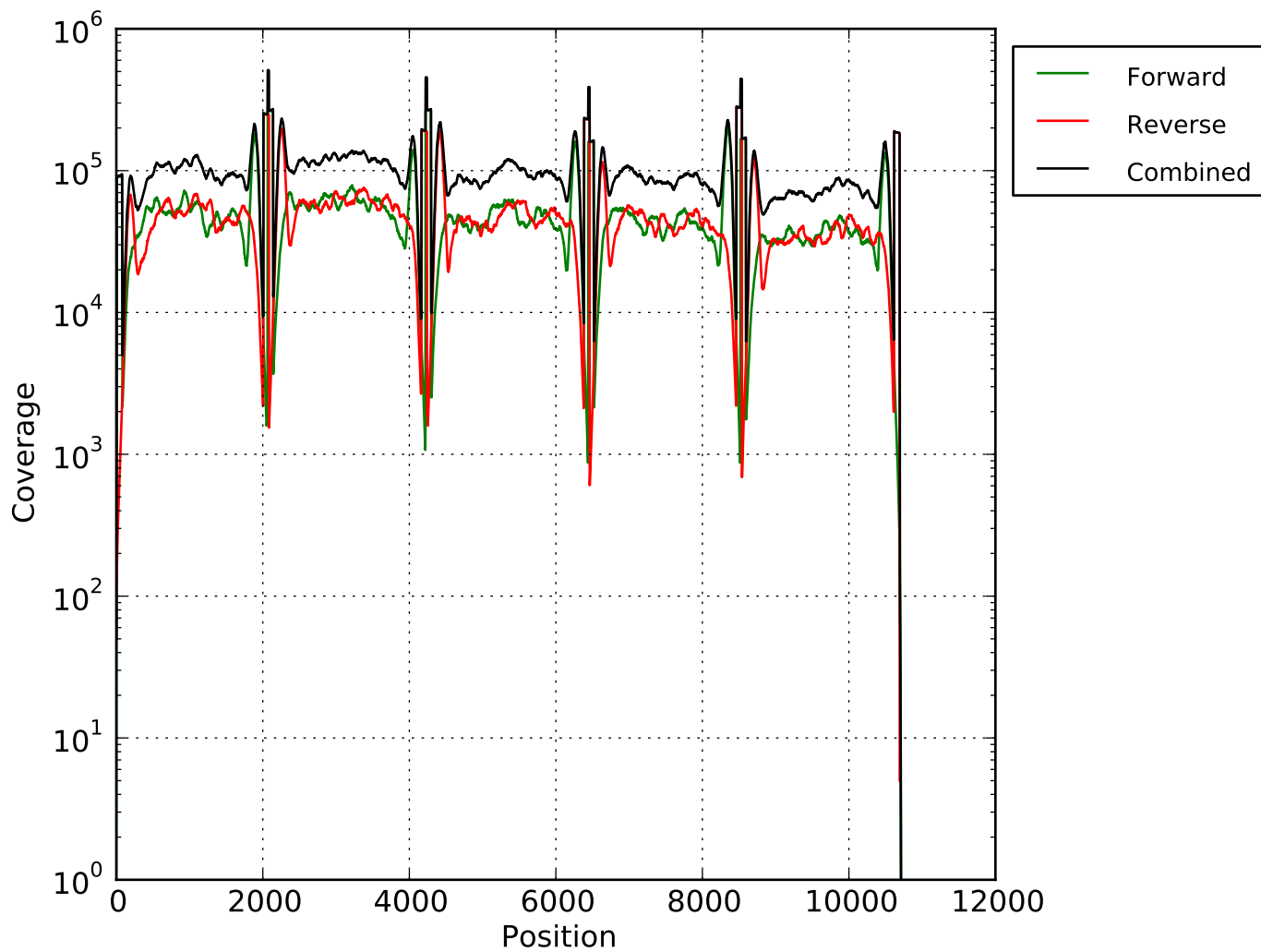

Supplement: S6 File — The LoFreq output.snp files for each sample in our data set are in the folder “SNP Files”. The read coverage graphs for each sample are in the folder “Coverage Plots”. (ZIP) [file pntd.0004044.s006.zip › S6_File/Coverage Plots/05K4138DK2-Albo-Coverage.pdf]

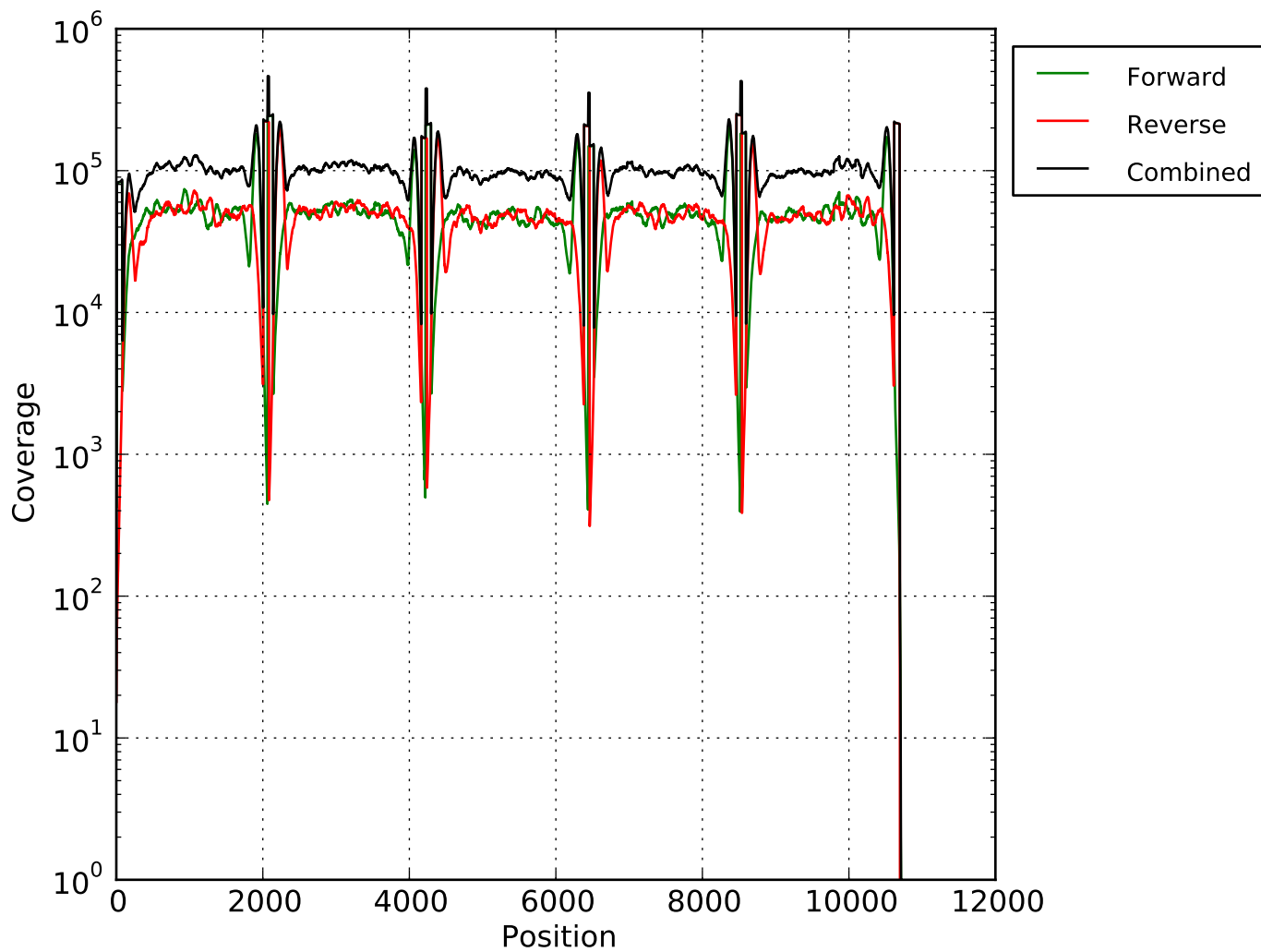

Supplement: S6 File — The LoFreq output.snp files for each sample in our data set are in the folder “SNP Files”. The read coverage graphs for each sample are in the folder “Coverage Plots”. (ZIP) [file pntd.0004044.s006.zip › S6_File/Coverage Plots/05K4138DK2-Serum-Coverage.pdf]

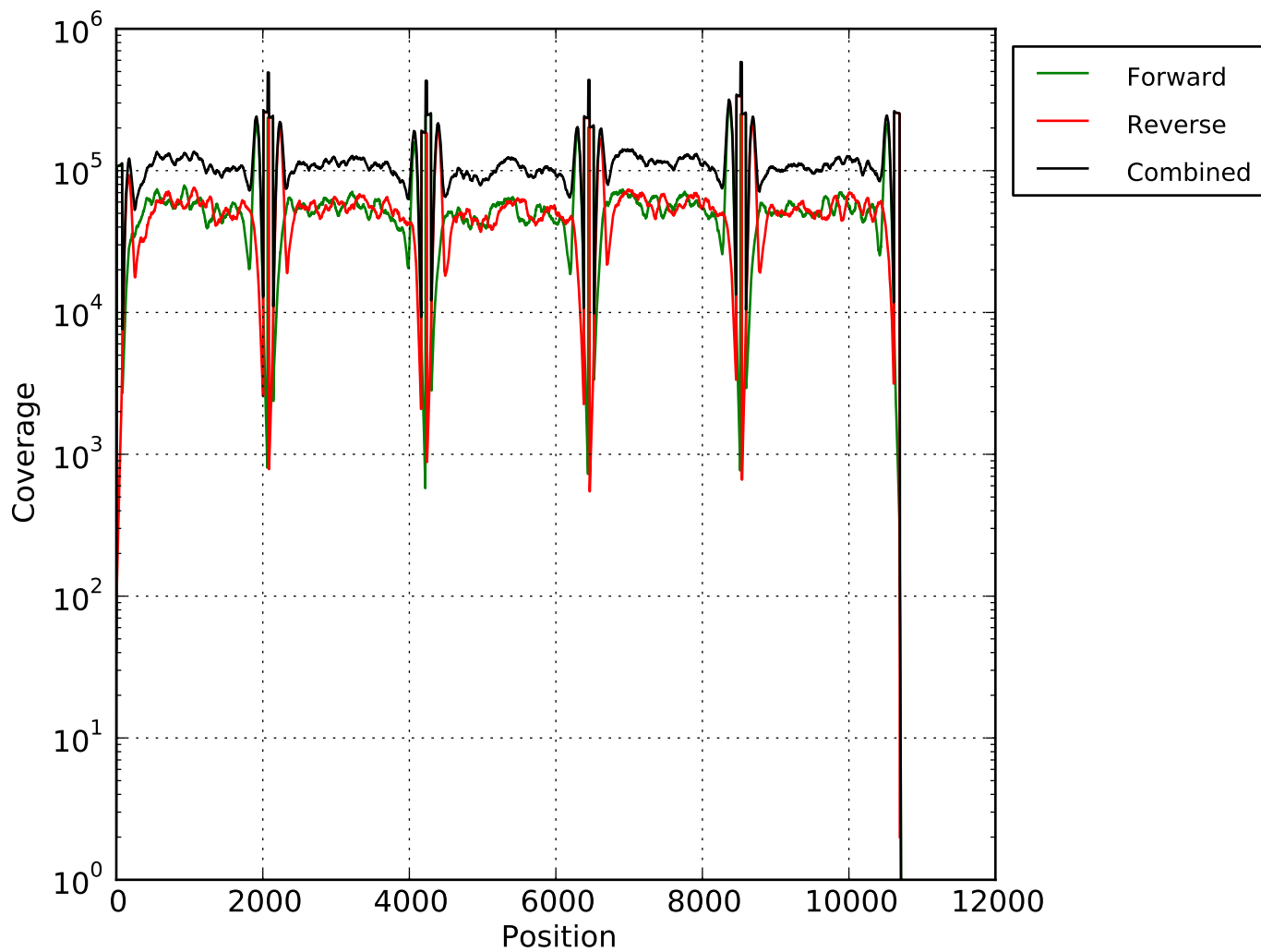

Supplement: S6 File — The LoFreq output.snp files for each sample in our data set are in the folder “SNP Files”. The read coverage graphs for each sample are in the folder “Coverage Plots”. (ZIP) [file pntd.0004044.s006.zip › S6_File/Coverage Plots/05K4139DK1-Aeg-Coverage.pdf]

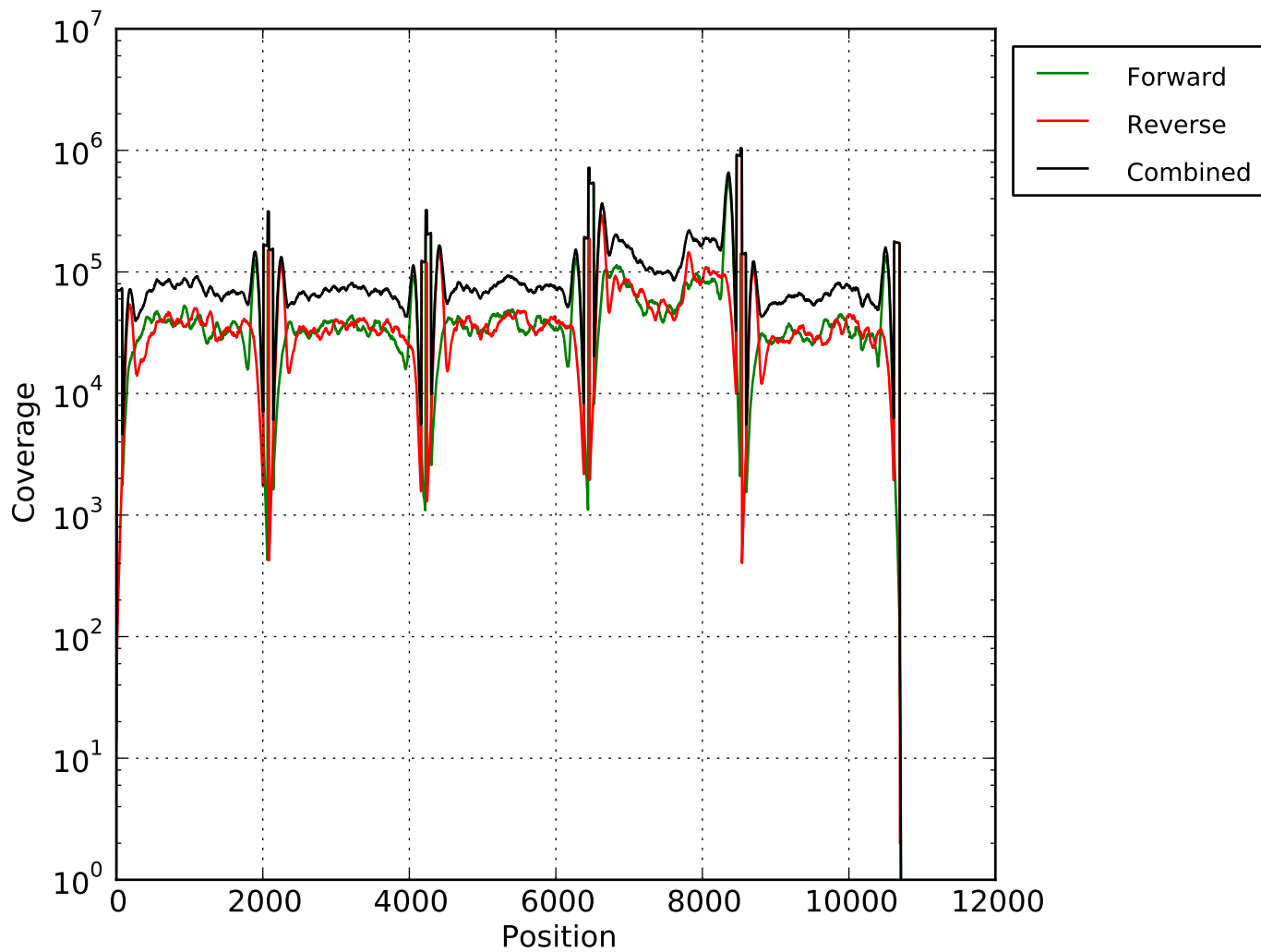

Supplement: S6 File — The LoFreq output.snp files for each sample in our data set are in the folder “SNP Files”. The read coverage graphs for each sample are in the folder “Coverage Plots”. (ZIP) [file pntd.0004044.s006.zip › S6_File/Coverage Plots/05K4139DK1-Albo-Coverage.pdf]

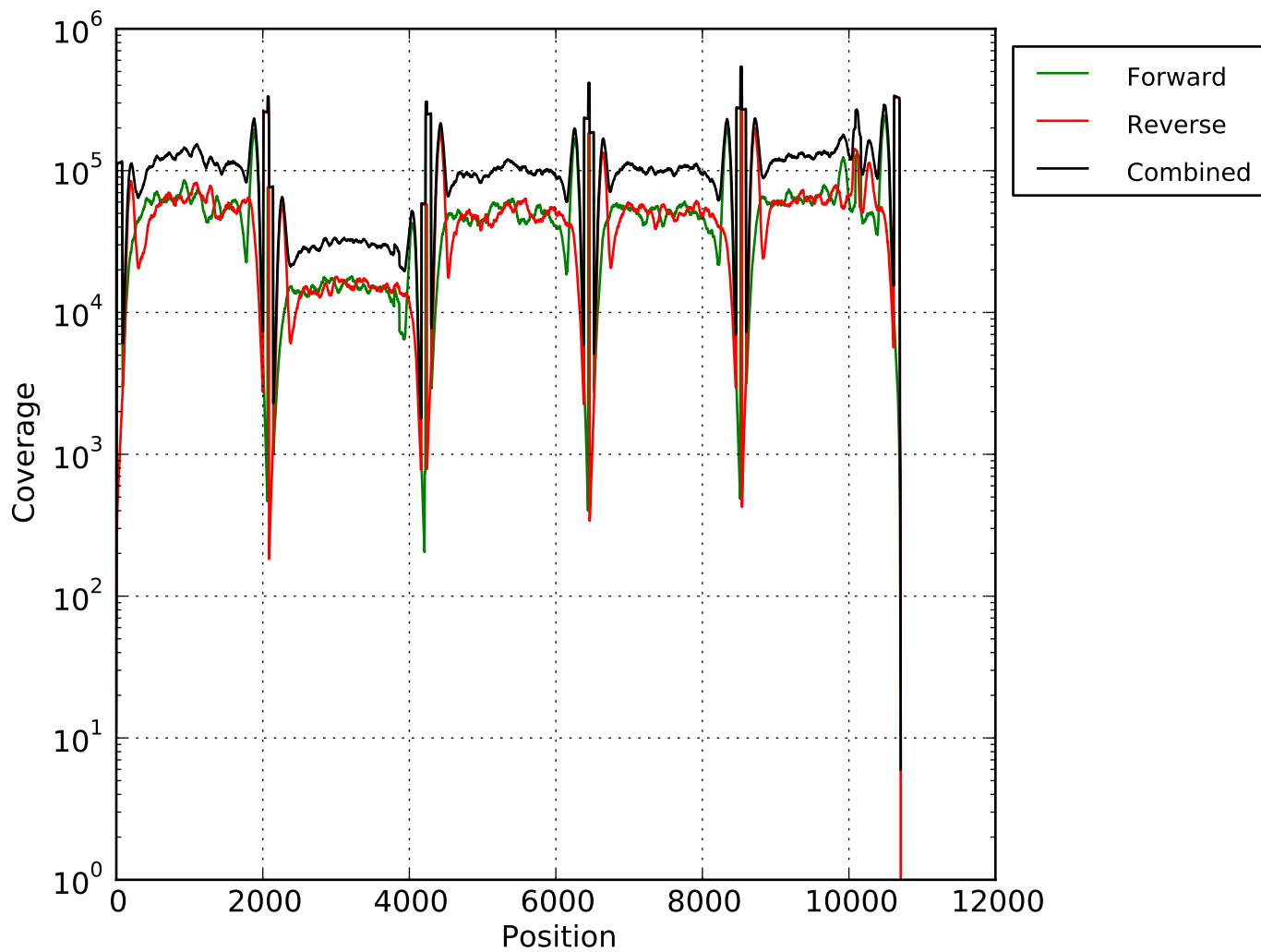

Supplement: S6 File — The LoFreq output.snp files for each sample in our data set are in the folder “SNP Files”. The read coverage graphs for each sample are in the folder “Coverage Plots”. (ZIP) [file pntd.0004044.s006.zip › S6_File/Coverage Plots/05K4139DK1-Serum-Coverage.pdf]

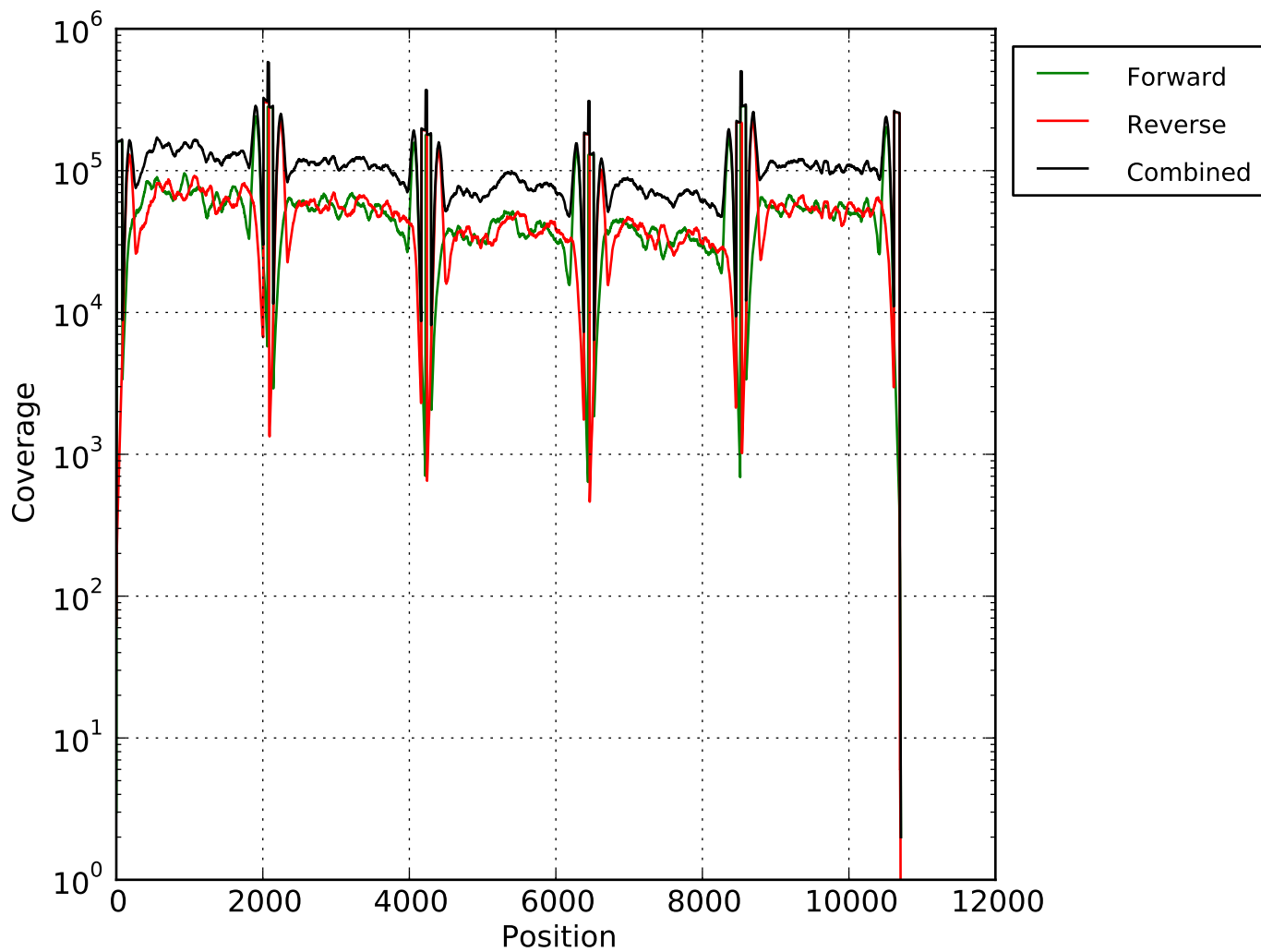

Supplement: S6 File — The LoFreq output.snp files for each sample in our data set are in the folder “SNP Files”. The read coverage graphs for each sample are in the folder “Coverage Plots”. (ZIP) [file pntd.0004044.s006.zip › S6_File/Coverage Plots/05K4139DK2-Aeg-Coverage.pdf]

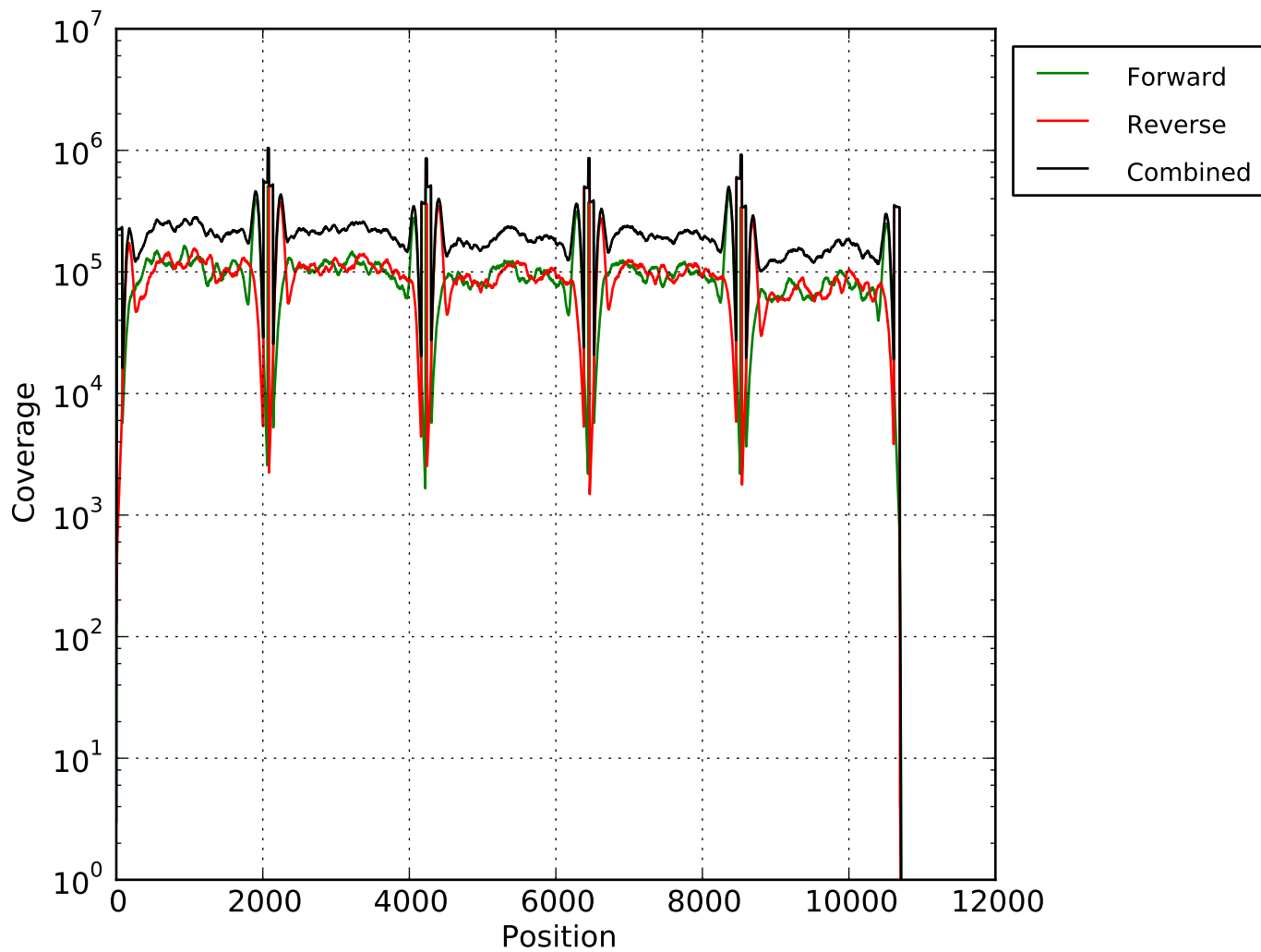

Supplement: S6 File — The LoFreq output.snp files for each sample in our data set are in the folder “SNP Files”. The read coverage graphs for each sample are in the folder “Coverage Plots”. (ZIP) [file pntd.0004044.s006.zip › S6_File/Coverage Plots/05K4139DK2-Albo-Coverage.pdf]

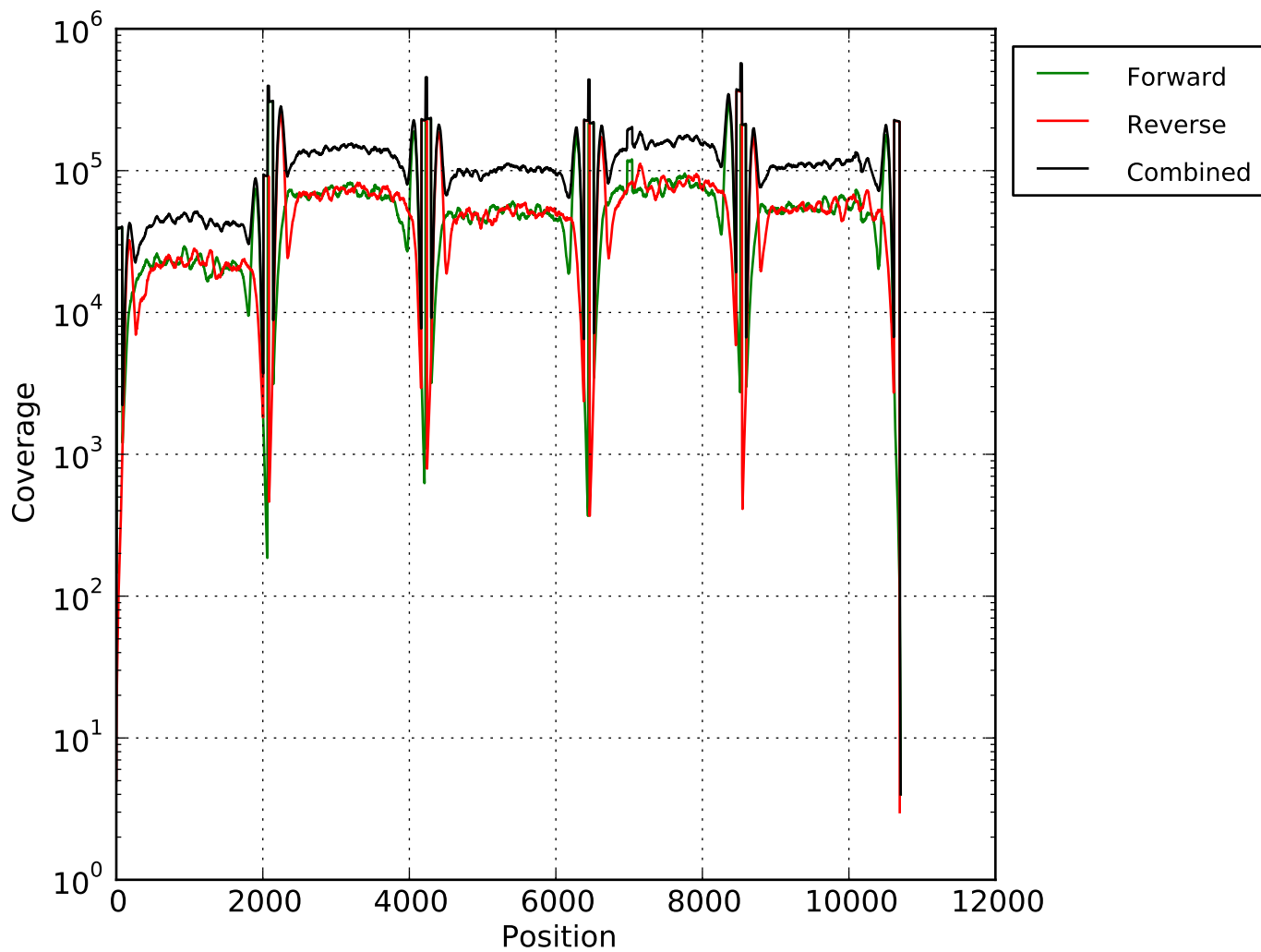

Supplement: S6 File — The LoFreq output.snp files for each sample in our data set are in the folder “SNP Files”. The read coverage graphs for each sample are in the folder “Coverage Plots”. (ZIP) [file pntd.0004044.s006.zip › S6_File/Coverage Plots/05K4139DK2-Serum-Coverage.pdf]

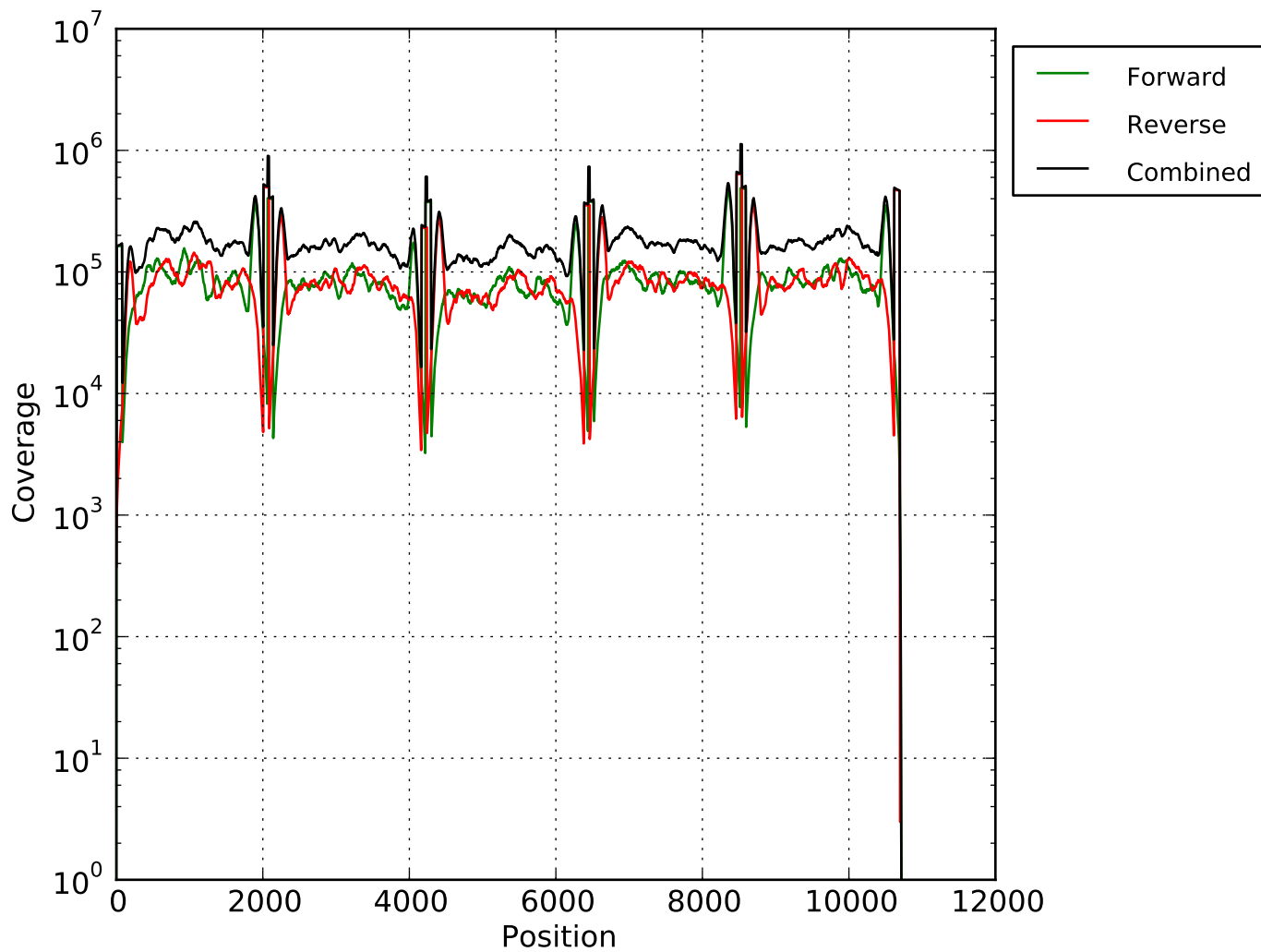

Supplement: S6 File — The LoFreq output.snp files for each sample in our data set are in the folder “SNP Files”. The read coverage graphs for each sample are in the folder “Coverage Plots”. (ZIP) [file pntd.0004044.s006.zip › S6_File/Coverage Plots/05K4152DK1-Aeg-Coverage.pdf]

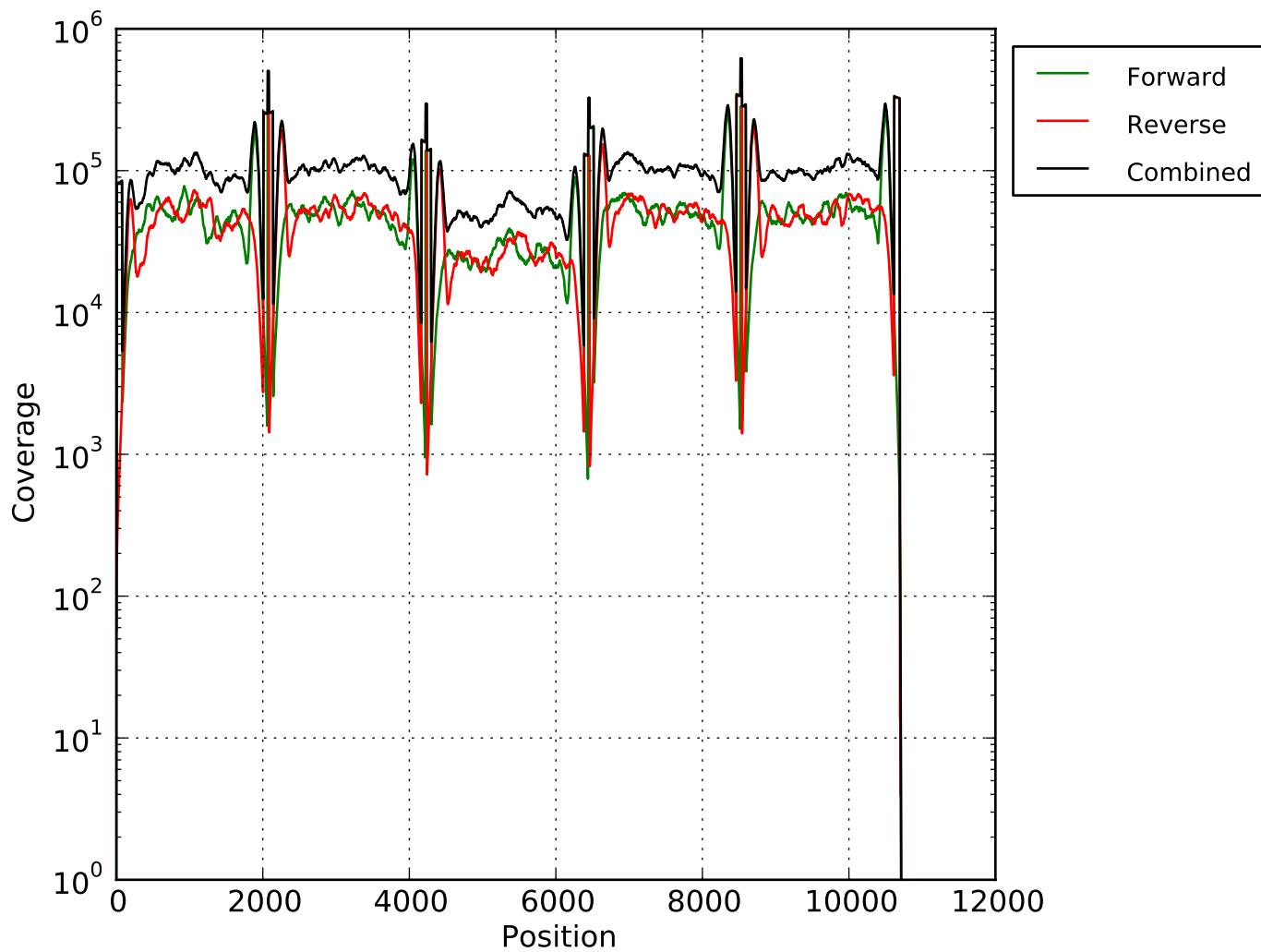

Supplement: S6 File — The LoFreq output.snp files for each sample in our data set are in the folder “SNP Files”. The read coverage graphs for each sample are in the folder “Coverage Plots”. (ZIP) [file pntd.0004044.s006.zip › S6_File/Coverage Plots/05K4152DK1-Albo-Coverage.pdf]

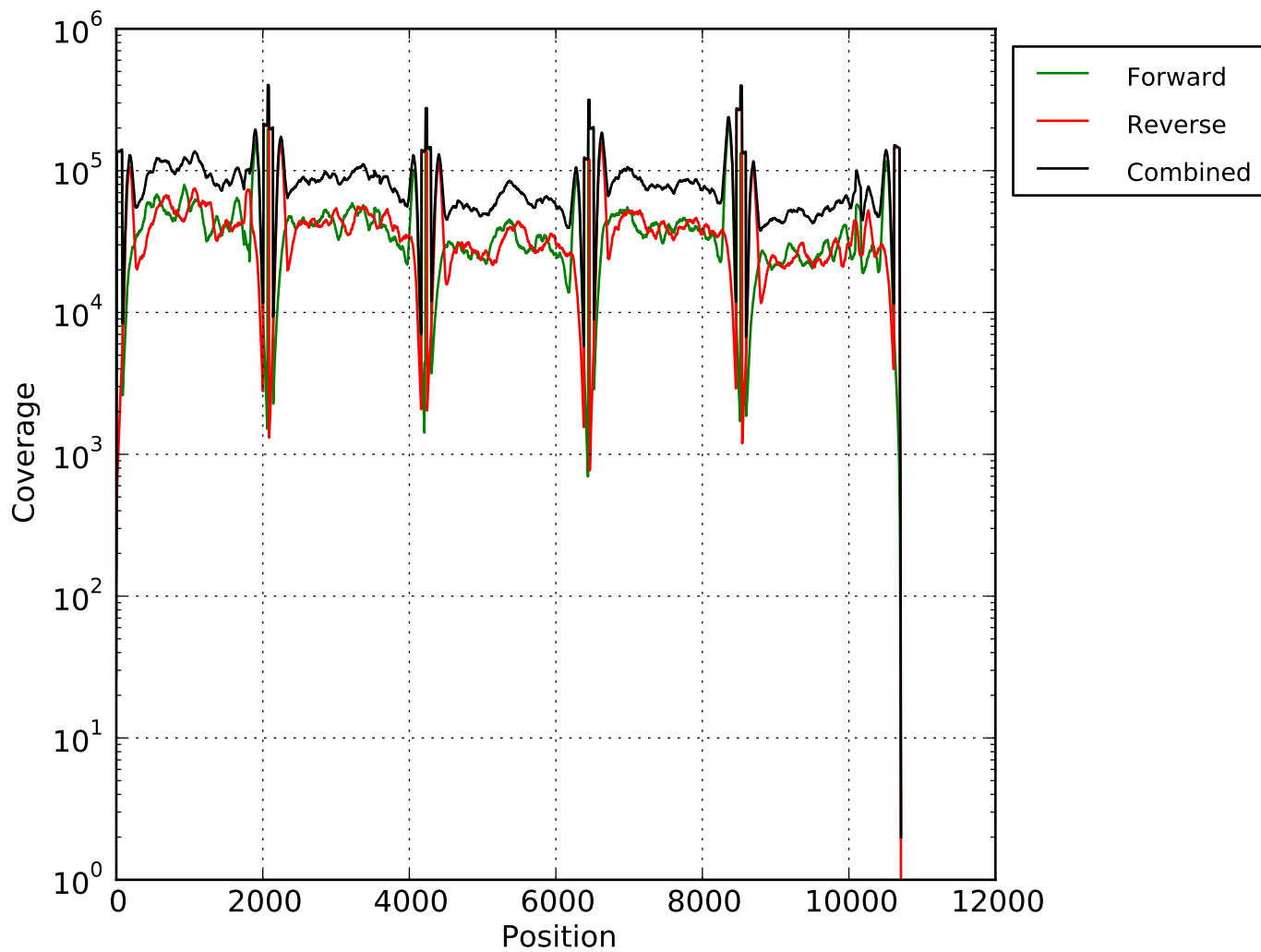

Supplement: S6 File — The LoFreq output.snp files for each sample in our data set are in the folder “SNP Files”. The read coverage graphs for each sample are in the folder “Coverage Plots”. (ZIP) [file pntd.0004044.s006.zip › S6_File/Coverage Plots/05K4152DK1-Serum-Coverage.pdf]

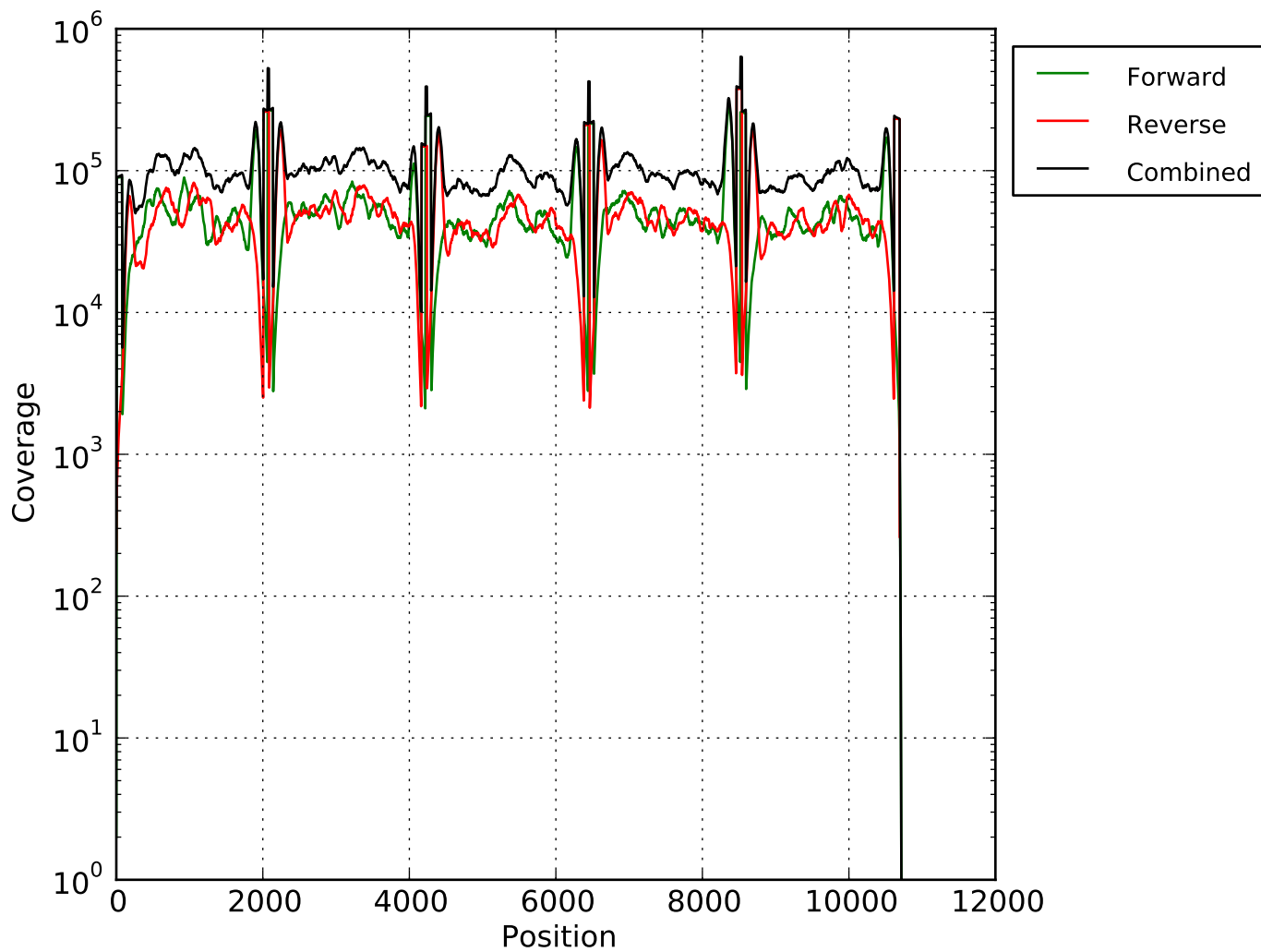

Supplement: S6 File — The LoFreq output.snp files for each sample in our data set are in the folder “SNP Files”. The read coverage graphs for each sample are in the folder “Coverage Plots”. (ZIP) [file pntd.0004044.s006.zip › S6_File/Coverage Plots/05K4152DK2-Aeg-Coverage.pdf]

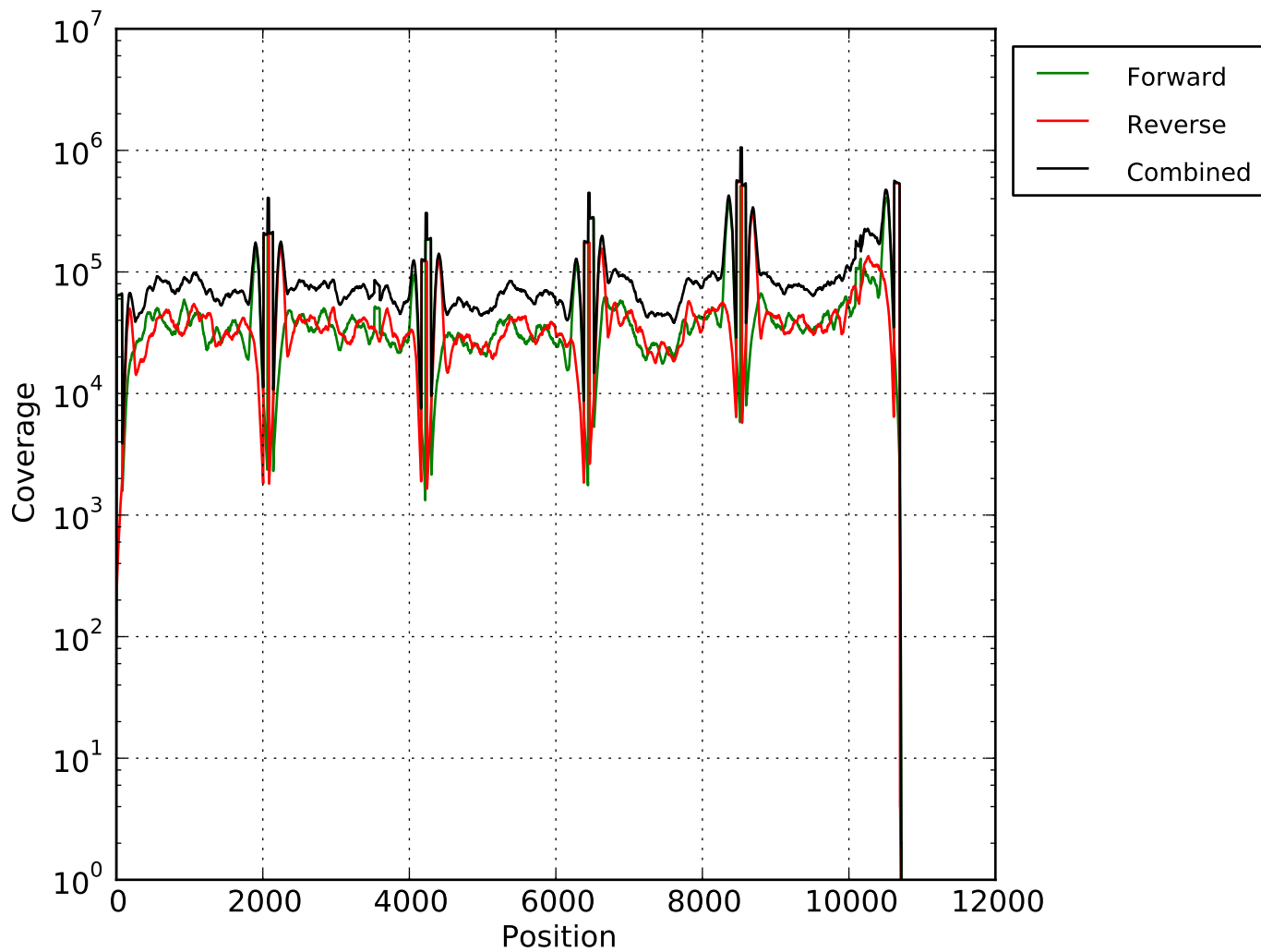

Supplement: S6 File — The LoFreq output.snp files for each sample in our data set are in the folder “SNP Files”. The read coverage graphs for each sample are in the folder “Coverage Plots”. (ZIP) [file pntd.0004044.s006.zip › S6_File/Coverage Plots/05K4152DK2-Albo-Coverage.pdf]

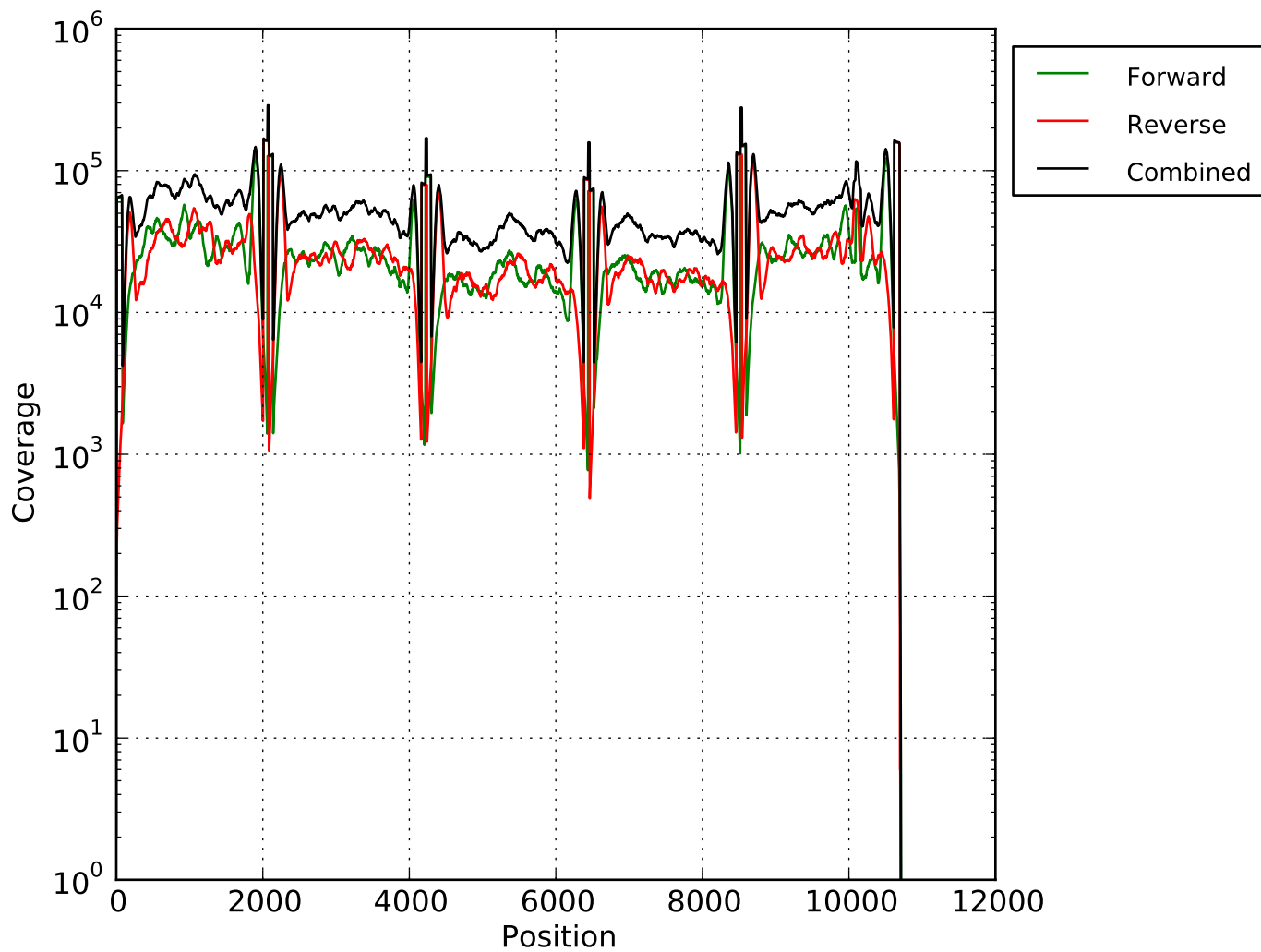

Supplement: S6 File — The LoFreq output.snp files for each sample in our data set are in the folder “SNP Files”. The read coverage graphs for each sample are in the folder “Coverage Plots”. (ZIP) [file pntd.0004044.s006.zip › S6_File/Coverage Plots/05K4152DK2-Serum-Coverage.pdf]

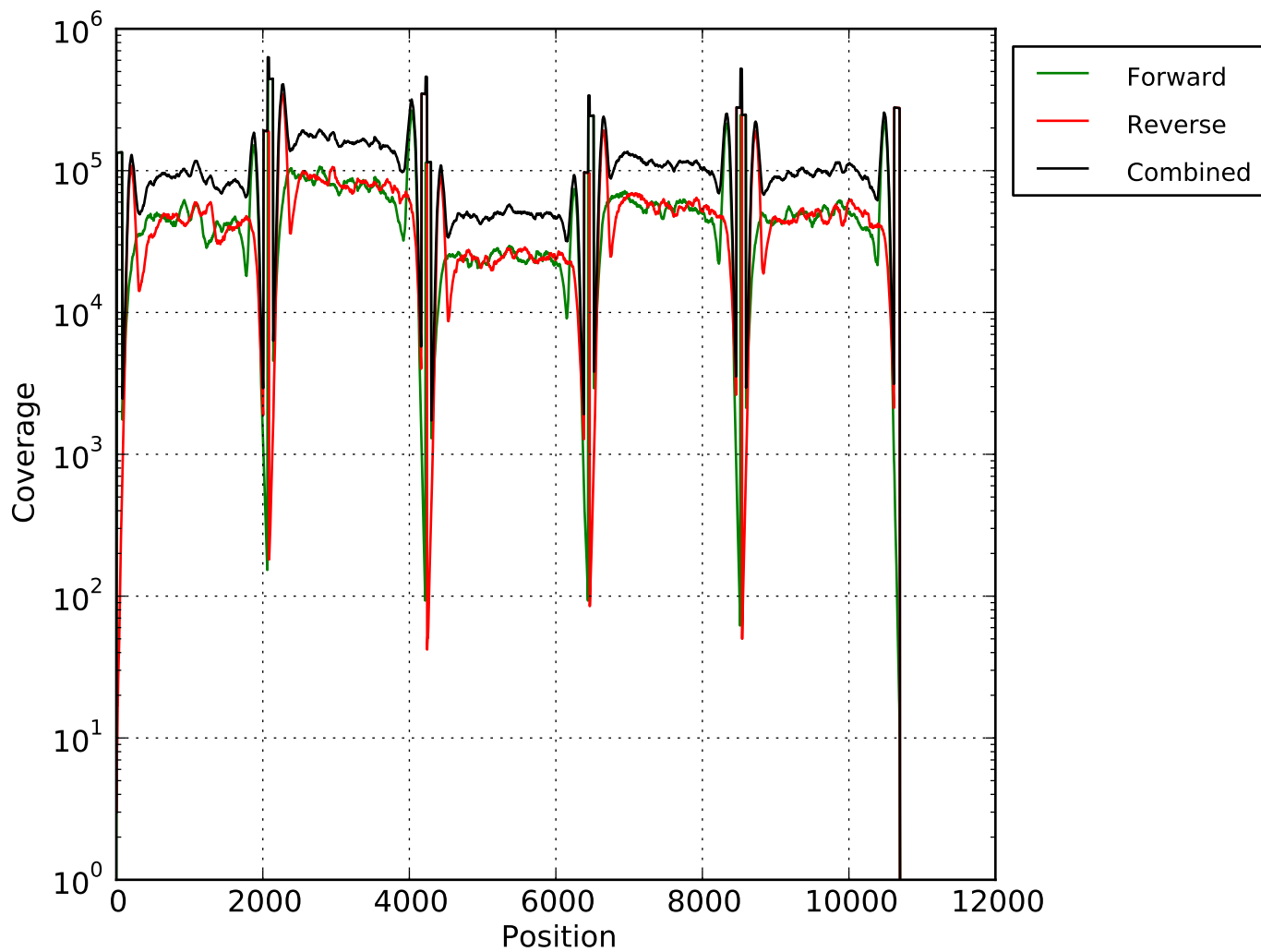

Supplement: S6 File — The LoFreq output.snp files for each sample in our data set are in the folder “SNP Files”. The read coverage graphs for each sample are in the folder “Coverage Plots”. (ZIP) [file pntd.0004044.s006.zip › S6_File/Coverage Plots/05K4172DK1-Aeg-Coverage.pdf]

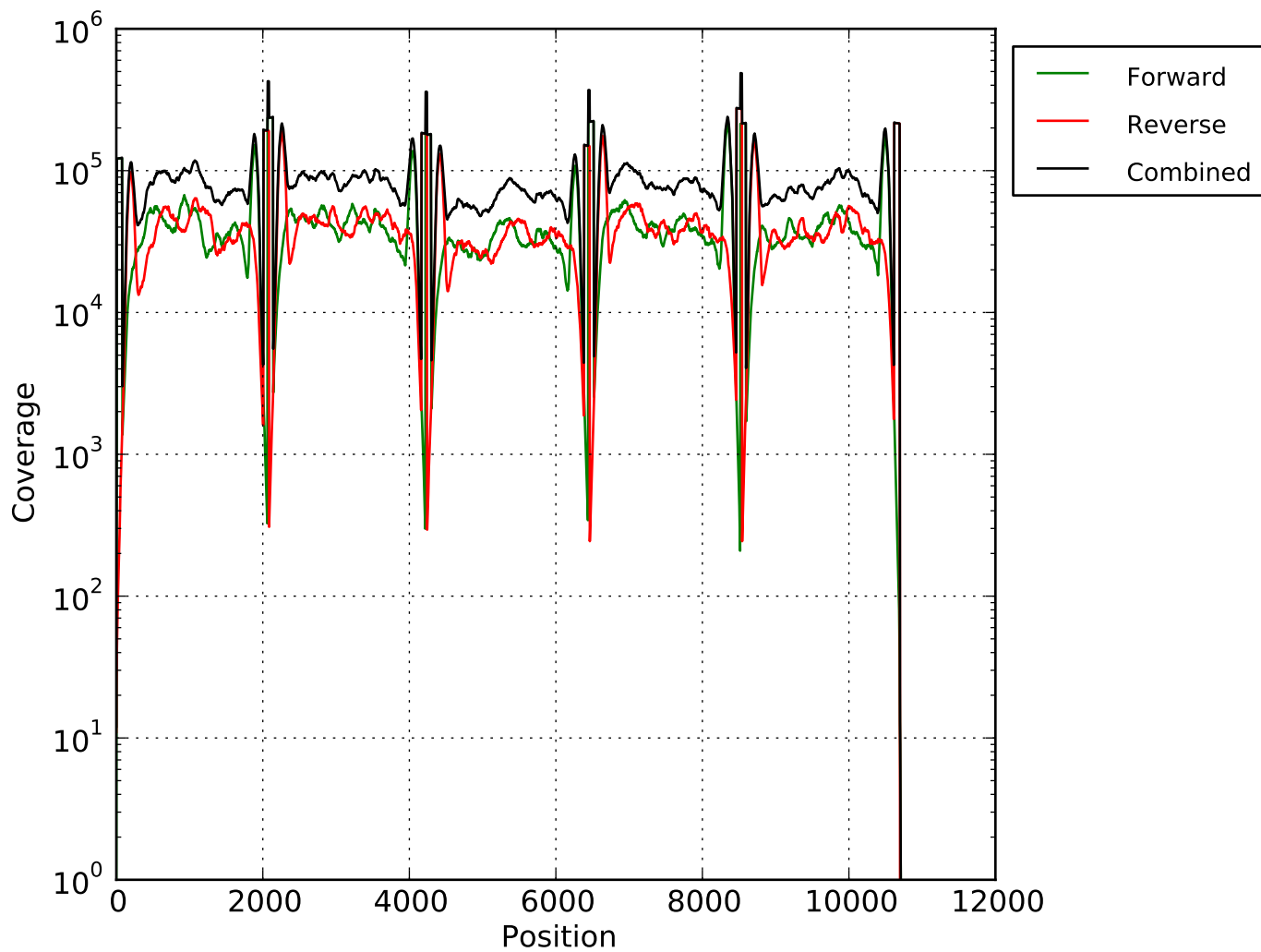

Supplement: S6 File — The LoFreq output.snp files for each sample in our data set are in the folder “SNP Files”. The read coverage graphs for each sample are in the folder “Coverage Plots”. (ZIP) [file pntd.0004044.s006.zip › S6_File/Coverage Plots/05K4172DK1-Albo-Coverage.pdf]

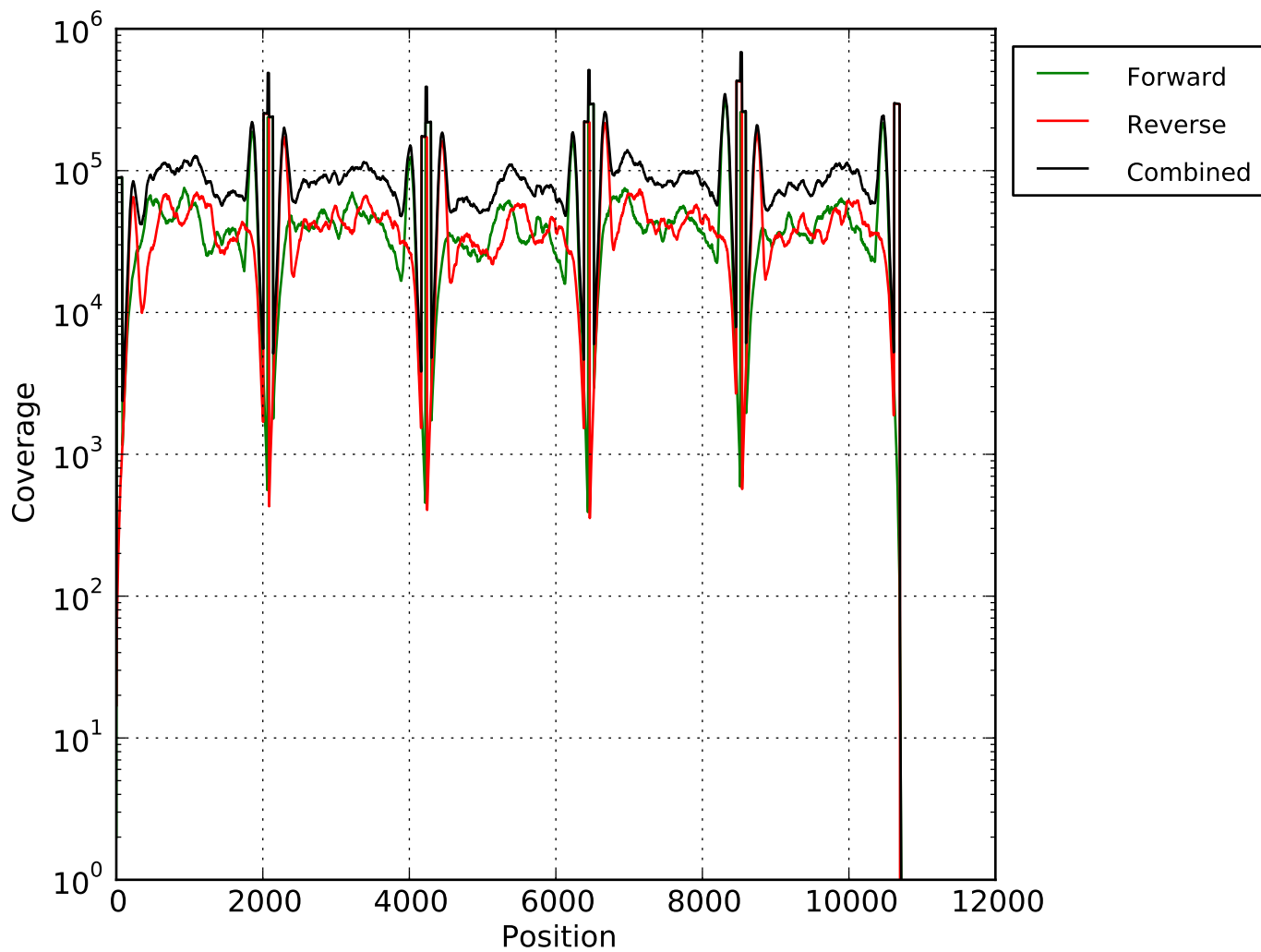

Supplement: S6 File — The LoFreq output.snp files for each sample in our data set are in the folder “SNP Files”. The read coverage graphs for each sample are in the folder “Coverage Plots”. (ZIP) [file pntd.0004044.s006.zip › S6_File/Coverage Plots/05K4172DK2-Aeg-Coverage.pdf]

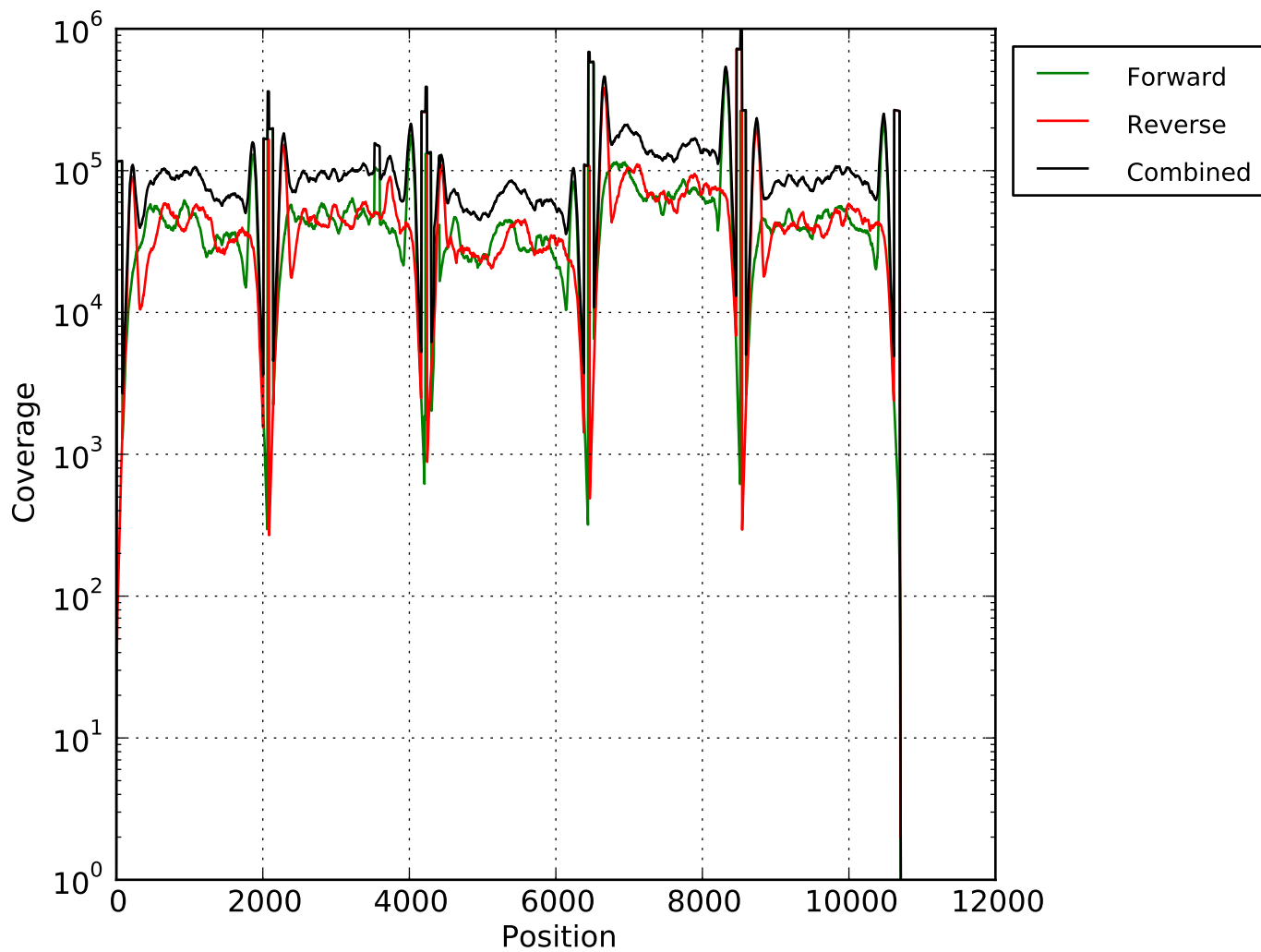

Supplement: S6 File — The LoFreq output.snp files for each sample in our data set are in the folder “SNP Files”. The read coverage graphs for each sample are in the folder “Coverage Plots”. (ZIP) [file pntd.0004044.s006.zip › S6_File/Coverage Plots/05K4172DK2-Albo-Coverage.pdf]

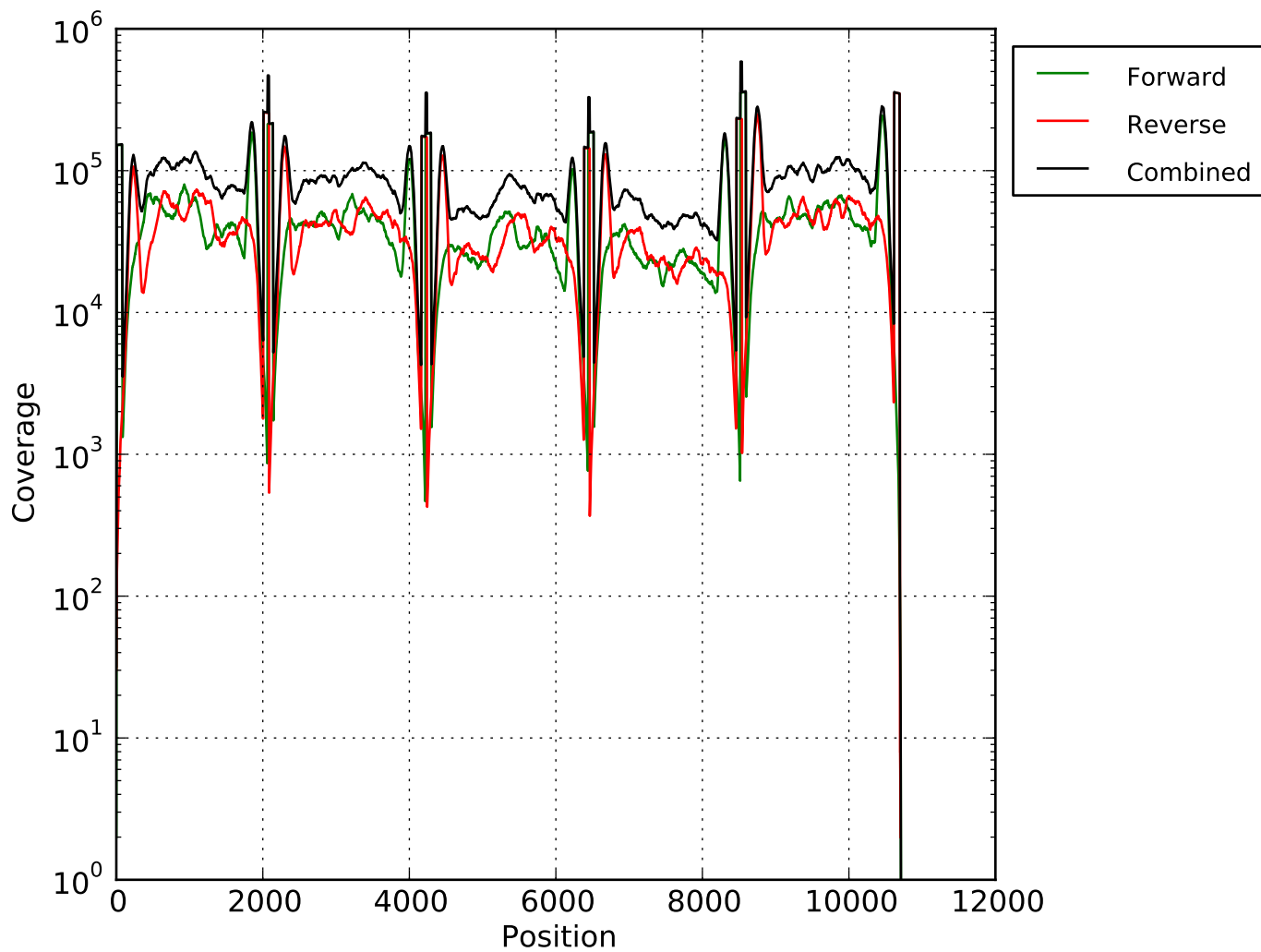

Supplement: S6 File — The LoFreq output.snp files for each sample in our data set are in the folder “SNP Files”. The read coverage graphs for each sample are in the folder “Coverage Plots”. (ZIP) [file pntd.0004044.s006.zip › S6_File/Coverage Plots/05K4173DK1-Aeg-Coverage.pdf]

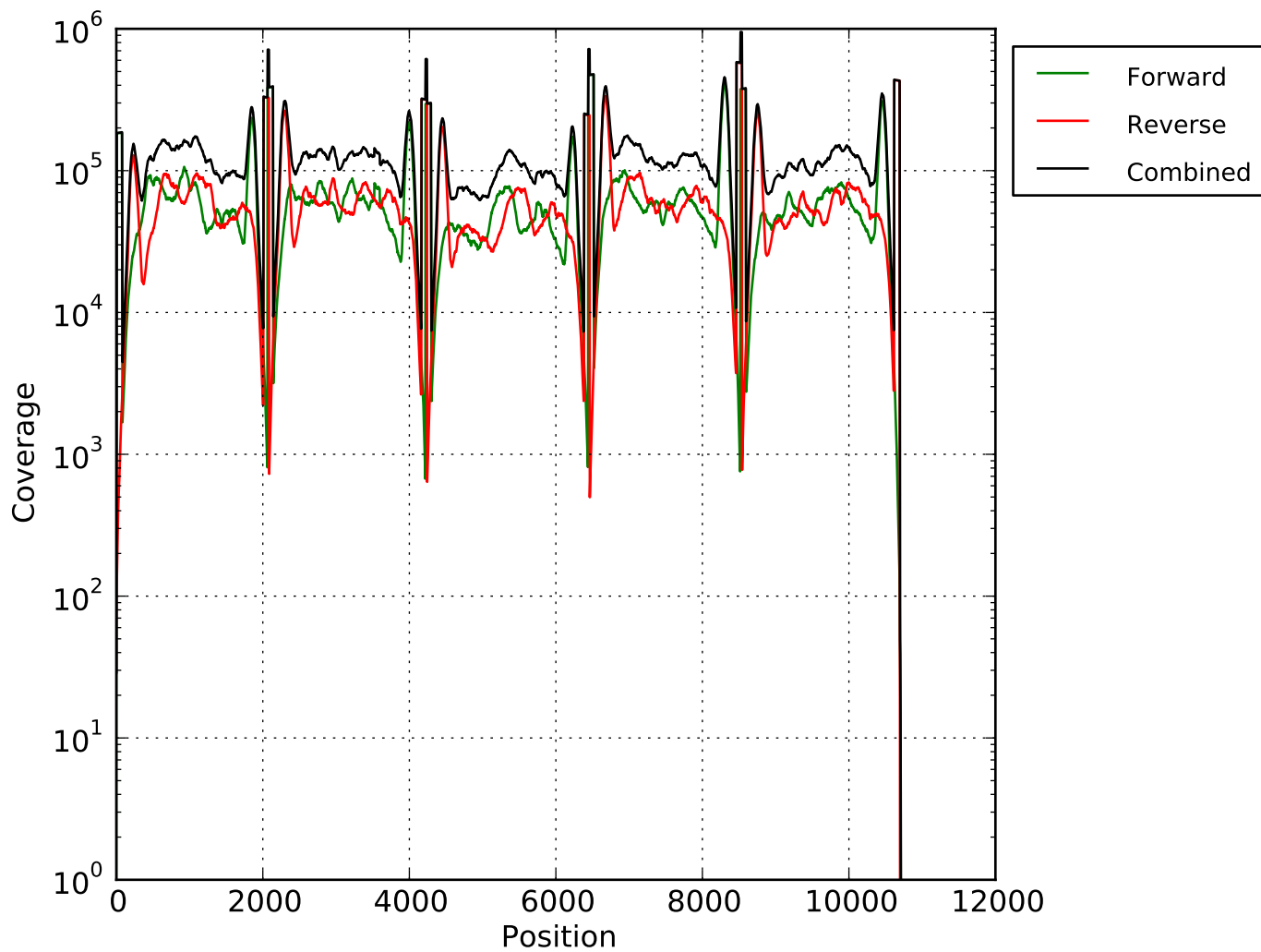

Supplement: S6 File — The LoFreq output.snp files for each sample in our data set are in the folder “SNP Files”. The read coverage graphs for each sample are in the folder “Coverage Plots”. (ZIP) [file pntd.0004044.s006.zip › S6_File/Coverage Plots/05K4173DK1-Albo-Coverage.pdf]

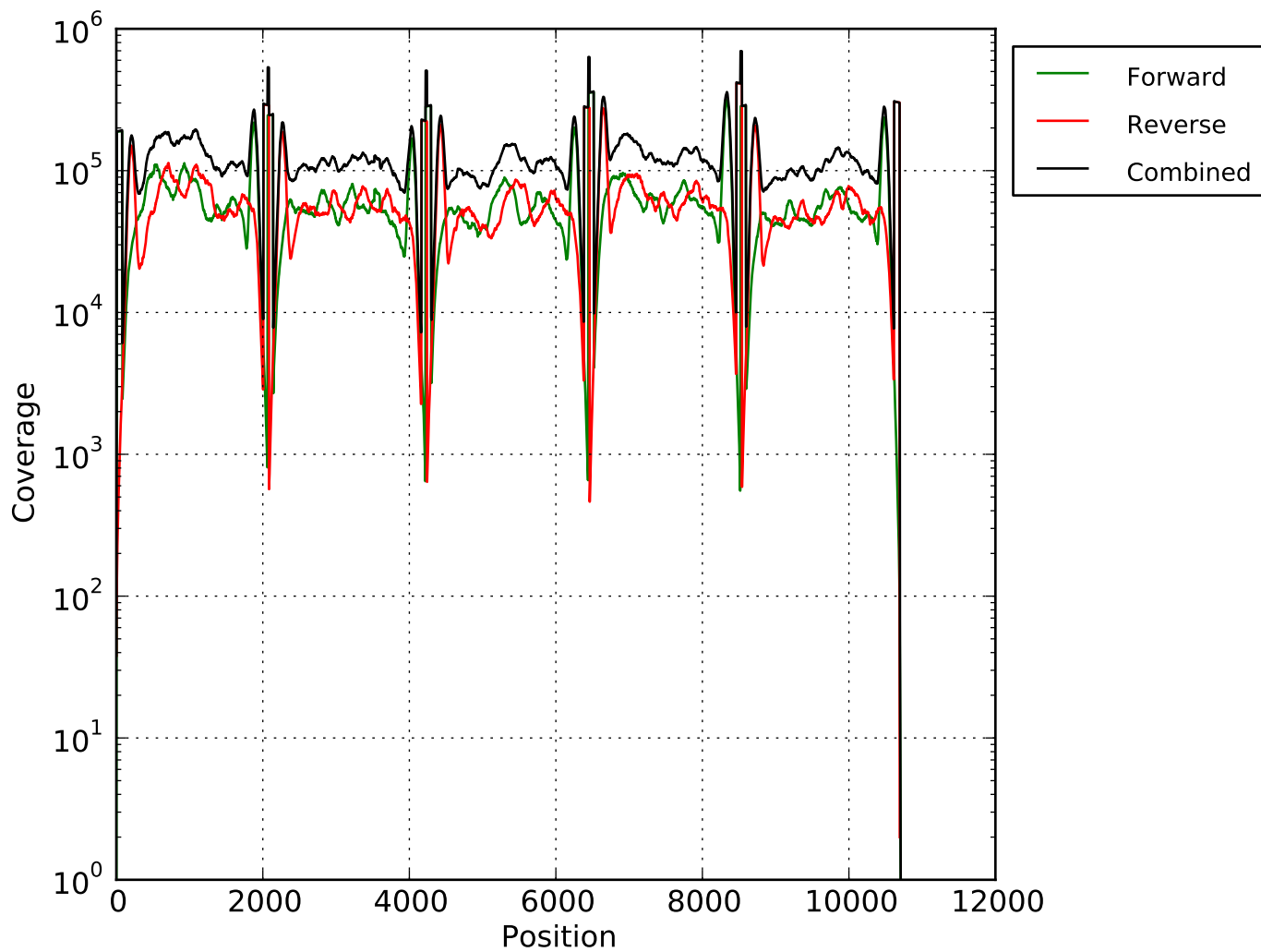

Supplement: S6 File — The LoFreq output.snp files for each sample in our data set are in the folder “SNP Files”. The read coverage graphs for each sample are in the folder “Coverage Plots”. (ZIP) [file pntd.0004044.s006.zip › S6_File/Coverage Plots/05K4173DK2-Aeg-Coverage.pdf]

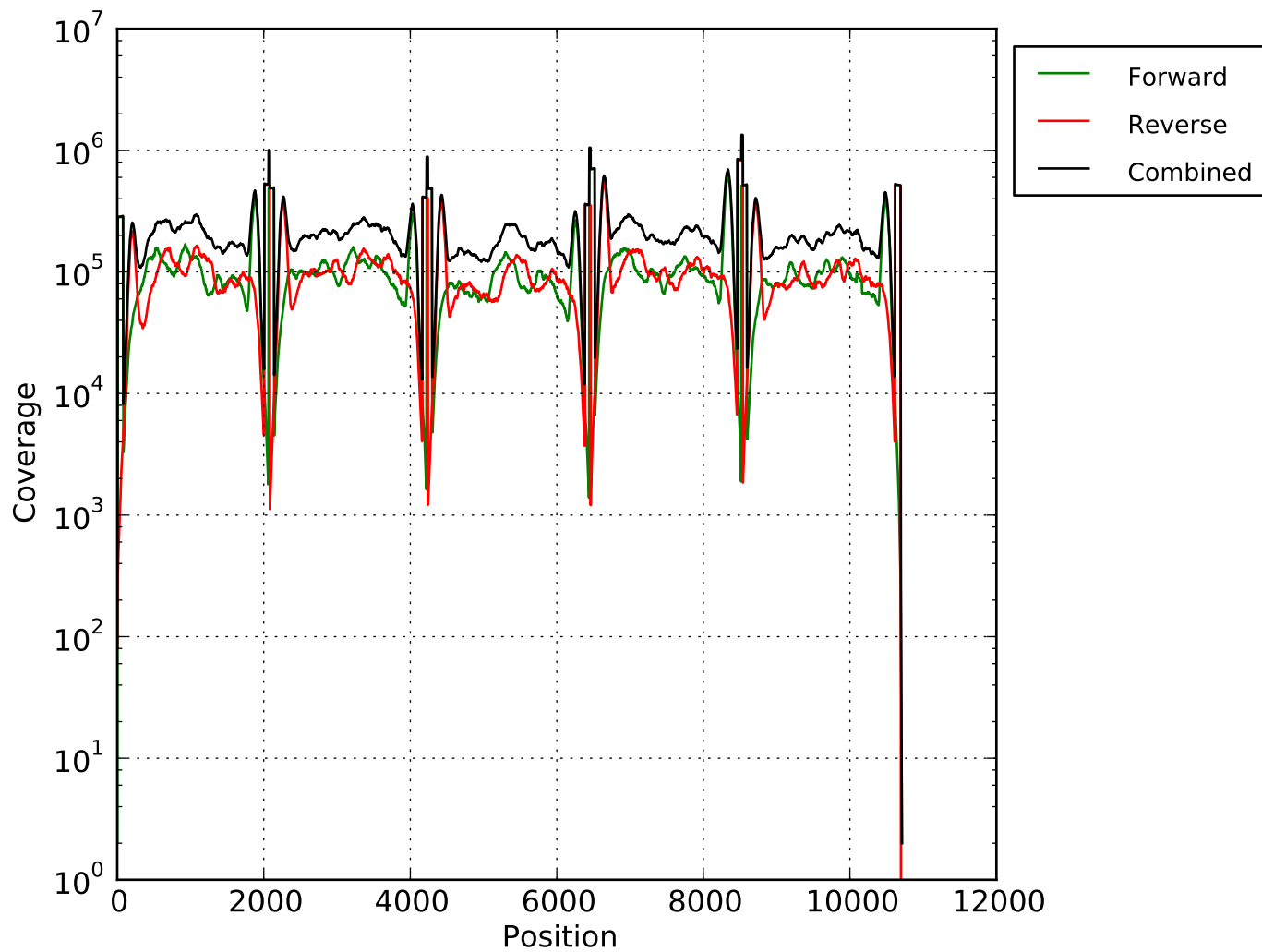

Supplement: S6 File — The LoFreq output.snp files for each sample in our data set are in the folder “SNP Files”. The read coverage graphs for each sample are in the folder “Coverage Plots”. (ZIP) [file pntd.0004044.s006.zip › S6_File/Coverage Plots/05K4173DK2-Albo-Coverage.pdf]

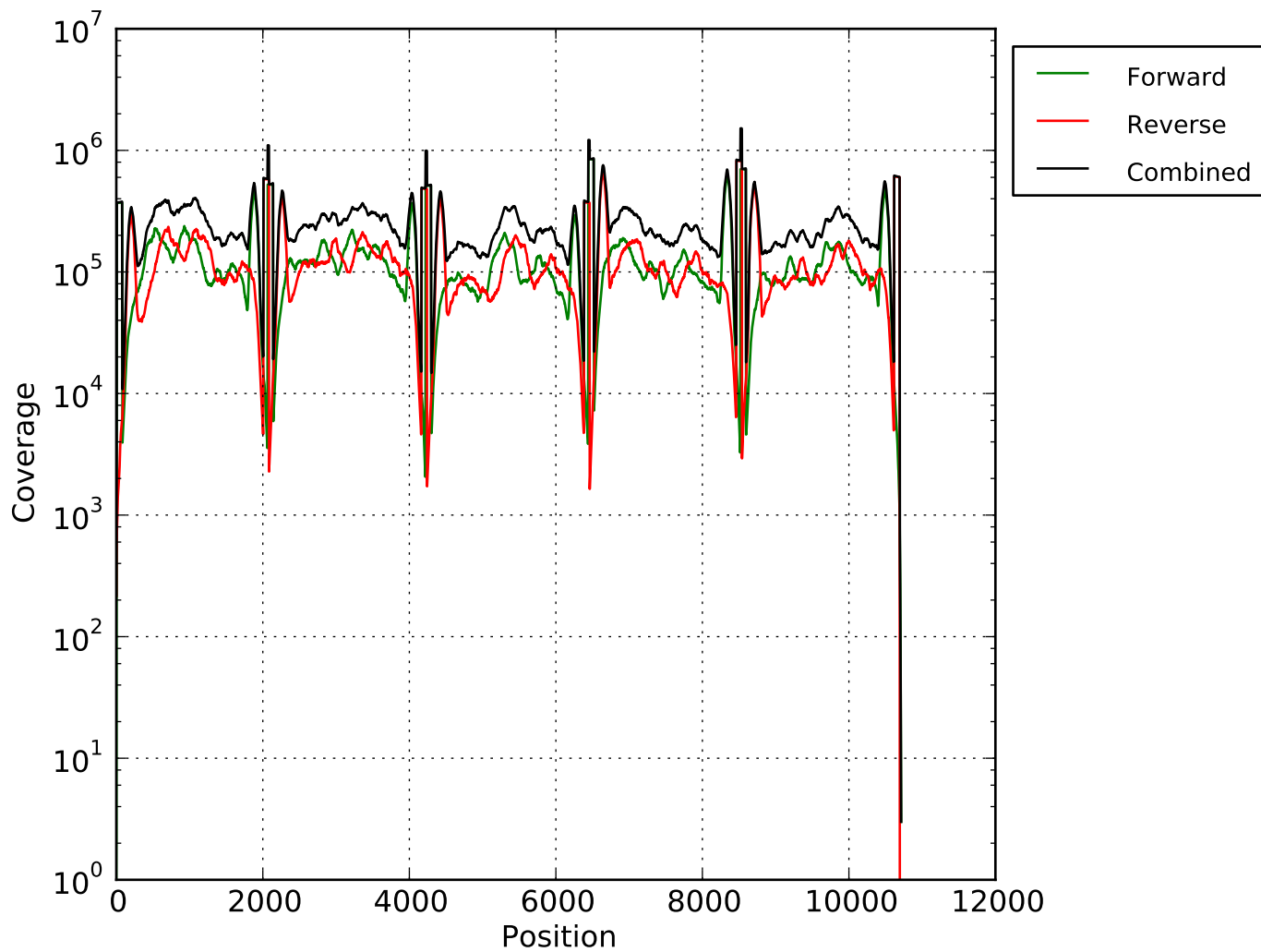

Supplement: S6 File — The LoFreq output.snp files for each sample in our data set are in the folder “SNP Files”. The read coverage graphs for each sample are in the folder “Coverage Plots”. (ZIP) [file pntd.0004044.s006.zip › S6_File/Coverage Plots/05K4441DK1-Aeg-Coverage.pdf]

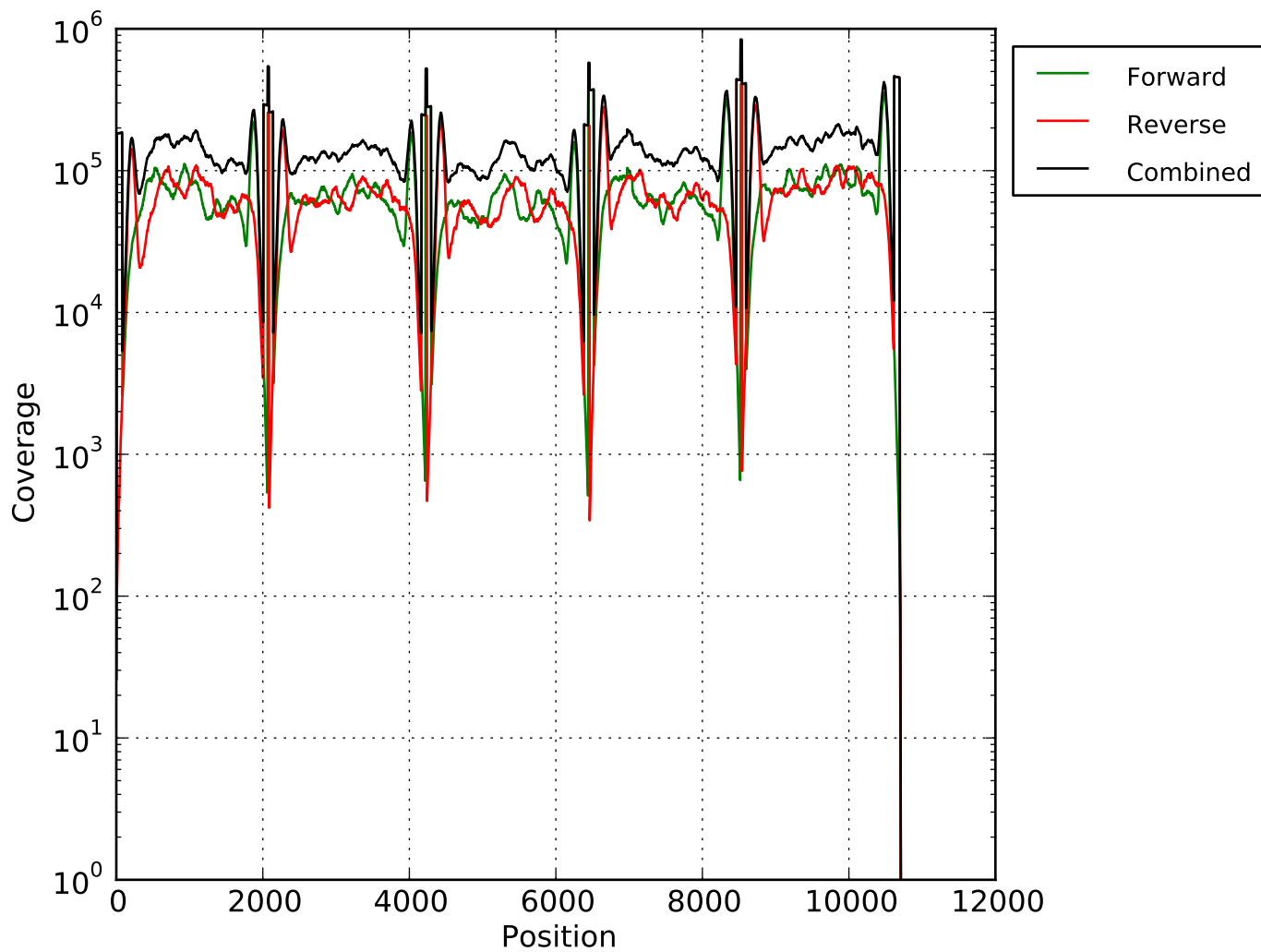

Supplement: S6 File — The LoFreq output.snp files for each sample in our data set are in the folder “SNP Files”. The read coverage graphs for each sample are in the folder “Coverage Plots”. (ZIP) [file pntd.0004044.s006.zip › S6_File/Coverage Plots/05K4441DK1-Albo-Coverage.pdf]

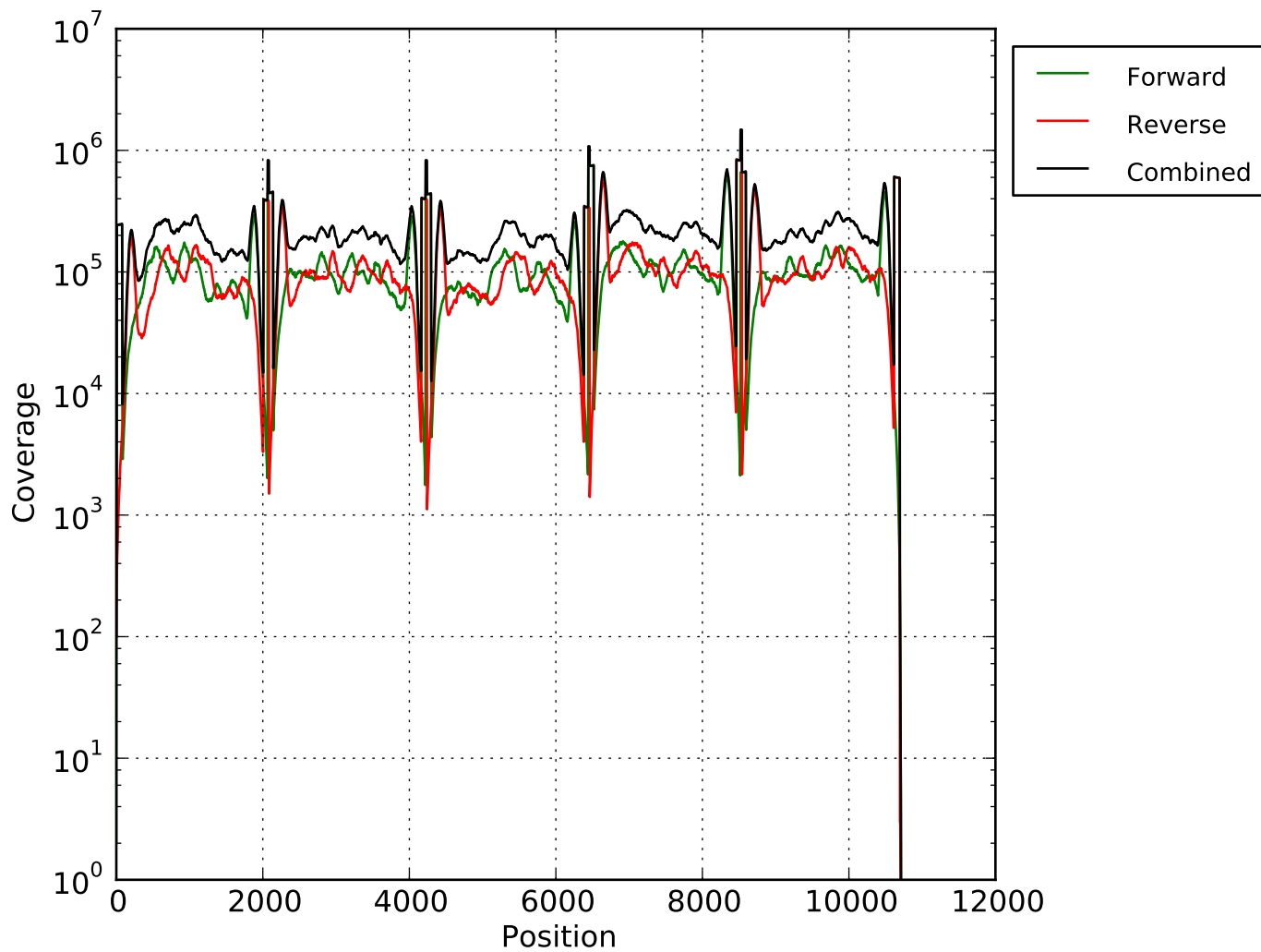

Supplement: S6 File — The LoFreq output.snp files for each sample in our data set are in the folder “SNP Files”. The read coverage graphs for each sample are in the folder “Coverage Plots”. (ZIP) [file pntd.0004044.s006.zip › S6_File/Coverage Plots/05K4441DK1-Serum-Coverage.pdf]

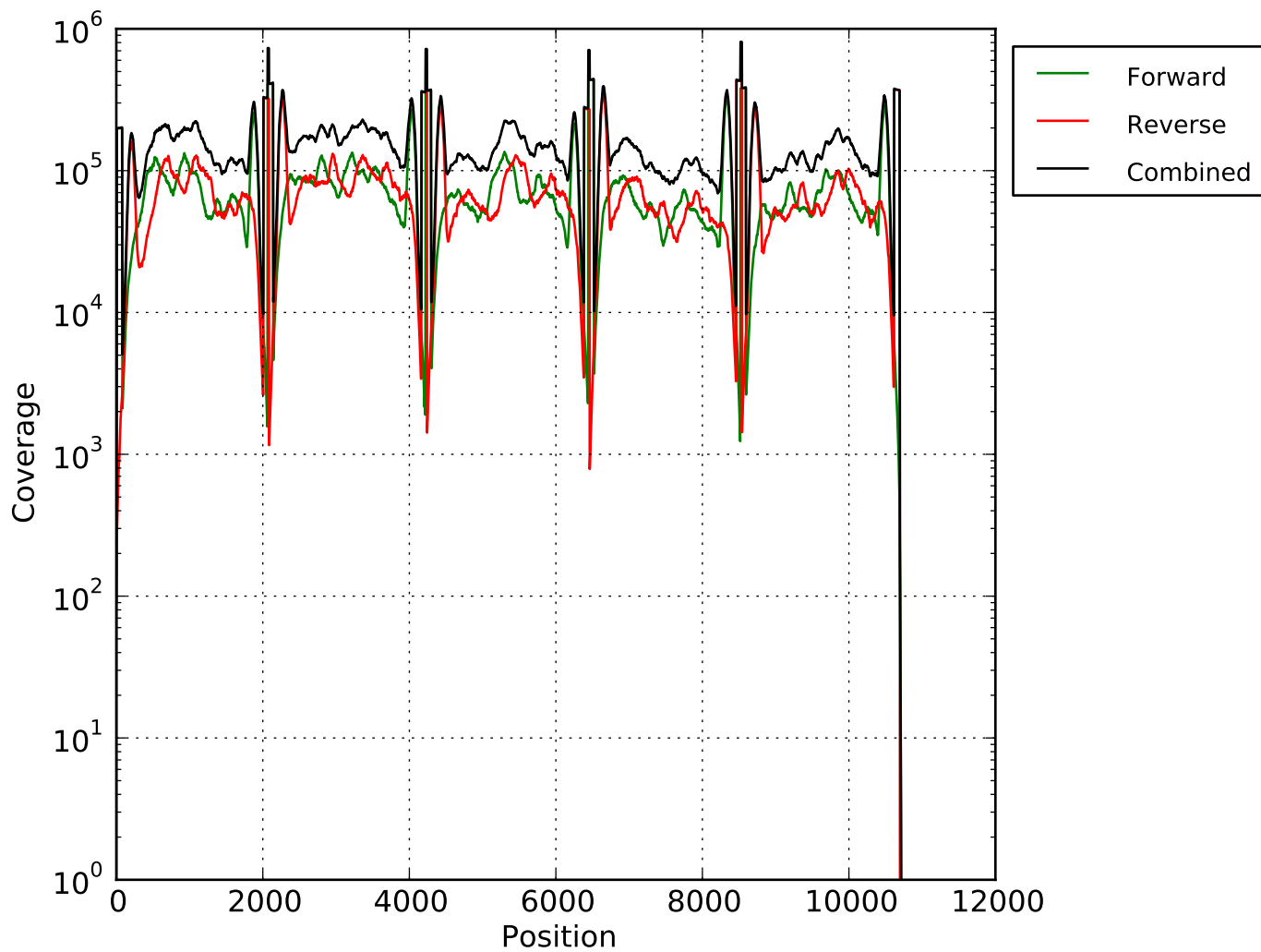

Supplement: S6 File — The LoFreq output.snp files for each sample in our data set are in the folder “SNP Files”. The read coverage graphs for each sample are in the folder “Coverage Plots”. (ZIP) [file pntd.0004044.s006.zip › S6_File/Coverage Plots/05K4441DK2-Aeg-Coverage.pdf]

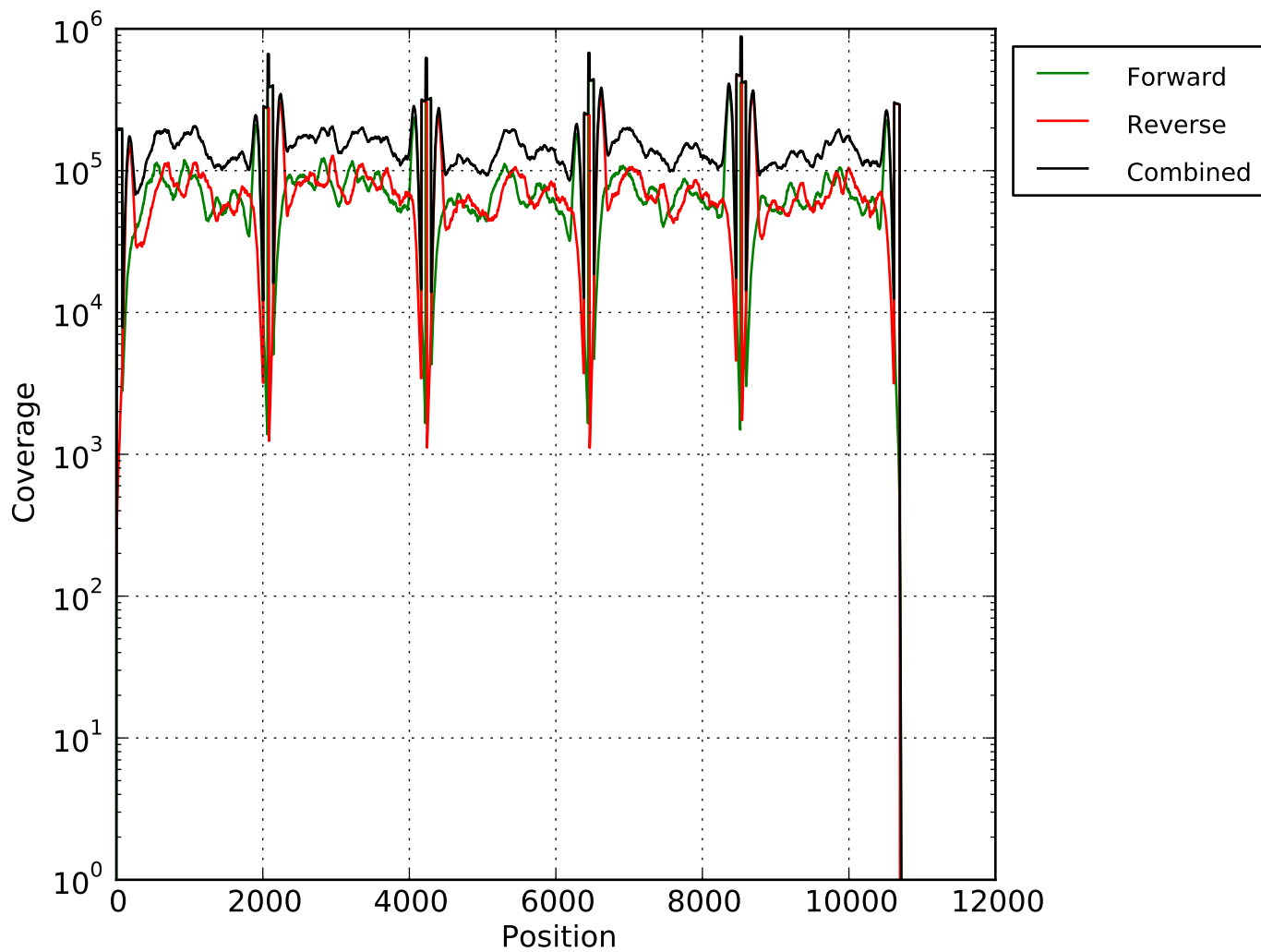

Supplement: S6 File — The LoFreq output.snp files for each sample in our data set are in the folder “SNP Files”. The read coverage graphs for each sample are in the folder “Coverage Plots”. (ZIP) [file pntd.0004044.s006.zip › S6_File/Coverage Plots/05K4441DK2-Albo-Coverage.pdf]

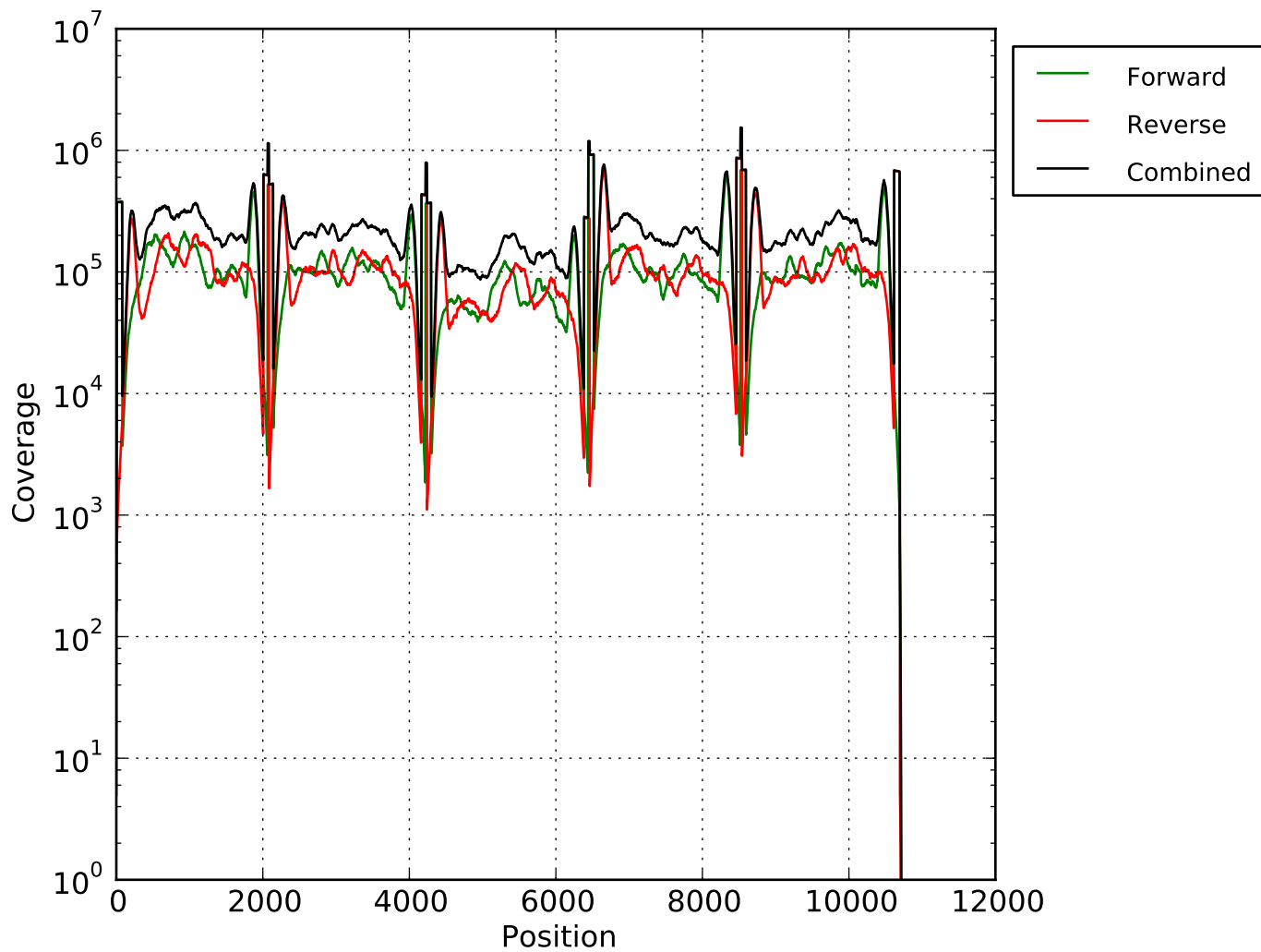

Supplement: S6 File — The LoFreq output.snp files for each sample in our data set are in the folder “SNP Files”. The read coverage graphs for each sample are in the folder “Coverage Plots”. (ZIP) [file pntd.0004044.s006.zip › S6_File/Coverage Plots/05K4441DK2-Serum-Coverage.pdf]

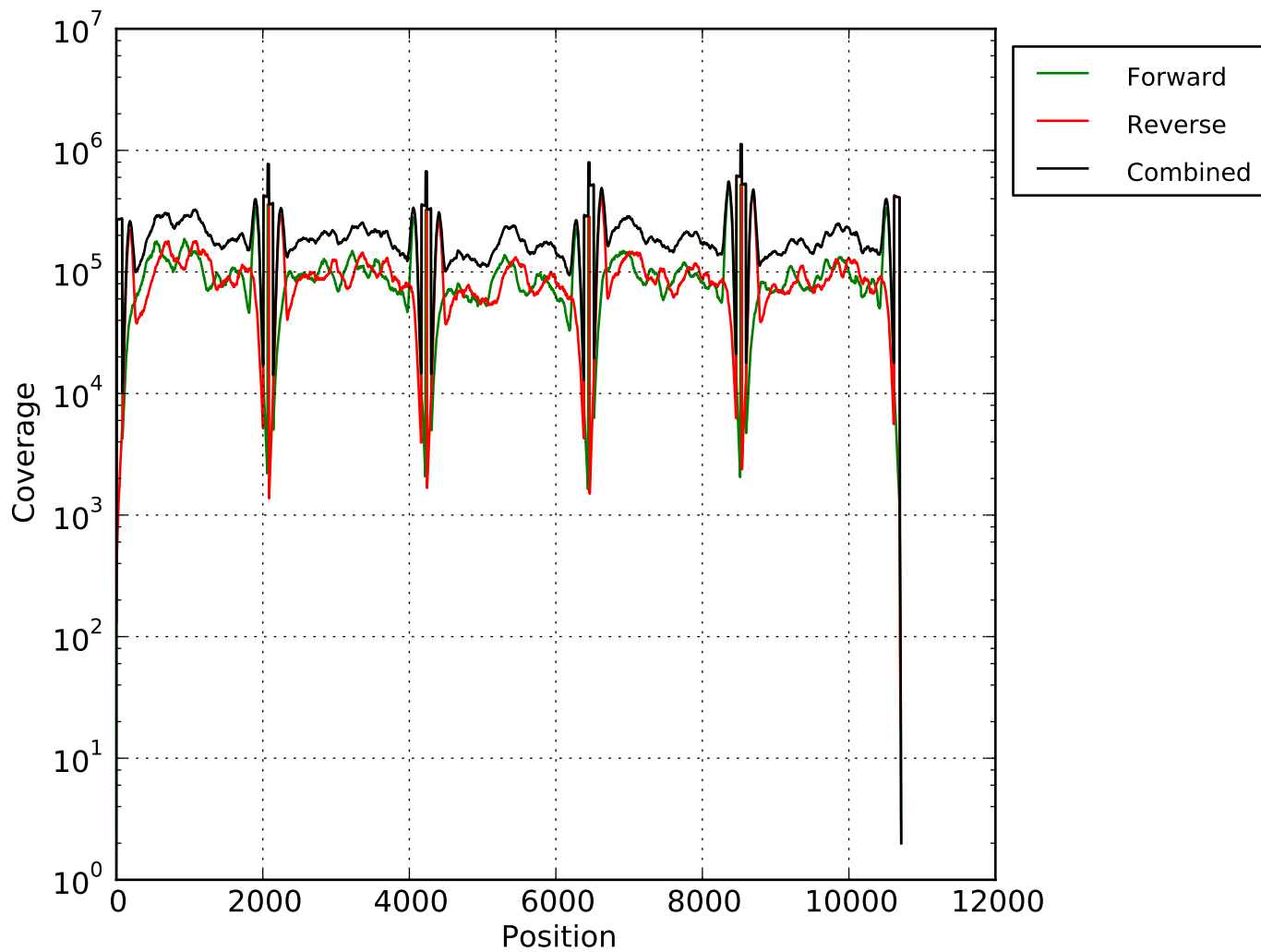

Supplement: S6 File — The LoFreq output.snp files for each sample in our data set are in the folder “SNP Files”. The read coverage graphs for each sample are in the folder “Coverage Plots”. (ZIP) [file pntd.0004044.s006.zip › S6_File/Coverage Plots/05K4468DK1-Aeg-Coverage.pdf]

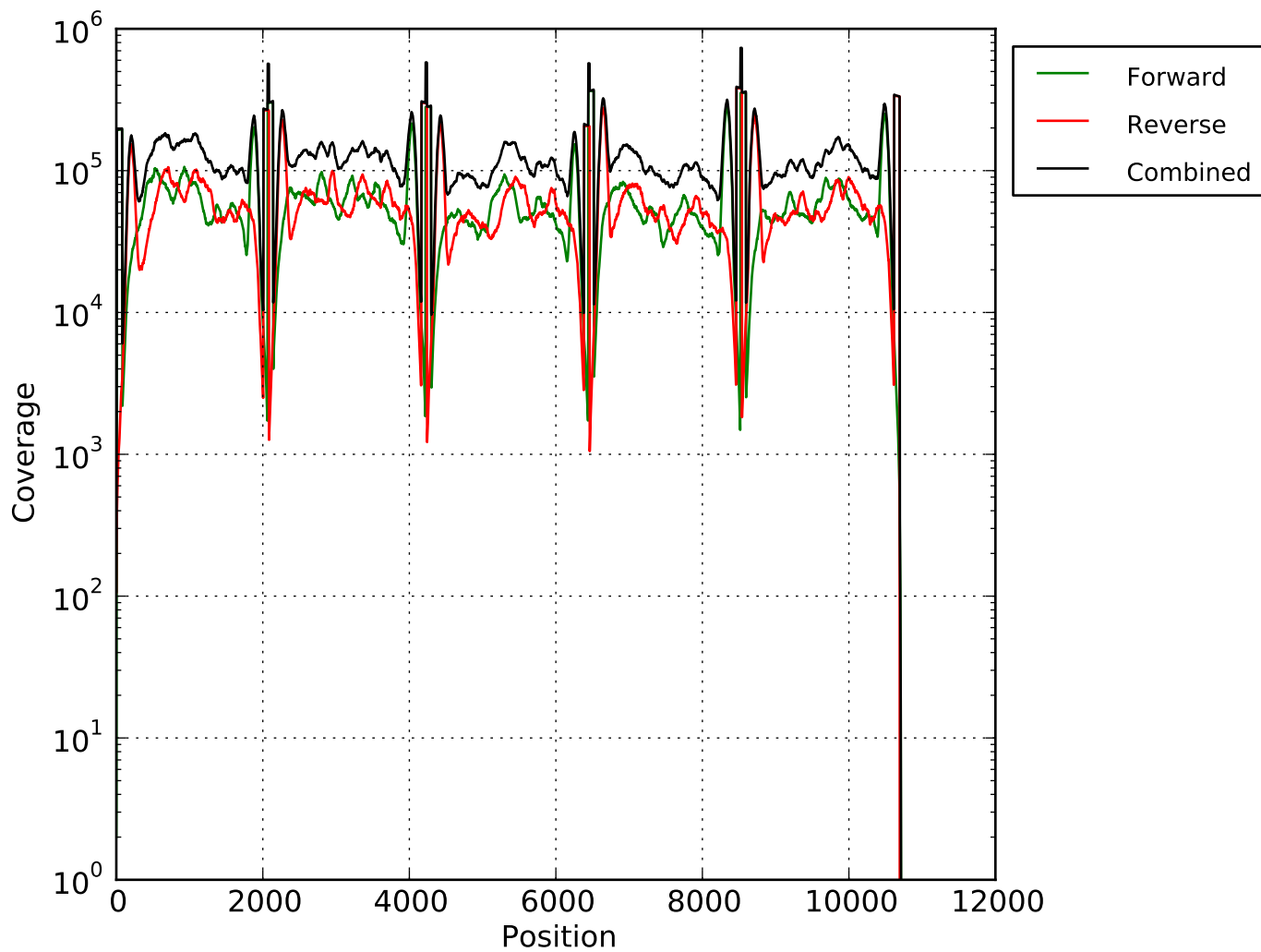

Supplement: S6 File — The LoFreq output.snp files for each sample in our data set are in the folder “SNP Files”. The read coverage graphs for each sample are in the folder “Coverage Plots”. (ZIP) [file pntd.0004044.s006.zip › S6_File/Coverage Plots/05K4468DK1-Serum-Coverage.pdf]

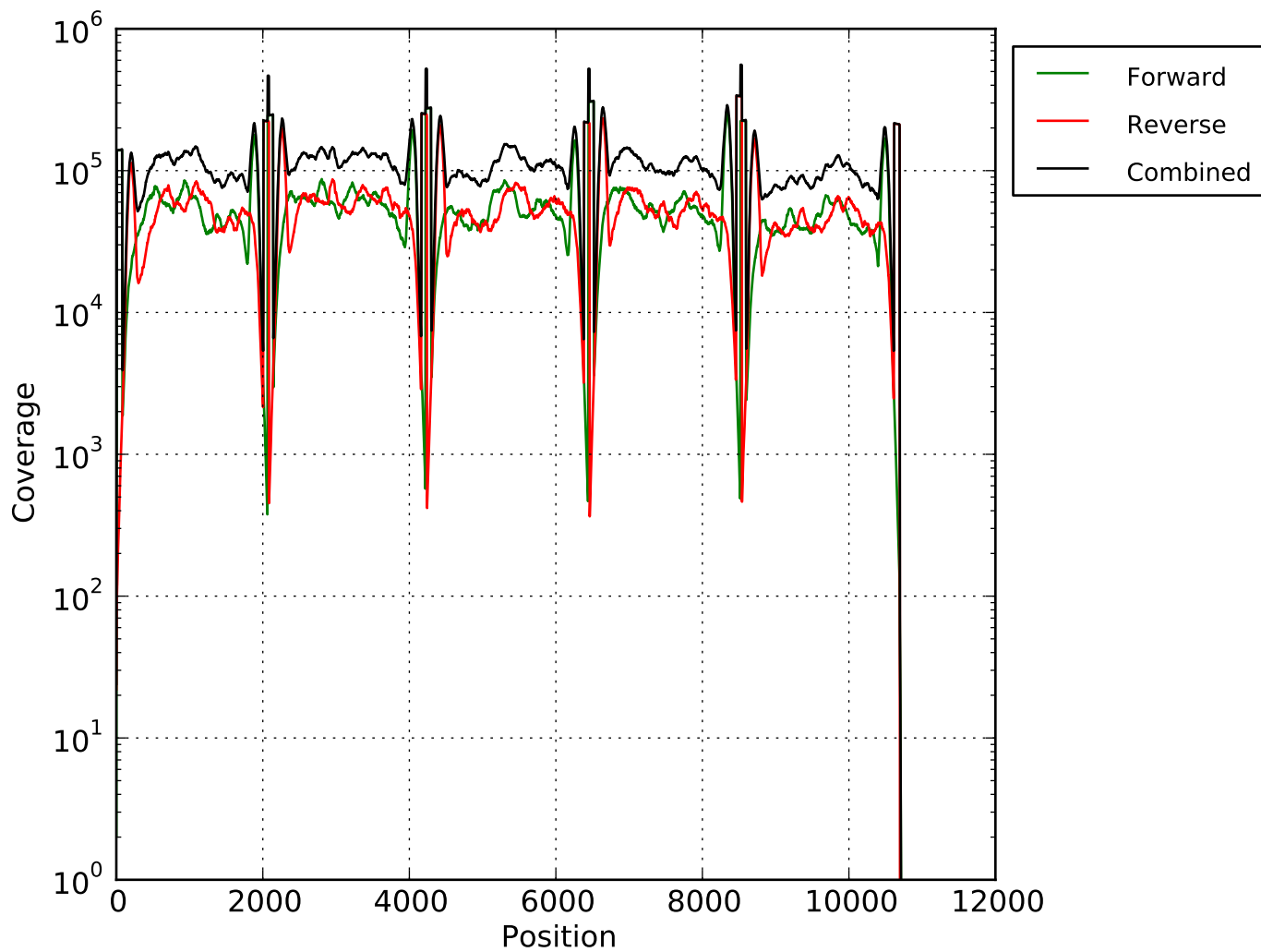

Supplement: S6 File — The LoFreq output.snp files for each sample in our data set are in the folder “SNP Files”. The read coverage graphs for each sample are in the folder “Coverage Plots”. (ZIP) [file pntd.0004044.s006.zip › S6_File/Coverage Plots/05K4468DK2-Aeg-Coverage.pdf]

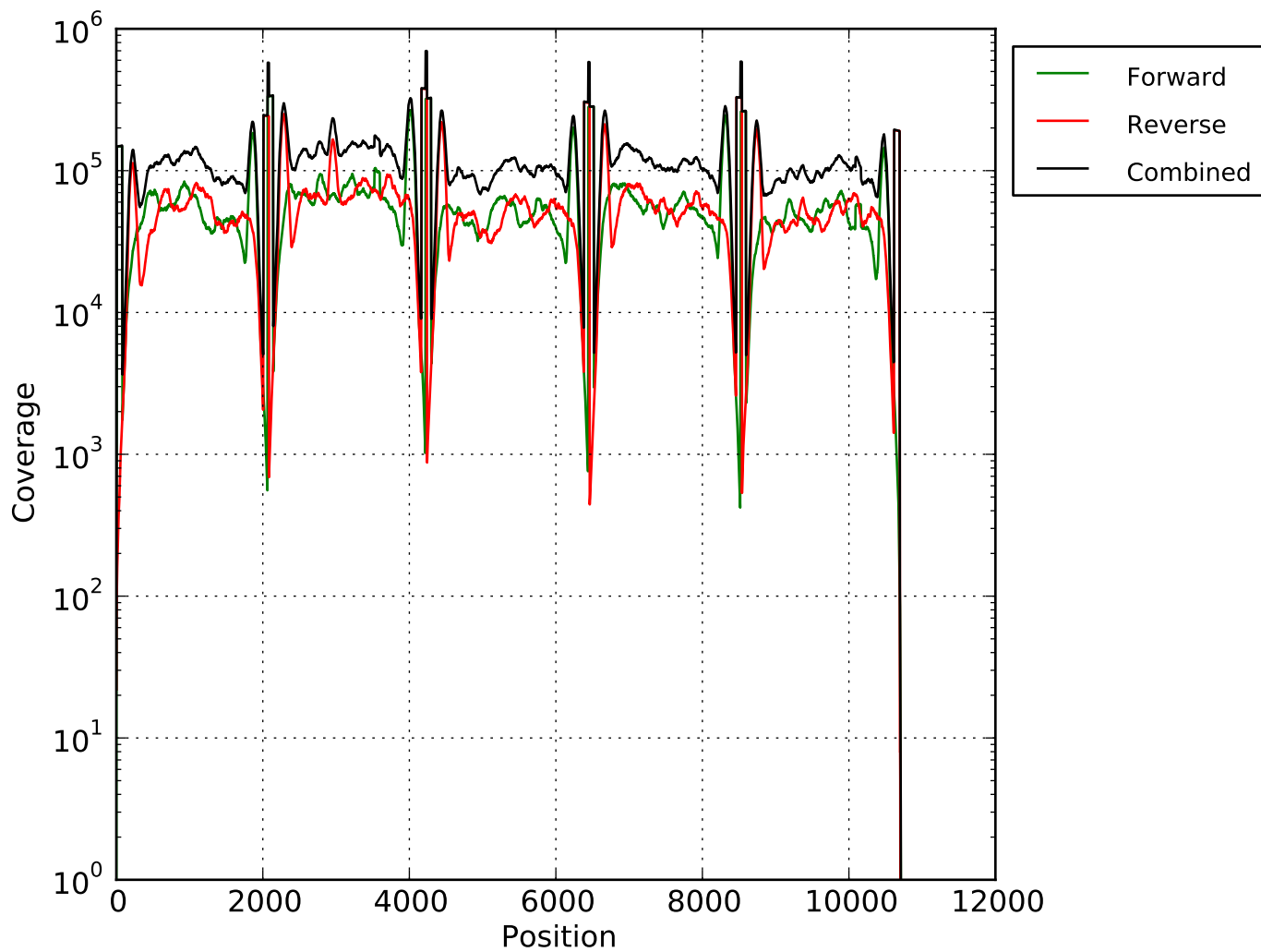

Supplement: S6 File — The LoFreq output.snp files for each sample in our data set are in the folder “SNP Files”. The read coverage graphs for each sample are in the folder “Coverage Plots”. (ZIP) [file pntd.0004044.s006.zip › S6_File/Coverage Plots/05K4468DK2-Albo-Coverage.pdf]

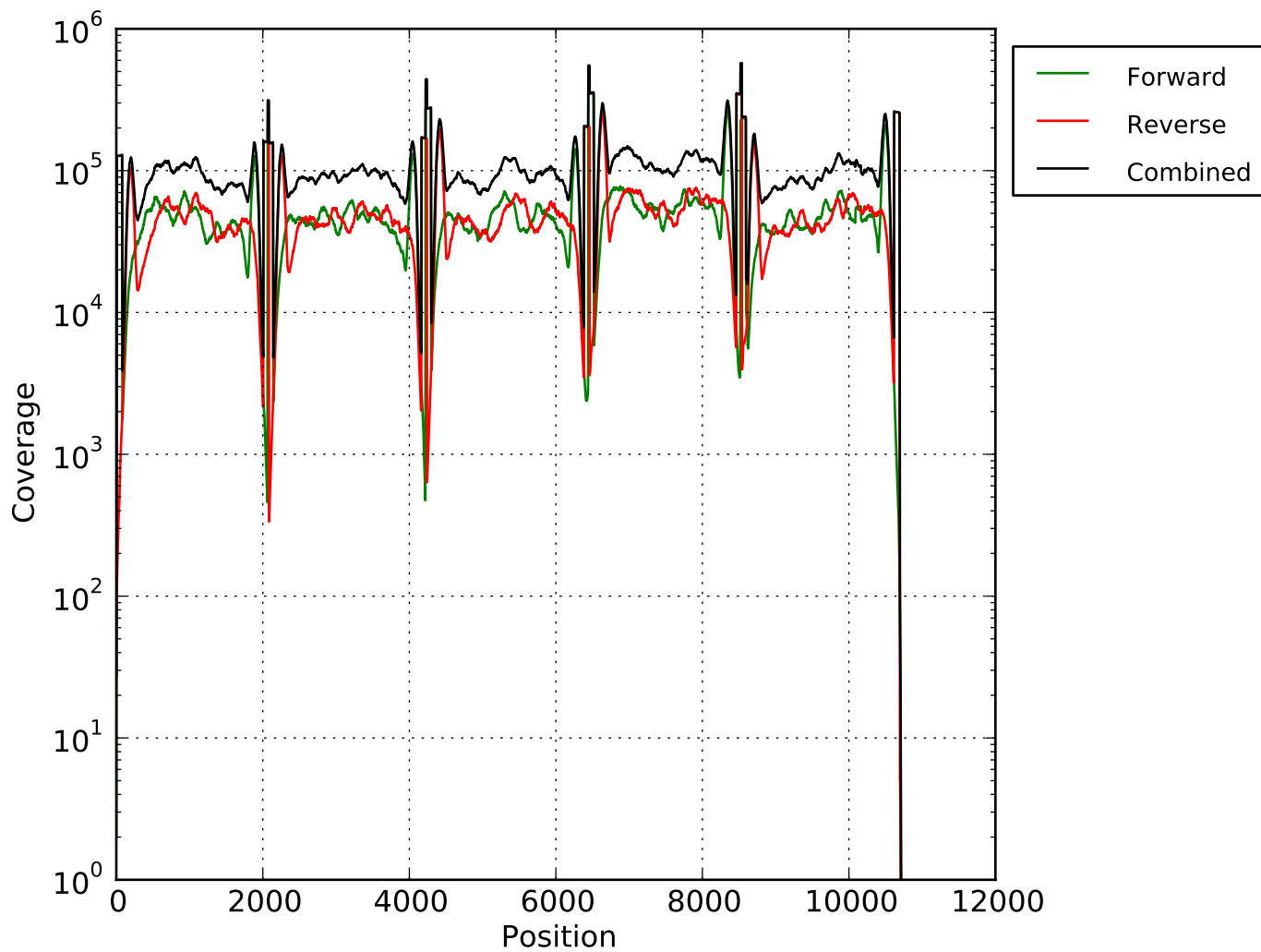

Supplement: S6 File — The LoFreq output.snp files for each sample in our data set are in the folder “SNP Files”. The read coverage graphs for each sample are in the folder “Coverage Plots”. (ZIP) [file pntd.0004044.s006.zip › S6_File/Coverage Plots/05K4468DK2-Serum-Coverage.pdf]

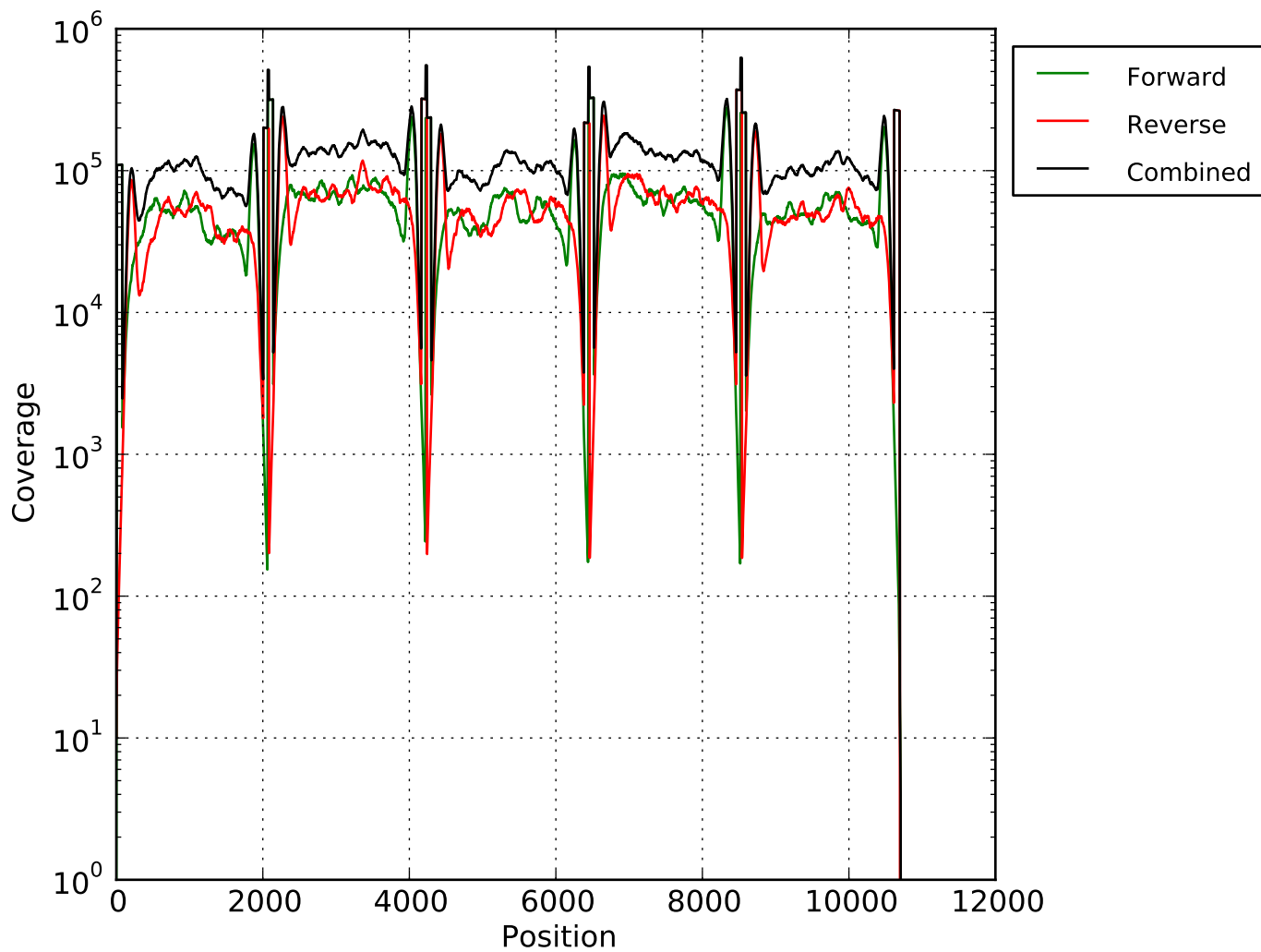

Supplement: S6 File — The LoFreq output.snp files for each sample in our data set are in the folder “SNP Files”. The read coverage graphs for each sample are in the folder “Coverage Plots”. (ZIP) [file pntd.0004044.s006.zip › S6_File/Coverage Plots/05K4621DK1-Aeg-Coverage.pdf]

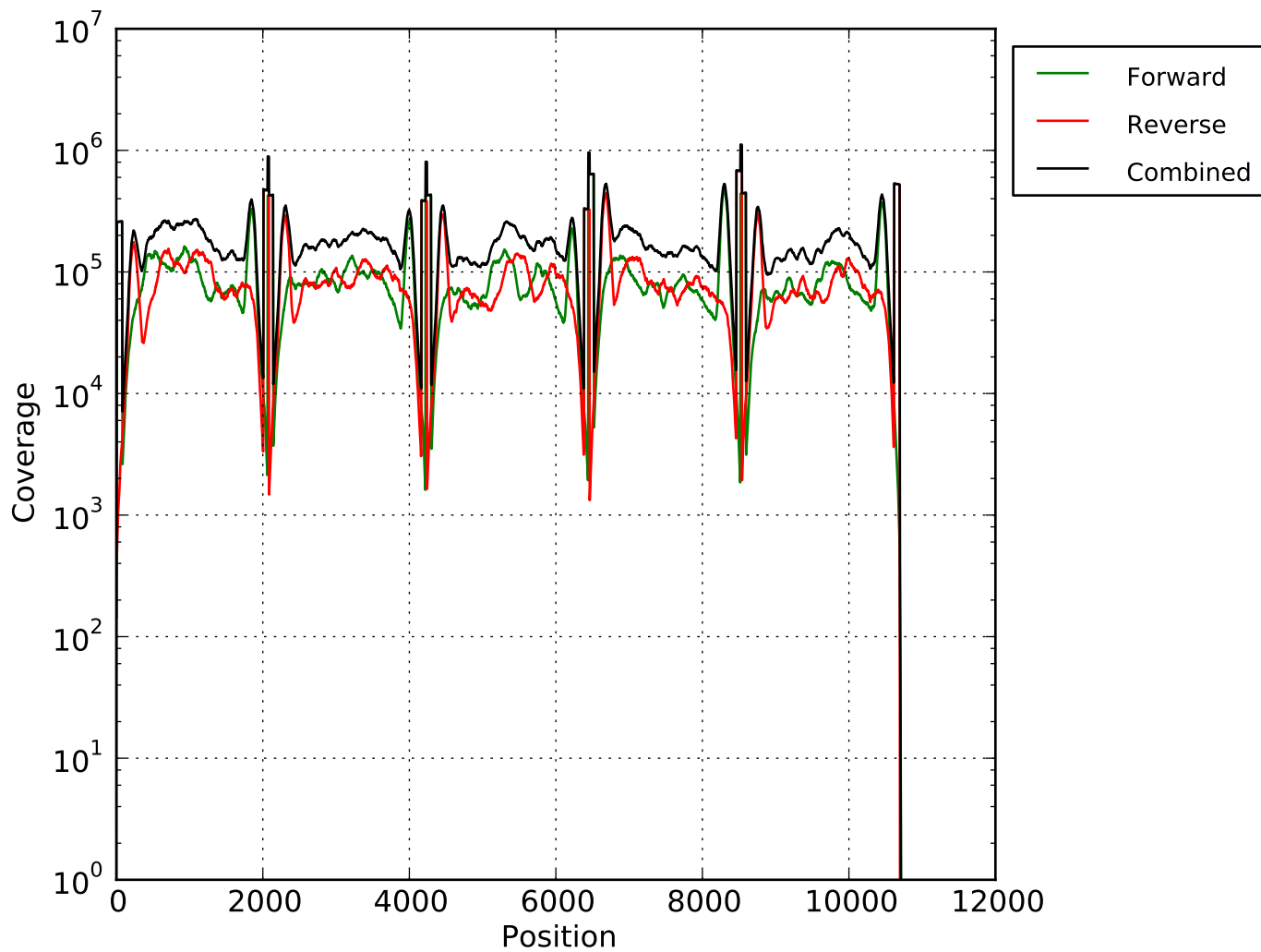

Supplement: S6 File — The LoFreq output.snp files for each sample in our data set are in the folder “SNP Files”. The read coverage graphs for each sample are in the folder “Coverage Plots”. (ZIP) [file pntd.0004044.s006.zip › S6_File/Coverage Plots/05K4621DK1-Albo-Coverage.pdf]

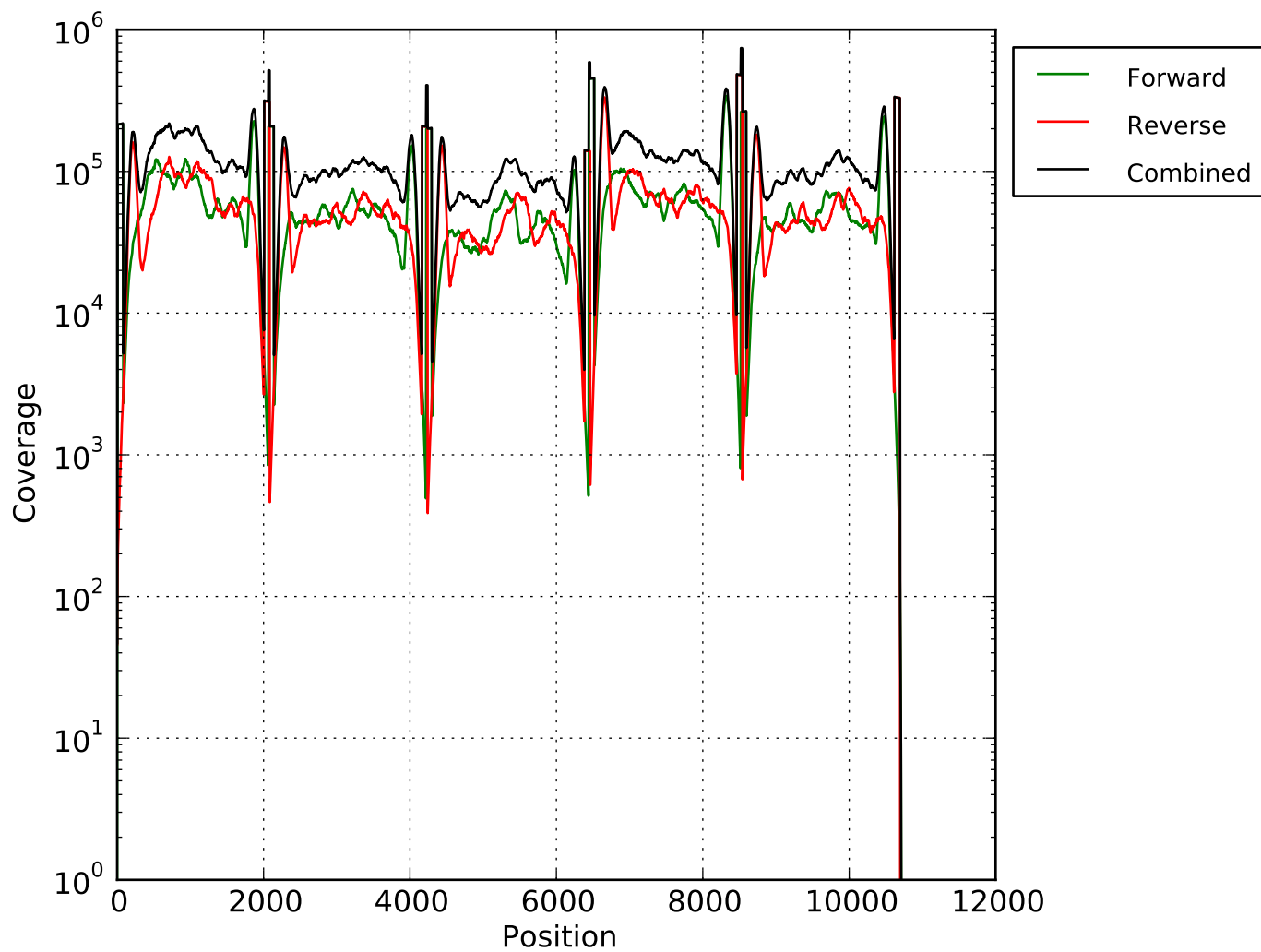

Supplement: S6 File — The LoFreq output.snp files for each sample in our data set are in the folder “SNP Files”. The read coverage graphs for each sample are in the folder “Coverage Plots”. (ZIP) [file pntd.0004044.s006.zip › S6_File/Coverage Plots/05K4621DK2-Aeg-Coverage.pdf]

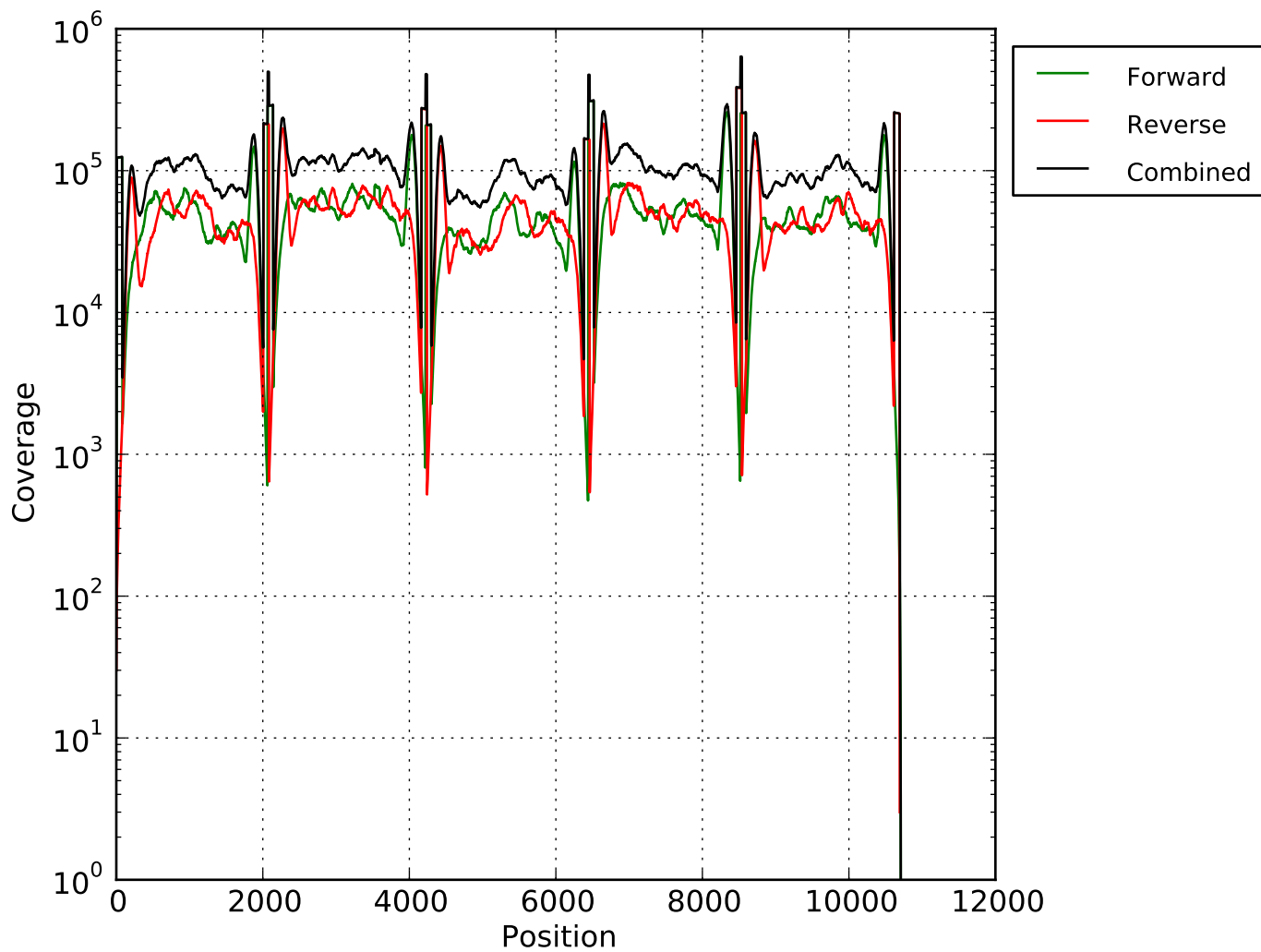

Supplement: S6 File — The LoFreq output.snp files for each sample in our data set are in the folder “SNP Files”. The read coverage graphs for each sample are in the folder “Coverage Plots”. (ZIP) [file pntd.0004044.s006.zip › S6_File/Coverage Plots/05K4621DK2-Albo-Coverage.pdf]

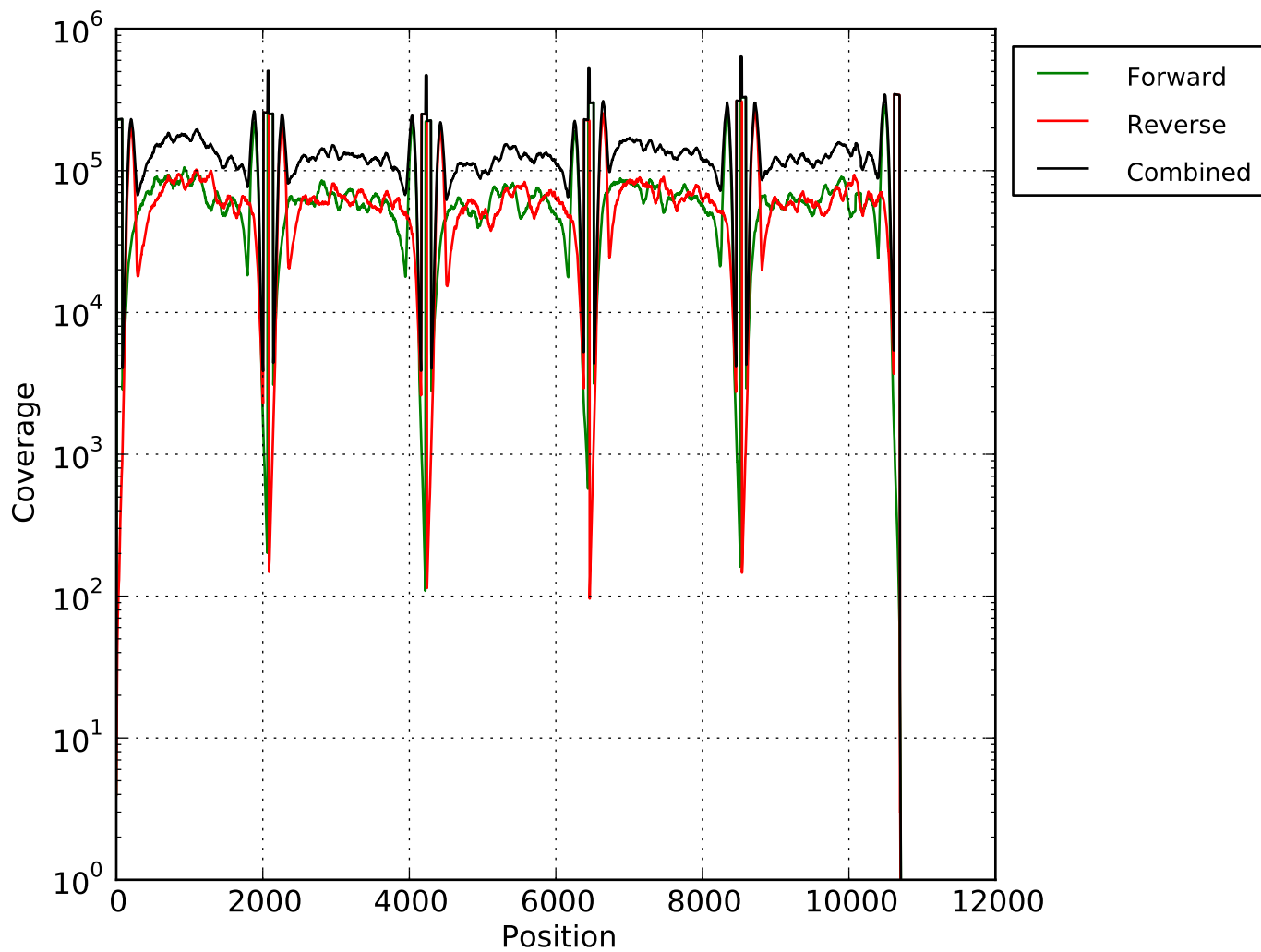

Supplement: S6 File — The LoFreq output.snp files for each sample in our data set are in the folder “SNP Files”. The read coverage graphs for each sample are in the folder “Coverage Plots”. (ZIP) [file pntd.0004044.s006.zip › S6_File/Coverage Plots/05K4622DK1-Aeg-Coverage.pdf]

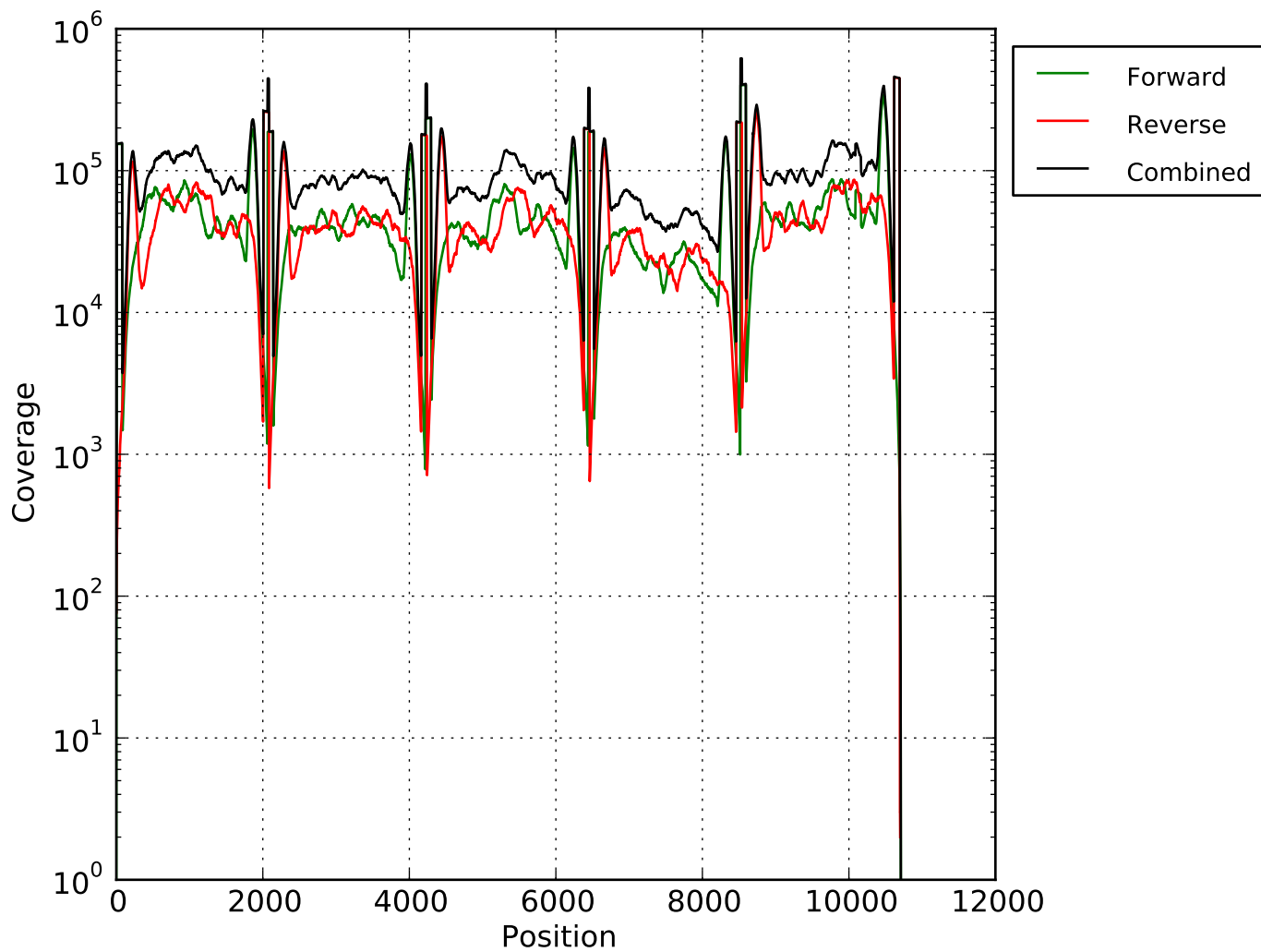

Supplement: S6 File — The LoFreq output.snp files for each sample in our data set are in the folder “SNP Files”. The read coverage graphs for each sample are in the folder “Coverage Plots”. (ZIP) [file pntd.0004044.s006.zip › S6_File/Coverage Plots/05K4622DK1-Albo-Coverage.pdf]

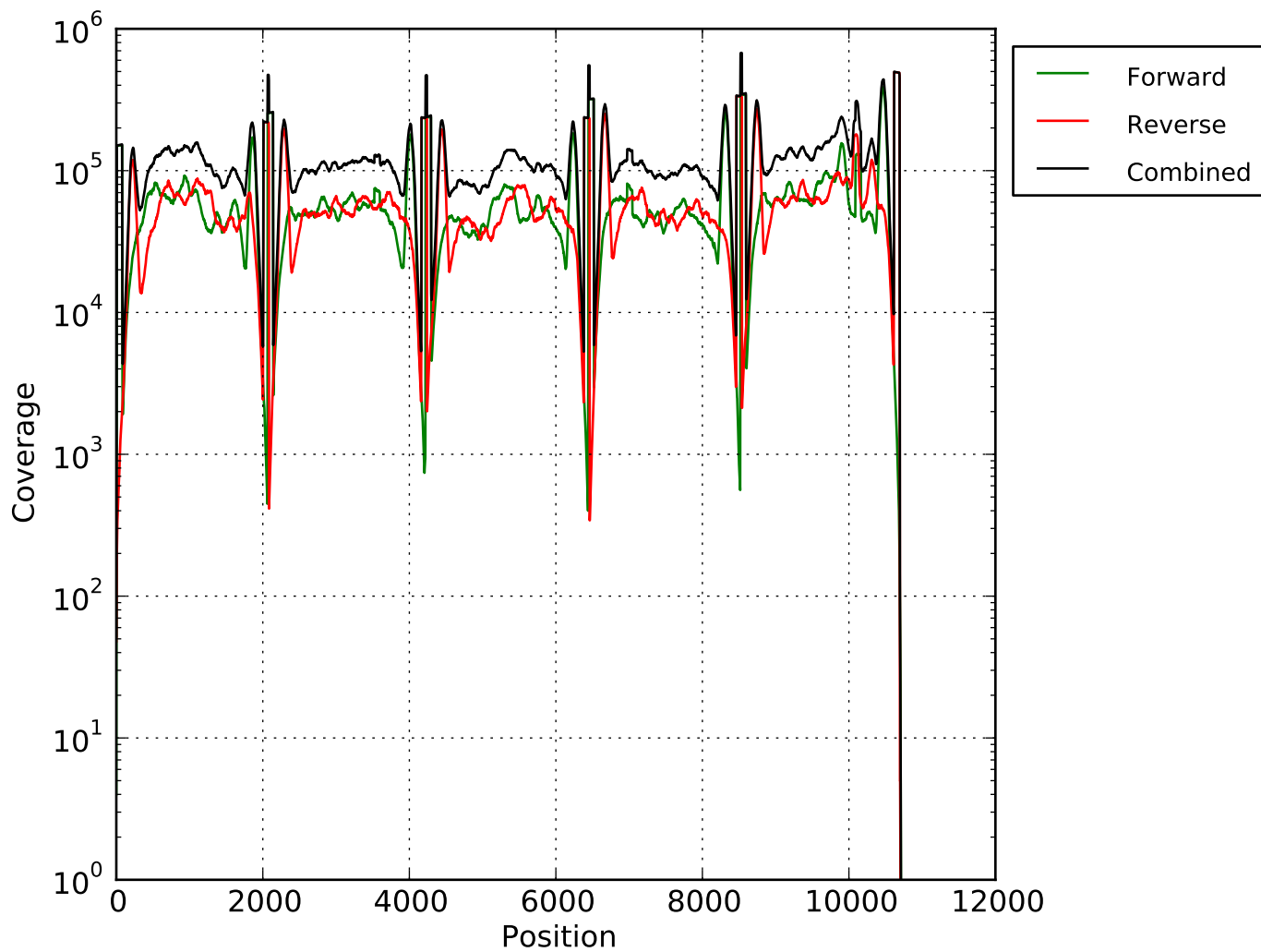

Supplement: S6 File — The LoFreq output.snp files for each sample in our data set are in the folder “SNP Files”. The read coverage graphs for each sample are in the folder “Coverage Plots”. (ZIP) [file pntd.0004044.s006.zip › S6_File/Coverage Plots/05K4622DK1-Serum-Coverage.pdf]

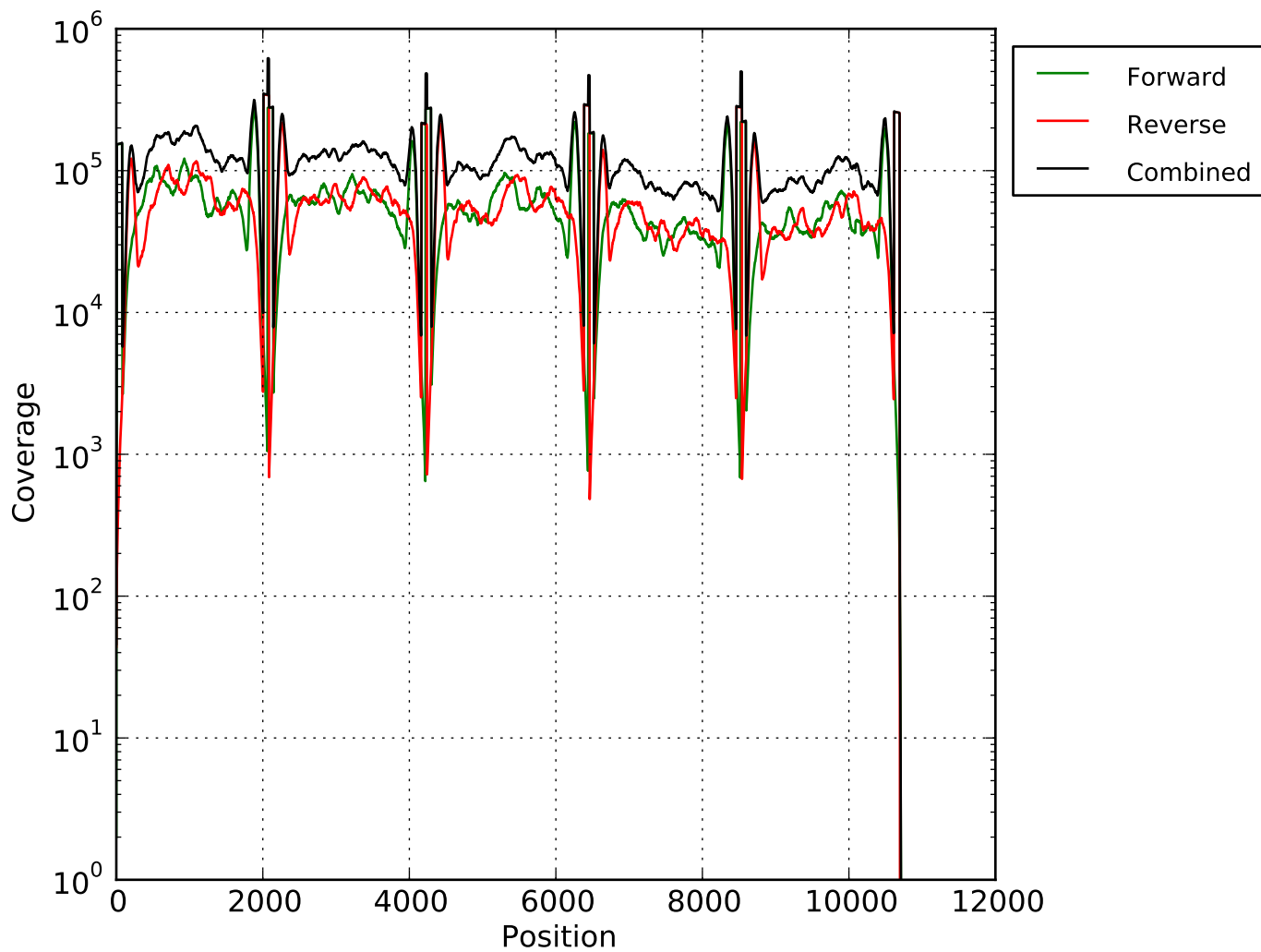

Supplement: S6 File — The LoFreq output.snp files for each sample in our data set are in the folder “SNP Files”. The read coverage graphs for each sample are in the folder “Coverage Plots”. (ZIP) [file pntd.0004044.s006.zip › S6_File/Coverage Plots/05K4622DK2-Aeg-Coverage.pdf]

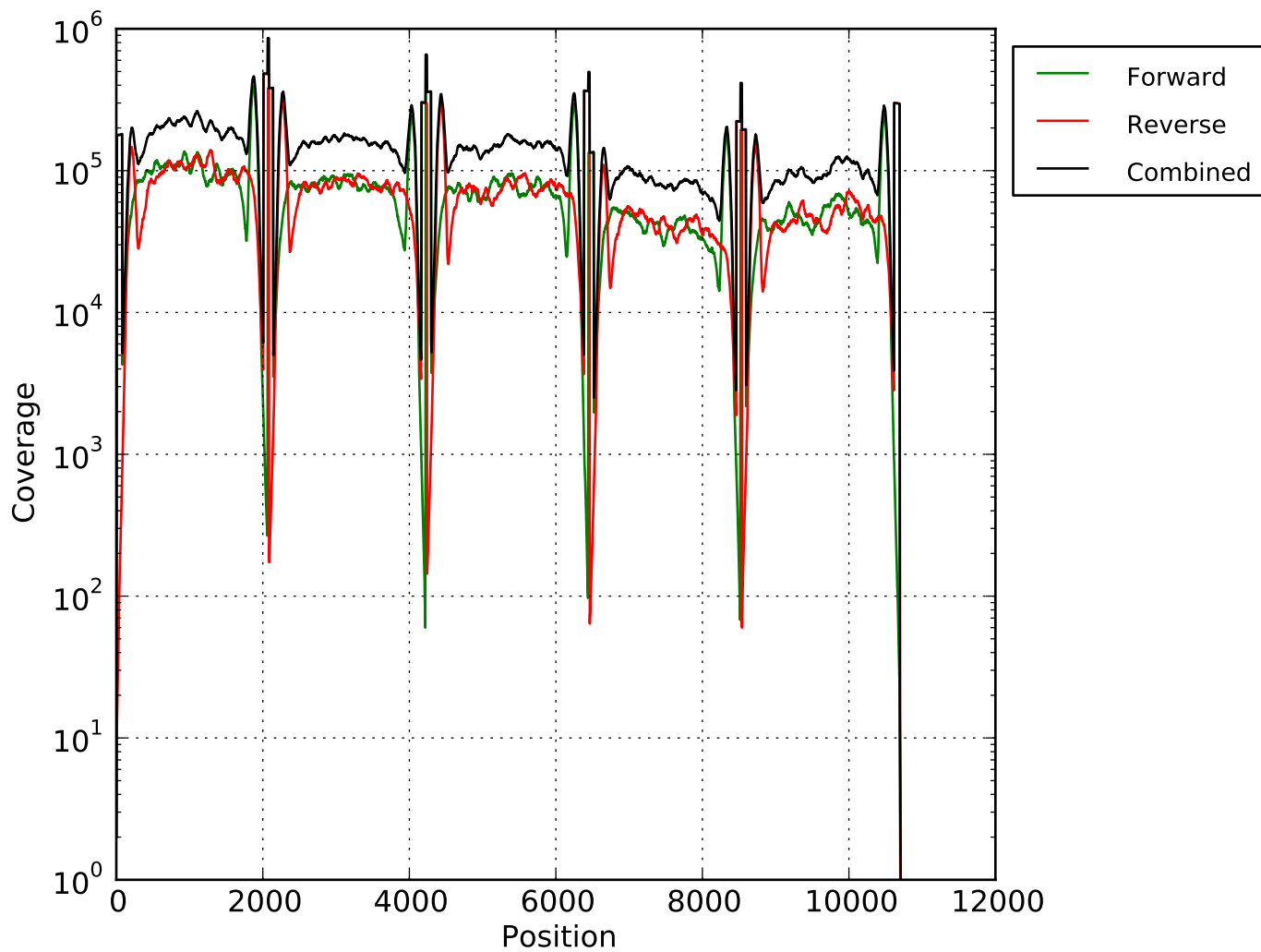

Supplement: S6 File — The LoFreq output.snp files for each sample in our data set are in the folder “SNP Files”. The read coverage graphs for each sample are in the folder “Coverage Plots”. (ZIP) [file pntd.0004044.s006.zip › S6_File/Coverage Plots/05K4622DK2-Albo-Coverage.pdf]

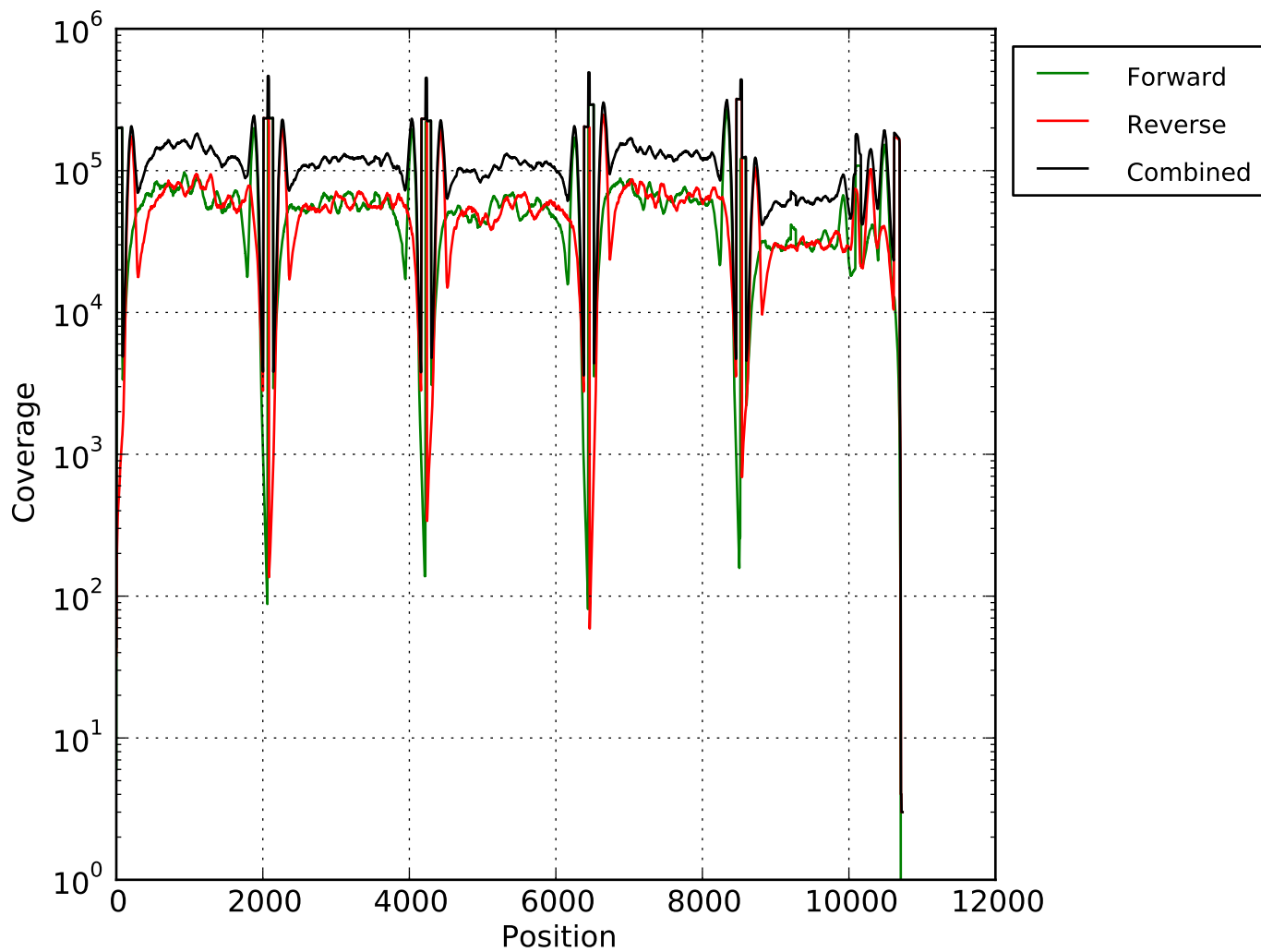

Supplement: S6 File — The LoFreq output.snp files for each sample in our data set are in the folder “SNP Files”. The read coverage graphs for each sample are in the folder “Coverage Plots”. (ZIP) [file pntd.0004044.s006.zip › S6_File/Coverage Plots/05K4622DK2-Serum-Coverage.pdf]
