# Supplementary material for: Analysis of Dengue Virus Genetic Diversity during Human and Mosquito Infection Reveals Genetic Constraints
Source: PLoS Negl Trop Dis. 2015 Sep 1;9(9):e0004044. doi: 10.1371/journal.pntd.0004044 (PMC4556638; doi:10.1371/journal.pntd.0004044)
Supplement: S7 File — Positions evidencing a change in consensus sequence in any of our samples were recorded and interrogated for each DENV-1 strain. Nucleotide sequences are color-coded and consensus is defined as >50% of reads mapping to the indicated position. Each of the circles depicted represents an experimental condition and are in the order of the experimental design figure depicted in Fig 1a. (PDF) [file pntd.0004044.s007.pdf]

S7 File

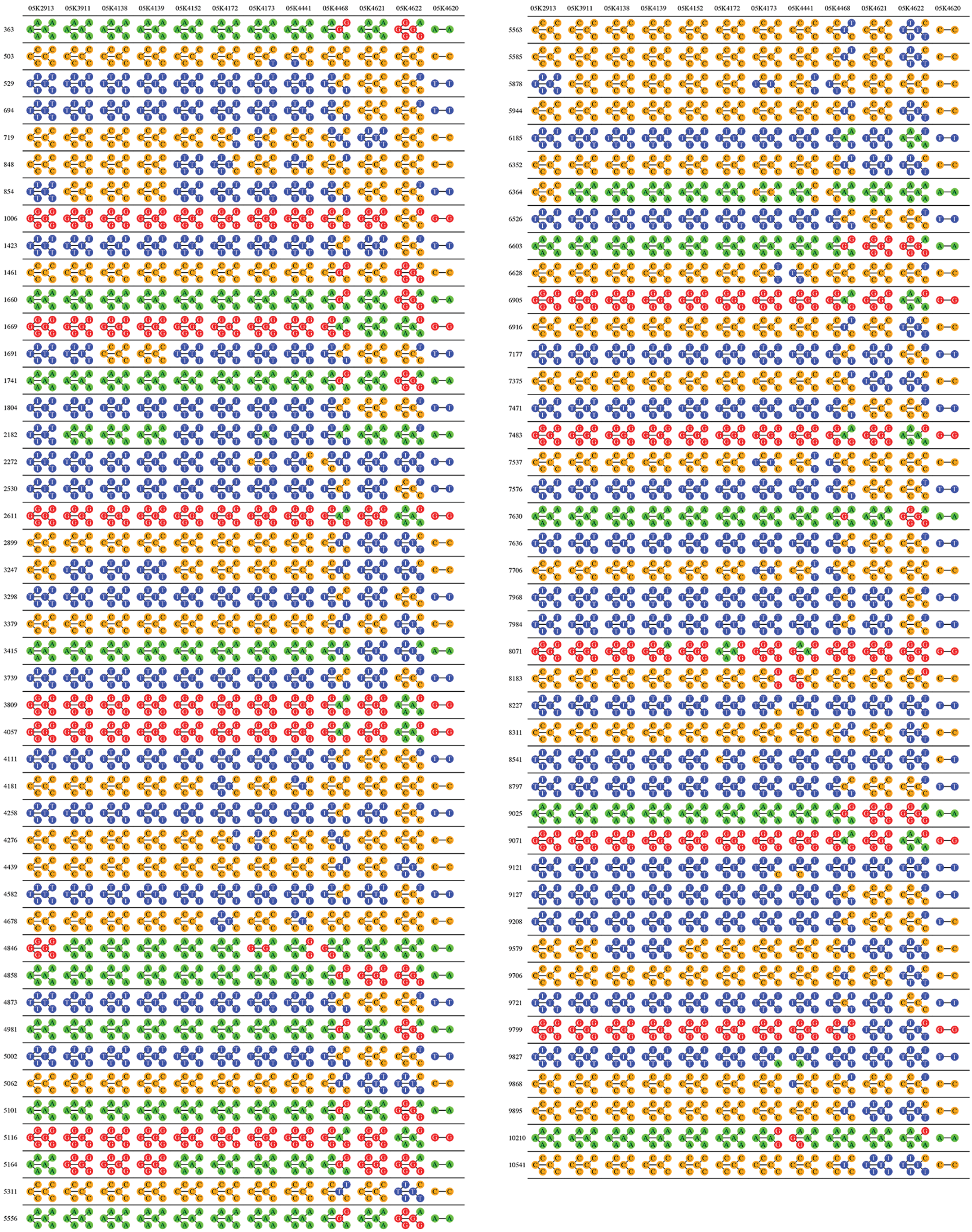
05K2913
05K3911
05K4138
05K4139
05K4152
05K4172
05K4173
05K4441
05K4468
05K4621
05K4622
05K4620
363
503
529
694
719
848
854
1006
1423
1461
1660
1669
1691
1741
1804
2182
2272
2530
2611
2899
3247
3298
3379
3415
3739
3809
4057
4111
4181
4258
4276
4439
4582
4678
4846
4858
4873
4981
5002
5062
5101
5116
5164
5311
5556
5563
5585
5878
5944
6185
6352
6364
6526
6603
6628
6905
6916
7177
7375
7471
7483
7537
7576
7630
7636
7706
7968
7984
8071
8183
8227
8311
8541
8797
9025
9071
9121
9127
9208
9579
9706
9721
9799
9827
9868
9895
10210
10541

Gene	Position	Consensus Changes	Amino acid change
C	363	A<->G	N<->S
prM	503	C->T	L->F
prM	529	T<->C	V<->V
prM	694	T<->C	H<->H
prM	719	C<->T	L<->L
prM	848	C<->T	L<->F
prM	854	C<->T	L<->L
E	1006	G<->C	V<->V
E	1423	T<->C	T<->T
E	1461	C<->G	T<->R
E	1660	A<->G	T<->T
E	1669	G<->A	A<->A
E	1691	T<->C	L<->L
E	1741	A<->G	E<->E
E	1804	T<->C	D<->D
E	2182	T<->A	G<->G
E	2272	T<->C	V<->V
NS1	2530	T<->C	D<->D
NS1	2611	G<->A	E<->E
NS1	2899	C<->T	F<->F
NS1	3247	C<->T	D<->D
NS1	3298	T<->C	N<->N
NS1	3379	C<->T	P<->P
NS1	3415	A<->T	G<->G
NS2A	3739	T<->C	A<->A
NS2A	3809	G<->A	A<->T
NS2A	4057	G<->A	V<->V
NS2A	4111	T<->C	N<->N
NS2B	4181	C<->T	L<->L
NS2B	4258	T<->C	V<->V
NS2B	4276	C<->T	A<->A
NS2B	4439	C<->T	L<->L
NS3	4582	T<->C	G<->G
NS3	4678	C<->T	T<->T
NS3	4846	G<->A	V<->V
NS3	4858	A<->G	P<->P
NS3	4873	T<->C	T<->T
NS3	4981	A<->G	V<->V
NS3	5002	T<->C	Y<->Y
NS3	5062	C<->T	D<->D
NS3	5101	A<->G	L<->L
NS3	5116	G<->A	G<->G
NS3	5164	A<->G	R<->R
NS3	5311	C<->T	A<->A
NS3	5556	A<->G	N<->S
NS3	5563	C<->T	G<->G
NS3	5585	C<->T	P<->S
NS3	5878	T<->C	A<->A
NS3	5944	C<->T	Y<->Y
NS3	6185	T<->A	F<->I
NS3	6352	C<->T	F<->F
NS3	6364	C<->A	A<->A

NS4A	6526	T<->C	D<->D
NS4A	6603	A<->G	K<->R
NS4B	6628	C<->T	G<->G
NS4B	6905	G<->A	A<->T
NS4B	6916	C<->T	D<->D
NS4B	7177	T<->C	A<->A
NS4B	7375	C<->T	L<->L
NS4B	7471	T<->C	N<->N
NS4B	7483	G<->A	A<->A
NS4B	7537	C<->T	A<->A
NS5	7576	T<->C	G<->G
NS5	7630	A<->G	Q<->Q
NS5	7636	T<->C	S<->S
NS5	7706	C<->T	L<->L
NS5	7968	T<->C	V<->A
NS5	7984	T<->C	P<->P
NS5	8071	G<->A	K<->K
NS5	8183	C<->G	L<->V
NS5	8227	T->C	Y->Y
NS5	8311	C<->T	H<->H
NS5	8541	C->T	A->V
NS5	8797	T<->C	I<->I
NS5	9025	A<->G	E<->E
NS5	9071	G<->A	E<->K
NS5	9121	T->C	Y->Y
NS5	9208	T<->C	D<->D
NS5	9579	C<->T	T<->I
NS5	9706	C<->T	H<->H
NS5	9721	T<->C	I<->I
NS5	9799	G<->T	G<->G
NS5	9827	T->A	C->S
NS5	9868	C<->T	Y<->Y
NS5	9895	C<->T	A<->A
NS5	10210	A<->G	L<->L
utr	10541	C<->T	Q<->*

S7 File. Consensus shifts over time by position. Positions evidencing a change in consensus sequence in any of our samples were recorded and interrogated for each DENV-1 strain. Nucleotide sequences are color-coded and consensus is defined as >50% of reads mapping to the indicated position. Each of the circles depicted represents an experimental condition and are in the order of the experimental design figure depicted in Figure 1a. The nucleotide changes and the amino acid change (if any) of the positions depicted graphically are summarized in the table below.
