# Supplementary material for: Analysis of Dengue Virus Genetic Diversity during Human and Mosquito Infection Reveals Genetic Constraints
Source: PLoS Negl Trop Dis. 2015 Sep 1;9(9):e0004044. doi: 10.1371/journal.pntd.0004044 (PMC4556638; doi:10.1371/journal.pntd.0004044)
Supplement: S10 File — The ratio of the mean nonsynonymous and synonymous mutations were calculated for the polyprotein as well as for each protein coding sequence. (PDF) [file pntd.0004044.s010.pdf]

S10 File

dN/dS-Mean

	Polyprotein	C	prM	E	NS1	NS2A	NS2B	NS3	NS4A	2K protein	NS4B	NS5
**Early Aegypti**	0.163	0.095	0.063	0.237	0.159	0.101	0.358	0.119	0.214	0.336	0.205	0.3
**Late Aegypti**	0.126	0.061	0.204	0.207	0.127	0.116	0	0.13	0.08	0.336	0.296	0.148
**Early Albopictus**	0.176	0.041	0.186	0.233	0.215	0.288	0.243	0.147	0.181	0.168	0.238	0.141
**Late Albopictus**	0.149	0.086	0.19	0.243	0.12	0.06	0.064	0.149	0	0.168	0.018	0.216
**Early Human**	0.183	0.143	0.171	0.189	0.174	0.346	0.099	0.151	0.12	0.202	0.198	0.164
**Late Human**	0.225	0.071	0.188	0.186	0.385	0.26	0.107	0.144	0.25	0.112	0.193	0.232

S10 File. dN/dS analysis. The ratio of the mean nonsynonymous and synonymous mutations were calculated for the polyprotein as well as for each protein coding sequence.
