# Supplementary material for: Analysis of Dengue Virus Genetic Diversity during Human and Mosquito Infection Reveals Genetic Constraints
Source: PLoS Negl Trop Dis. 2015 Sep 1;9(9):e0004044. doi: 10.1371/journal.pntd.0004044 (PMC4556638; doi:10.1371/journal.pntd.0004044)
Supplement: S11 File — The Mann-Whitney test was used to compare individual dN/dS values for each protein coding sequence to the other protein coding sequences within each experimental condition. (PDF) [file pntd.0004044.s011.pdf]

S11 File

Mann-Whitney test dN/dS values between protein coding sequences

	C	prM	E	NS1	NS2A	NS2B	NS3	NS4A	2K protein	NS4B	NS5
**Early Aegypti**	0.443	0.082	0.536	0.713	0.243	0.414	0.117	0.971	0.27	0.522	0.043
**Late Aegypti**	0.096	0.49	0.576	0.799	0.811	0.002	0.965	0.222	0.024	0.102	0.444
**Early Albopictus**	0.024	0.772	0.562	0.4	0.626	0.476	0.868	0.843	0.968	0.94	0.979
**Late Albopictus**	0.461	0.944	0.037	0.989	0.116	0.142	0.498	0.119	0.487	0.086	0.04
**Early Human**	0.557	0.526	0.316	0.635	0.568	0.351	0.636	0.84	0.349	0.662	0.399
**Late Human**	0.235	0.873	0.957	0.233	0.061	0.225	0.435	0.696	0.547	0.238	0.735

S11 File. Mann-Whitney test for significant dN/dS differences between protein coding sequences. The Mann-Whitney test was used to compare individual dN/dS values for each protein coding sequence to the other protein coding sequences within each experimental condition
