# Supplementary material for: Analysis of Dengue Virus Genetic Diversity during Human and Mosquito Infection Reveals Genetic Constraints
Source: PLoS Negl Trop Dis. 2015 Sep 1;9(9):e0004044. doi: 10.1371/journal.pntd.0004044 (PMC4556638; doi:10.1371/journal.pntd.0004044)
Supplement: S12 File — Transition (Ts) is the mutation between the same nucleotide type (A ↔ G, C ↔ T) and transversion (Tv) is mutation that results in change in nucleotide type (A↔ C, G ↔ T, G ↔ C, A ↔ T). The ratio of these mutation types is calculated here. (PDF) [file pntd.0004044.s012.pdf]

**S12 File**

*Mean Transition/Transversion ratio*

| | Polyprotein | C | prM | E | NS1 | NS2A | NS2B | NS3 | NS4A | 2K protein | NS4B | NS5 |
|---|---|---|---|---|---|---|---|---|---|---|---|---|
| **Early Aegypti** | 0.741 | 1.000 | 1.000 | 0.876 | 0.954 | 0.960 | 1.000 | 0.856 | 1.000 | 1.000 | 0.939 | 0.703 |
| **Late Aegypti** | 9.782 | 1.000 | No Tv | 4.750 | 1.750 | 2.600 | 1.000 | 4.600 | 0.667 | No Tv | 2.000 | 6.357 |
| **Early Albopictus** | 0.575 | 1.000 | 1.000 | 0.779 | 0.930 | 0.951 | 0.939 | 0.799 | 0.932 | 1.000 | 1.000 | 0.879 |
| **Late Albopictus** | 5.775 | No Tv | 1.667 | 4.517 | 3.500 | 2.900 | 1.750 | 3.313 | 0.600 | No Tv | 2.250 | 6.646 |
| **Early Human** | 0.706 | 1.000 | 0.952 | 0.647 | 0.950 | 0.890 | 0.939 | 0.909 | 0.948 | 1.000 | 0.899 | 0.821 |
| **Late Human** | 4.511 | 1.000 | 2.333 | 4.056 | 3.233 | 1.875 | 0.000 | 2.167 | 0.000 | 0.333 | 1.417 | 3.684 |

**S12 File. Transition/Transversion ratio.** Transition (Ts) is the mutation between the same nucleotide type (A ↔ G, C ↔ T) and transversion (Tv) is mutation that results in change in nucleotide type (A↔ C, G ↔ T, G ↔ C, A ↔ T). The ratio of these mutation types is calculated here.
