# Supplementary material for: Analysis of Dengue Virus Genetic Diversity during Human and Mosquito Infection Reveals Genetic Constraints
Source: PLoS Negl Trop Dis. 2015 Sep 1;9(9):e0004044. doi: 10.1371/journal.pntd.0004044 (PMC4556638; doi:10.1371/journal.pntd.0004044)
Supplement: S13 File — The number of mutations in a 100bp window were calculated across the genome. Diversity and evenness of the nucleotide type change (A ↔ G, C ↔ T, A↔ C, G ↔ T, G ↔ C, A ↔ T) were calculated using the Shannon diversity index and Shannon equitability measurement respectively. (PDF) [file pntd.0004044.s013.pdf]

S13 File

Mutation rate, diversity and eveness across the polyprotein

	# Snps per 100bp	Shannon diversity index	Shannon equitability
**Early Aegypti**	0.397	1.098	0.187
**Early Albopictus**	0.501	1.22	0.2
**Early Human**	0.438	1.314	0.22
**Late Aegypti**	0.536	1.071	0.173
**Late Albopictus**	0.406	1.152	0.195
**Late Human**	0.566	1.285	0.206

S13 File. Mutation rate, diversity and eveness across the polyprotein. The number of mutations in a 100bp window were calculated across the genome. Diversity and evenness of the nucleotide type change (A ↔ G, C ↔ T, A↔ C, G ↔ T, G ↔ C, A ↔ T) were calculated using the Shannon diversity index and Shannon equitability measurement respectively
