# Supplementary material for: Analysis of Dengue Virus Genetic Diversity during Human and Mosquito Infection Reveals Genetic Constraints
Source: PLoS Negl Trop Dis. 2015 Sep 1;9(9):e0004044. doi: 10.1371/journal.pntd.0004044 (PMC4556638; doi:10.1371/journal.pntd.0004044)
Supplement: S14 File — The dN/dS and Ts/Tv ratios for the polyprotein across experimental conditions were compared using the non-parametric Mann-Whitney-Wilcoxon test. Significant results are depicted in bold. (PDF) [file pntd.0004044.s014.pdf]

S14 File

Mann-Whitney-Wilcoxon test

	p value - dN/dS	p valule - Ts/Tv
**Aegypti: Early vs Late**	**0.042**	**0.004**
**Albopictus: Early vs Late**	0.465	**0.001**
**Human: Early vs Late**	0.413	**0.001**
**Early: Aegypti vs Albopictus**	0.638	0.083
**Early: Human vs Aegypti**	0.765	1
**Early: Human vs Albopictus**	0.898	0.413
**Late: Aegypti vs Albopictus**	0.765	**0.024**
**Late: Human vs Aegypti**	0.054	**0.019**
**Late: Human vs Albopictus**	0.123	0.123

S14 File. Mann-Whitney-Wilcoxon test. The dN/dS and Ts/Tv ratios for the polyprotein across experimental conditions were compared using the non-parametric Mann-Whitney-Wilcoxon test. Significant results are depicted in bold.
